# Supplementary material for: Contact-separation-induced self-recoverable mechanoluminescence of CaF2:Tb3+/PDMS elastomer
Source: Nat Commun. 2024 Mar 5;15:2014. doi: 10.1038/s41467-024-46432-3 (PMC10914845; doi:10.1038/s41467-024-46432-3)
Supplement: Supplementary file 1 — Supplementary Information [file 41467_2024_46432_MOESM1_ESM.pdf]

Supplementary Information

Contact-separation-induced self-recoverable mechanoluminescence of CaF_2:Tb^{3+}/PDMS elastomer

Wenxiang Wang, Shanwen Wang, Yan Gu, Jinyu Zhou, Jiachi Zhang

National & Local Joint Engineering Laboratory for Optical Conversion Materials and Technology, Lanzhou University, Lanzhou 730000, P. R. China

Content

Supplementary Note 1: Why do we focus on centrosymmetric phosphors? 1

Supplementary Note 2: Microstructure and elemental composition of CaF_2 5

Supplementary Note 3: Charge compensation mechanism.. 20

Supplementary Note 4: Spectra of CaF_2: Tb^{3+}/Eu^{2+}/Sm^{3+} 22

Supplementary Note 5: Two-dimensional stretching machine 30

Supplementary Note 6: Sample preparation 34

Supplementary Note 7: Properties of $Lu_3Al_5O_{12}$ and Y_2O_2S 36

Supplementary Note 8: Influence of light exposure on ML 54

Supplementary Note 9: Differences of scratching and stretching 56

Supplementary Note 10: Analysis of the contact interface 57

Supplementary Note 11: ML performance during a single stretching 62

Supplementary Note 12: Relatively electronegativity of the phosphors and PDMS ... 65

Supplementary Note 13: The role of friction on the triboelectrification 67

Supplementary Note 14: Humidity and other factors affecting ML intensity 70

Supplementary Note 15: More explanation on the scratching-induced separation 74

Supplementary Note 16: First-principles calculation of contact-separation 77

Supplementary Note 17: Additional first-principles calculations 87

Supplementary Note 18: A brief introduction to the AFM and EFM techniques 121

Supplementary References 122

Supplementary Note 1: Why do we focus on centrosymmetric phosphors?

Generally, the piezoelectricity manifests only in phosphors with asymmetrical piezoelectric structures, whereas the contact electrification occurs in almost all phosphors regardless of structural symmetry. However, because piezoelectricity in piezoelectric structures is significantly stronger than contact electrification, it is very difficult to observe the contact electrification effect in piezoelectric materials. Consequently, all previously reported MLs in piezoelectric materials were attributed to the piezoelectricity rather than contact electrification, as exhibited in Supplementary Table 1-3. Even the few centrosymmetric phosphors without piezoelectric effect emit ML under stress, and their MLs are still attributed to the local piezoelectricity due to doping (marked "local pieze." in Supplementary Table 1-3). In general, it is easier to observe the contact electrification effect at the interface of two insulators. The most well-known example of contact electrification appearing in textbooks is the rubbing of an animal's fur against a plastic rod. Since both the plastic rod and animal's fur are insulators, we can observe some typical static electricity phenomena due to contact electrification, such as static sparks and beeping noise. Correspondingly, centrosymmetric phosphors with large dielectric constant exhibit physical properties similar to insulators, and therefore we can observe stronger contact electrification effect in the centrosymmetric phosphors. For example, our group has reported a series of ML elastomers based on PDMS and centrosymmetric phosphors such as $Sr_3Al_2O_6:Eu^{3+}$, $Y_3Al_5O_{12}:Ce^{3+}$, $Lu_3Al_5O_{12}:Ce^{3+}$ and $Gd_5Ga_3O_{12}:RE^{3+}$ [1–4], which show much stronger ML than $ZnS:Cu,Mn^{2+}$/PDMS elastomer. Accordingly, it is worthwhile to research the ML properties of the centrosymmetric phosphors.

Supplementary Table 1. Crystal structure, piezoelectricity (piezoe.) -induced ML, contact-electrification-induced ML for reported ML materials (Triclinic, Monoclinic, Orthorhombic).

Crystal system	Point group	symmetry order	Piezoe. structure	Material hosts	Piezoe.-induced ML	Contact-electrification-induced ML
Triclinic	1	1	√	$CaAl_2Si_2O_8$	√	
				$SrAl_2Si_2O_8$		
				$SrSi_2O_2N_2$		
	$\bar{1}$	2	×		×	
Monoclinic	*m*	2	√		√	
	2	2	√	$SrAl_2O_4$	√	
				$SrMg_2(PO_4)_2$		
				$Ca_2Nb_2O_7$		
				$Sr_2Nb_2O_7$		
	2/*m*	4	×		×	
Orthorhombic	*mm2*	4	√	$Ca_3Ti_2O_7$	√	
				$BaSi_2O_2N_2$		
				$SrZn_2S_2O$		
				$Sr_3Sn_2O_7$		
	222	4	√	$CaZr(PO4)_2$	√	
	mmm	8	×	$NaNbO_3$	√ (local piezoe.)	
				BaZnOS		
				$CaNb_2O_6$		

Supplementary Table 2. Crystal structure, piezoelectricity (piezoe.) -induced ML, contact-electrification-induced ML for reported ML materials (Trigonal, Tetragonal).

Crystal system	Point group	symmetry order	Piezoe. structure	Material hosts	Piezoe.-induced ML	Contact-electrification-induced ML
Trigonal	3	3	√	$Zn_2(Ge,Si)O_4$	√	
	$\bar{3}$	6	×		×	
	3*m*	6	√	$LiNbO_3$	√	
	32	6	√		√	
	$\bar{3}$m	12	×		×	
Tetragonal	4	4	√		√	
	$\bar{4}$	4	√		√	
	4/*m*	8	×		×	
	4*mm*	8	√	$(Ba,Ca)TiO_3$	√	
	$\bar{4}$2m	8	√	$Ca_2Al_2SiO_7$<br>$CaYAl_3O_7$<br>$Ca_2MgSi_2O_7$<br>$Sr_2MgSi_2O_7$	√	
	422	8	√		√	
	4/*mmm*	16	×	$Ca_3Nb_2O_8$	√ (local piezoe.)	

Supplementary Table 3. Crystal structure, piezoelectricity (piezoe.) -induced ML, contact-electrification-induced ML for reported ML materials (Hexagonal, Cubic).

Crystal system	Point group	symmetry order	Piezoe. structure	Material hosts	Piezoe.-induced ML	Contact-electrification-induced ML
Hexagonal	6	6	√		√	
	$\bar{6}$	6	√		√	
	6/m	12	×		×	
	$\bar{6}m2$	12	√		√	
		12	√	ZnS	√	
		12	√		√	
		24	×		×	
Cubic	23	12	√		√	
	*m*3	24	×		×	
	$\bar{4}3m$	24	√		√	
	432	24	×		×	
	*m*3*m*	48	×	$ZnAl_2O_4$	×	√ (tribe.)
				$MgGa_2O_4$		√ (tribe.)
				$ZnGa_2O_4$		√ (tribe.)
				$Y_3Al_5O_8$		√
				$Lu_3Al_5O_8$		
				$Gd_5Ga_3O_{12}$		√

Supplementary Note 2: Microstructure and elemental composition of CaF_2

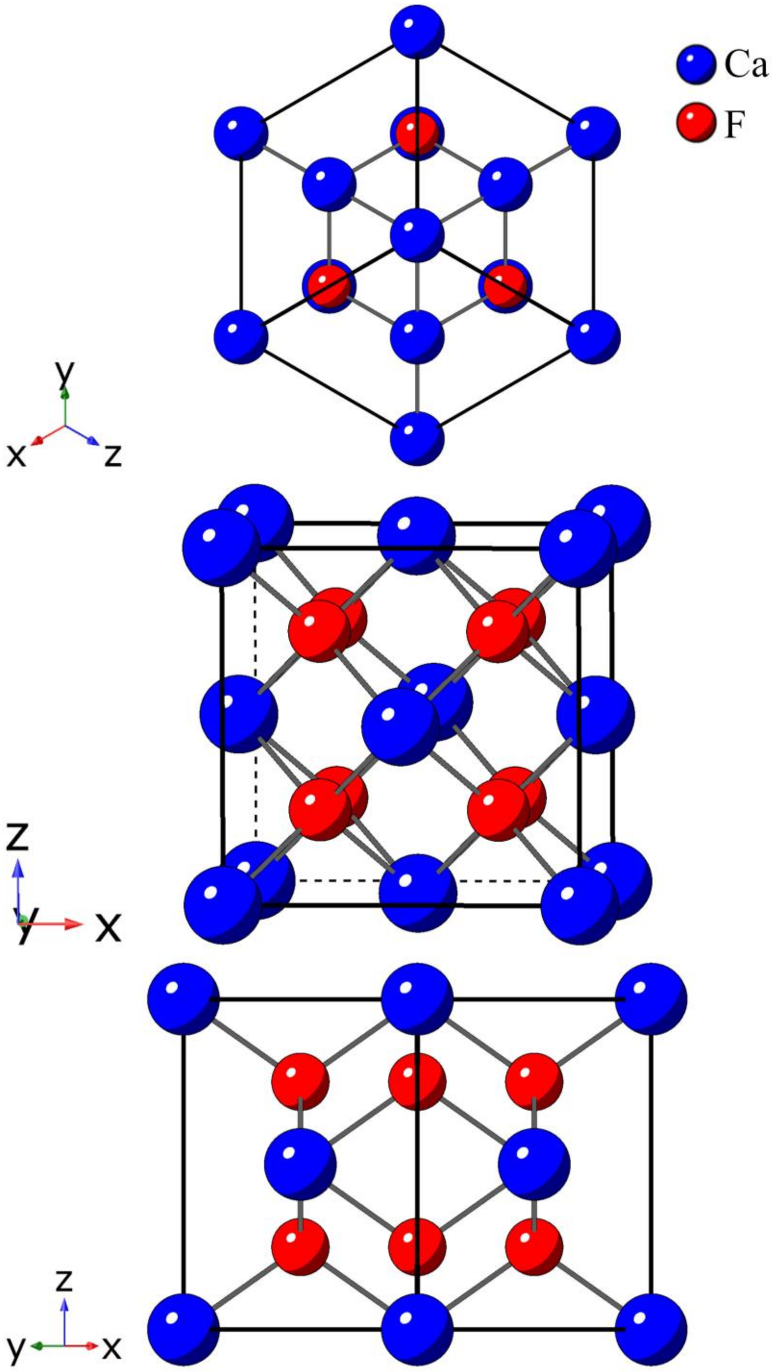

Supplementary Figure 1. Crystal structure of CaF_2

Supplementary Table 4. Crystallographic data of CaF_2

Formula	CaF_2
Crystal system	Cubic
Space group	Fm-3m (225)
Lattice parameters	
a = b = c	5.458 Å
$\alpha = \beta = \gamma$	90°
Cell volume	162.634 Å^3
T	289 K
Diffractometer	Rigaku D/Max-2400
Radiation/Å	Cu-Ka (λ= 1.5405)
Absorption correction	multi-scan
2θ range	8°-80°
Z	4
Calculated Density	3.189 g/cm^3
R-factors	
R_{wp}	0.0943
R_p	0.0672
χ^2	1.769

Supplementary Table 5. Refined coordinates of all atoms, bond lengths of X-F and the unit cell parameters of the CaF_2 as determined by the GSAS program

Space group	Fm-3m (225) - Cubic				
Cell	a = b = c = 5.458 Å				
Ion coordinates	x	y	z	Occupancy	Uiso
Ca	0.0000(0)	0.0000(0)	0.0000(0)	1.000(0)	0.0100(0)
F	0.2500(0)	0.2500(0)	0.2500(0)	0.2500(0)	0.0100(0)

The bond lengths of X-F	
Vector	Length
Ca_F	2.36358(4)

Supplementary Table 6. The bandgap of CaF_2 calculated by Vienna Ab-initio Simulation Package (VASP)

Band Character	Indirect
Band Gap (eV)	7.3822
Eigenvalue of VBM (eV)	-0.4827
Eigenvalue of CBM (eV)	6.8996
Fermi Energy (eV)	0.4076
HOMO & LUMO Bands	12 13
Location of VBM	0.500000 0.000000 0.500000
Location of CBM	0.000000 0.000000 0.000000

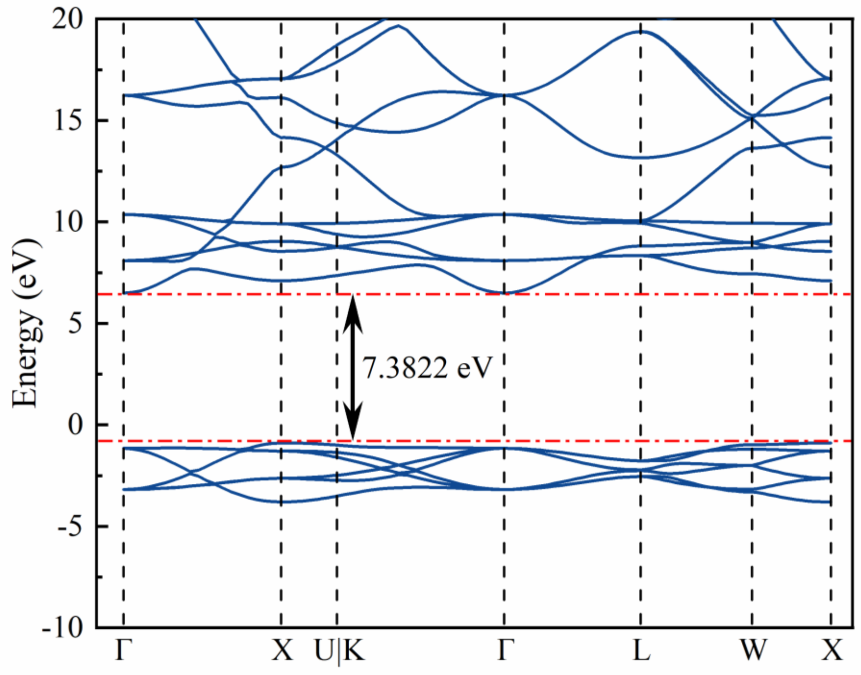

Supplementary Figure 2. Calculated energy band structure of CaF_2

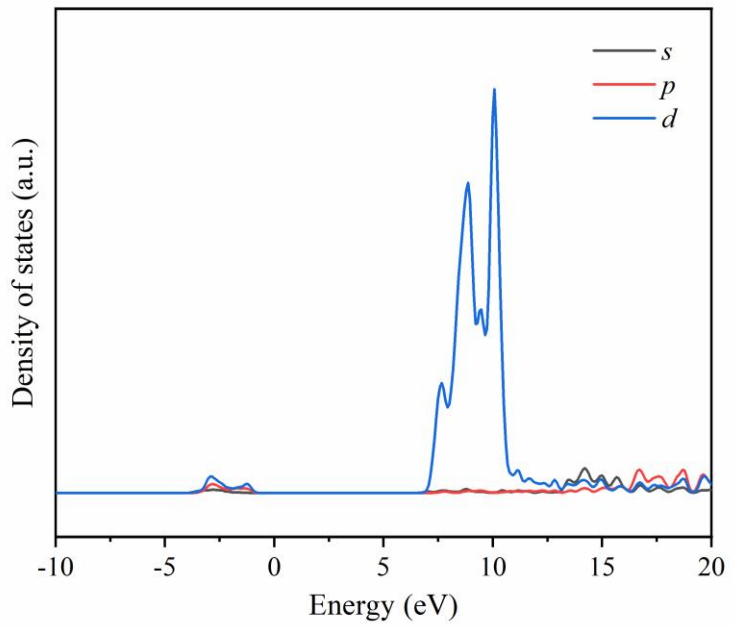

Supplementary Figure 3. Partial density of states (Ca atoms) of CaF_2

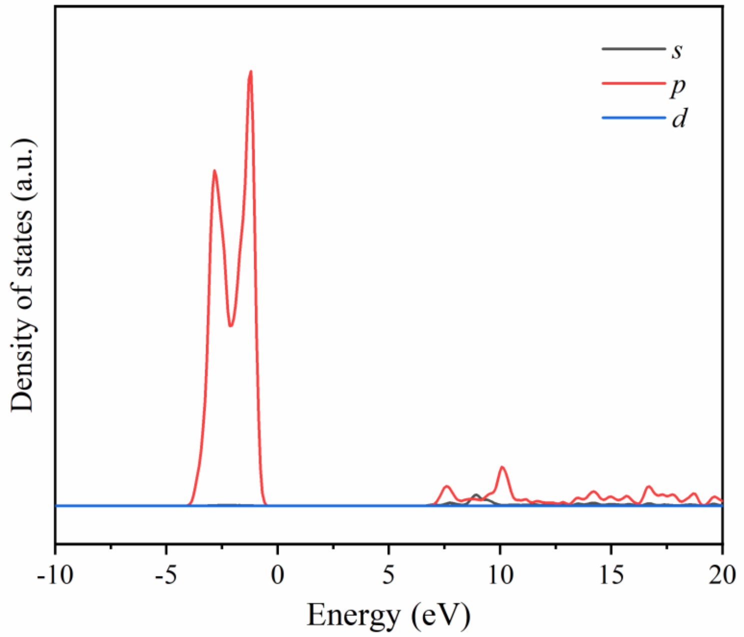

Supplementary Figure 4. Partial density of states (F atoms) of CaF_2

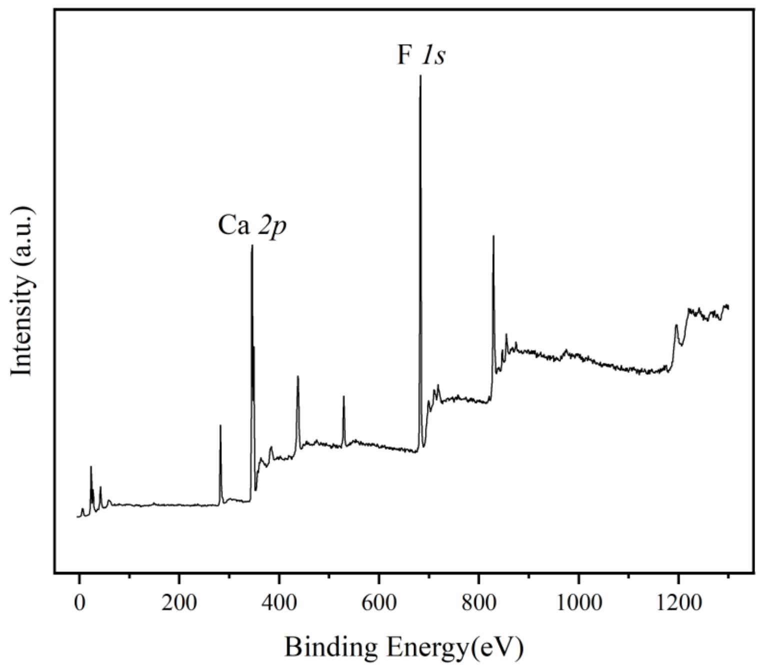

Supplementary Figure 5. X-ray photoelectron spectroscopy(XPS) spectrum of CaF_2

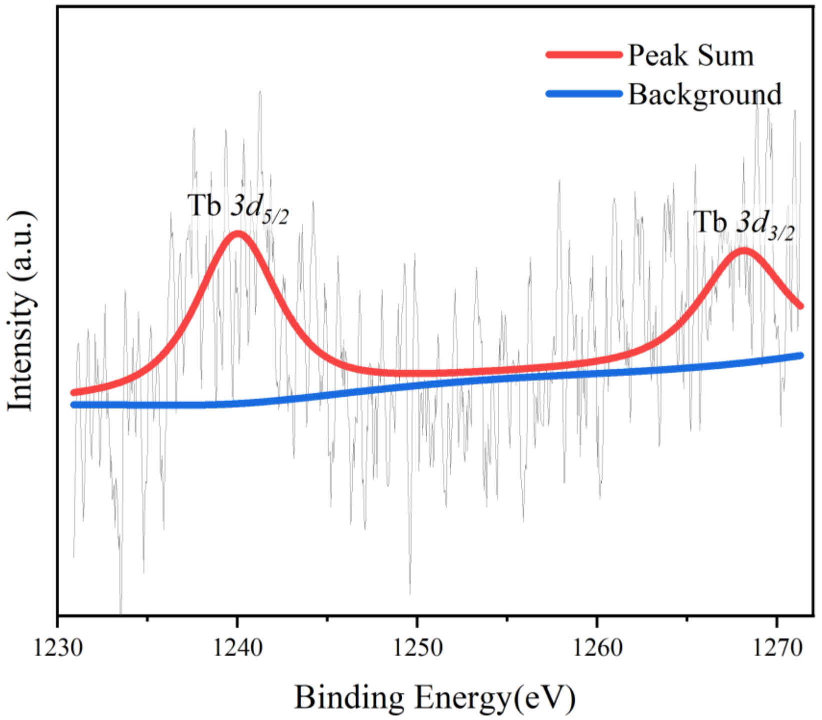

Supplementary Figure 6. High-resolution XPS spectra of Tb *3d*

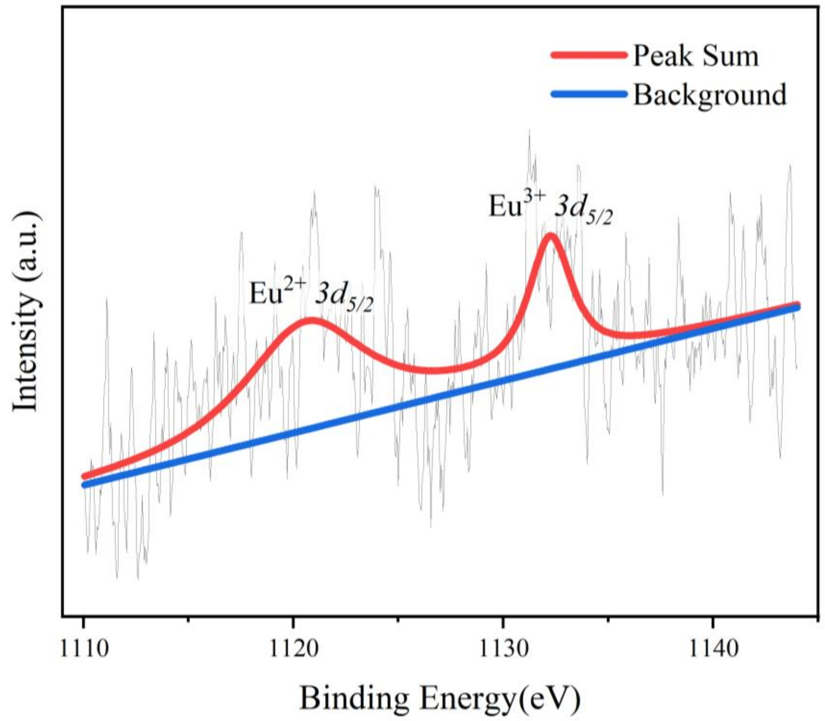

Supplementary Figure 7. High-resolution XPS spectra of Eu *3d*

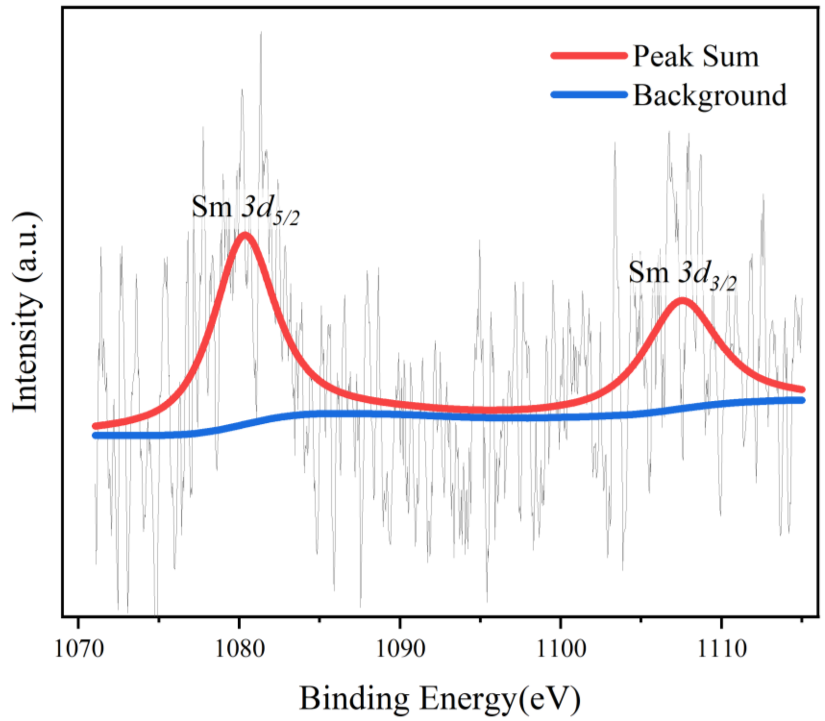

Supplementary Figure 8. High-resolution XPS spectra of Sm *3d*

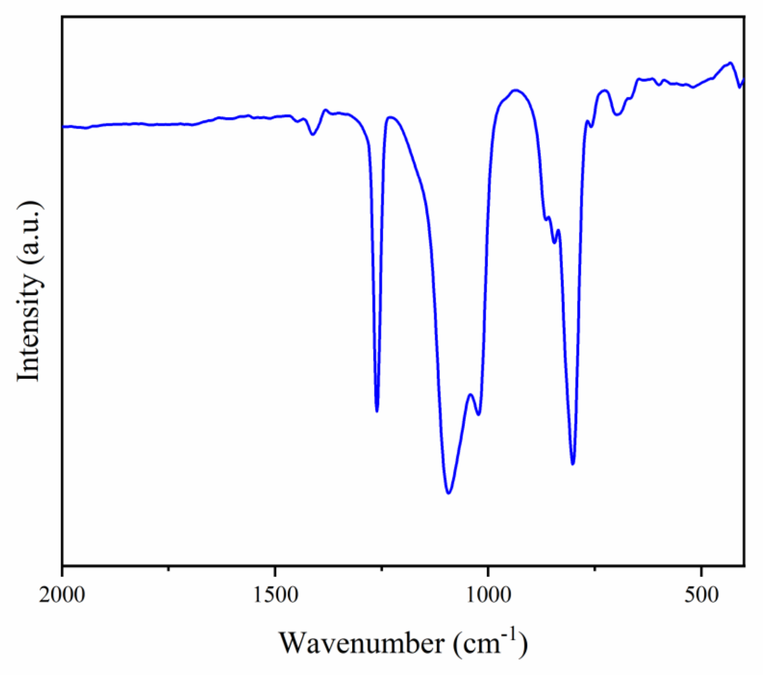

Supplementary Figure 9. Infrared absorption spectrum of PDMS

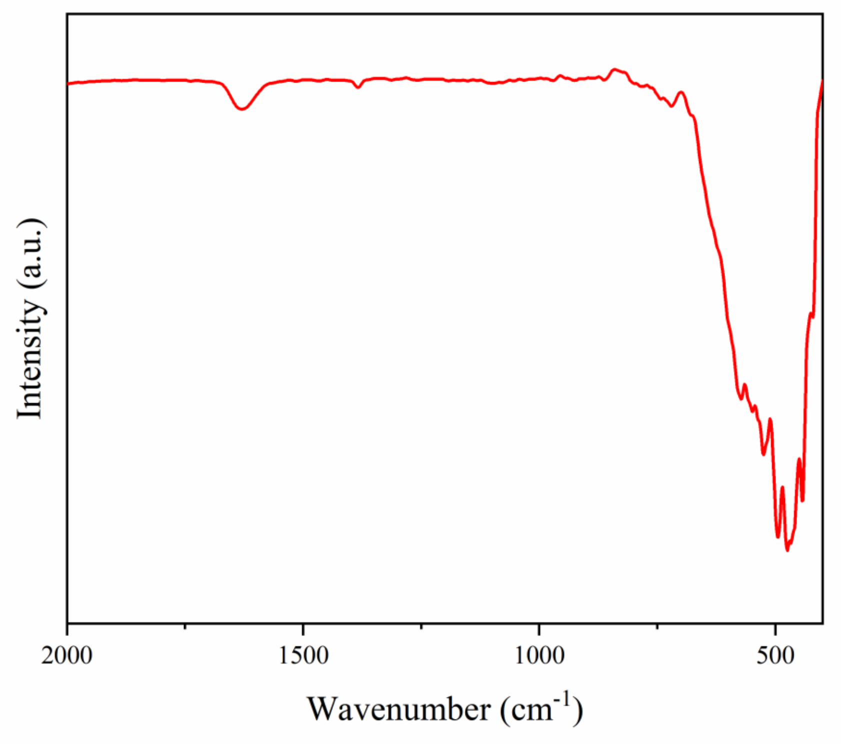

Supplementary Figure 10. Infrared absorption spectrum of $CaF_2:Tb^{3+}$

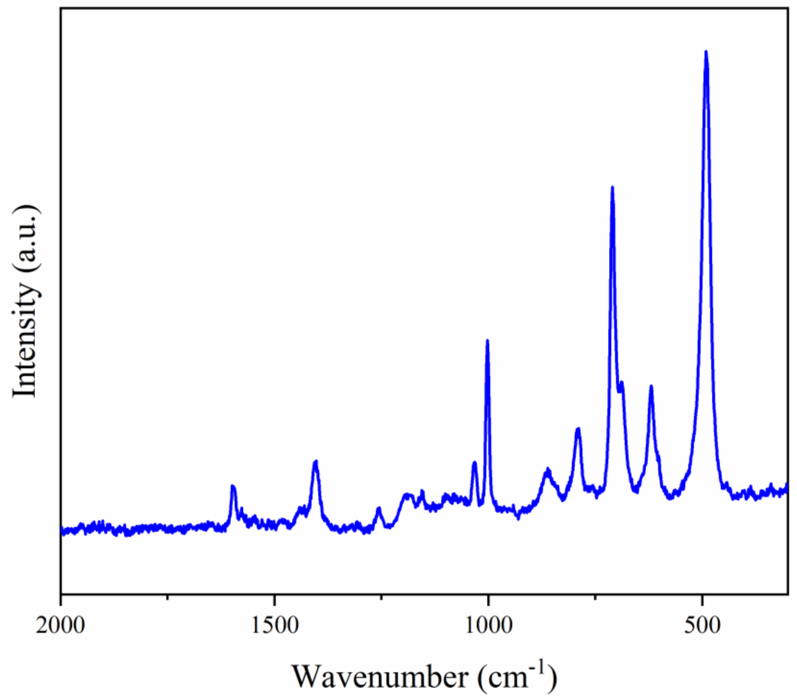

Supplementary Figure 11. Raman spectroscopy of PDMS

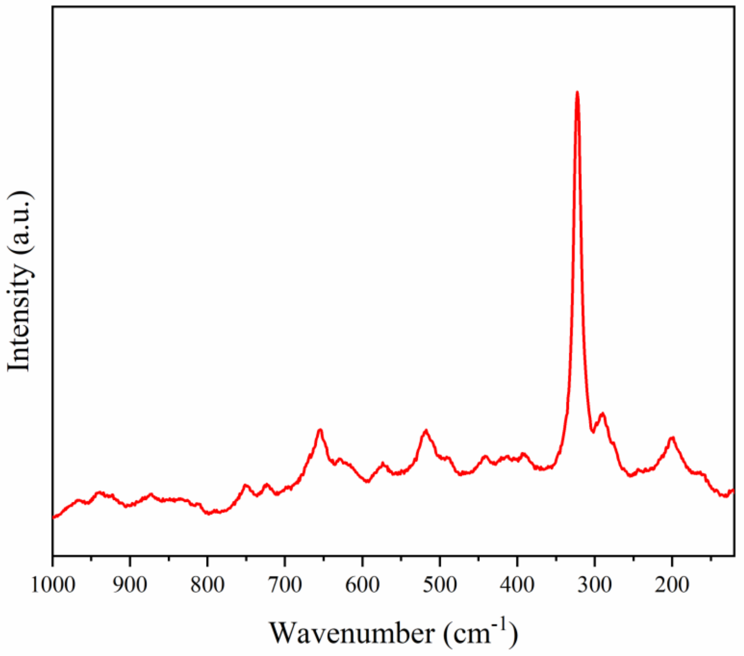

Supplementary Figure 12. Raman spectroscopy of $CaF_2:Tb^{3+}$

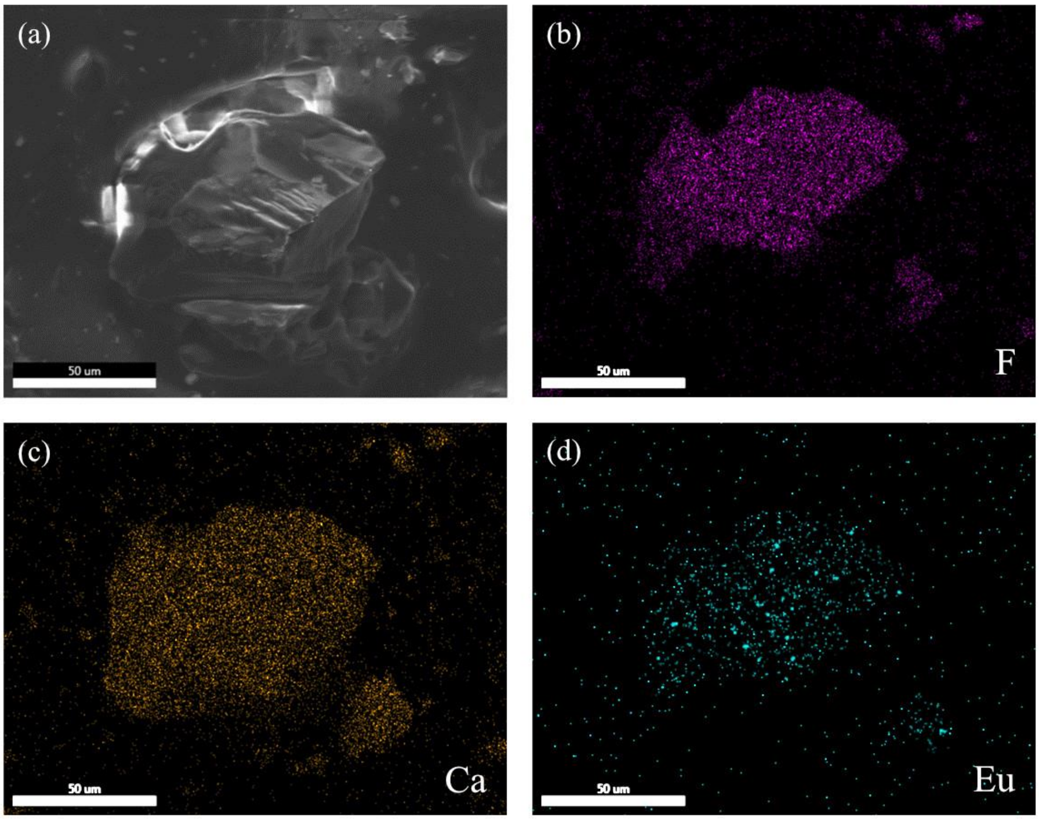

Supplementary Figure 13. Elemental distribution map of CaF_2:Eu^{2+}

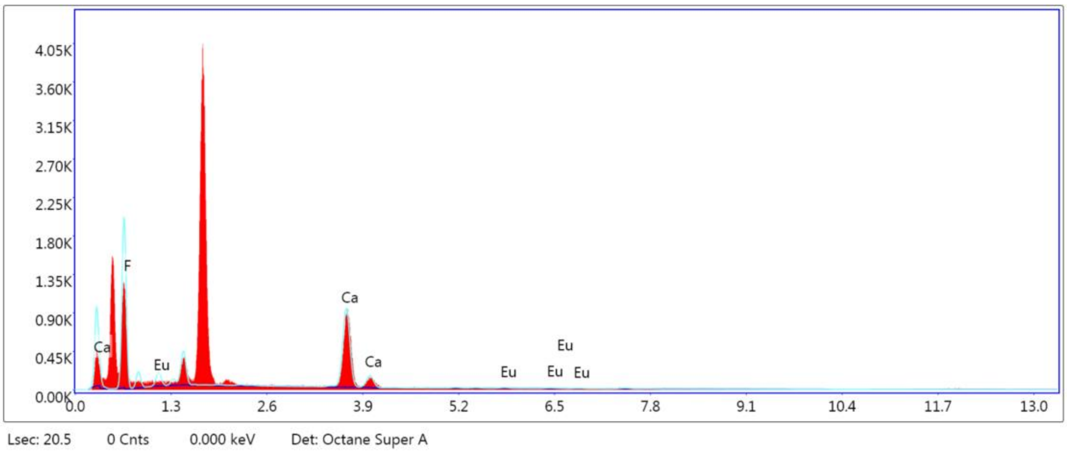

Supplementary Figure 14. Energy dispersive spectra (EDS) of $CaF_2:Eu^{2+}$

Supplementary Table 7. Elements analysis of $CaF_2:Eu^{2+}$

Element	Weight %	Atomic %	Error %
**F**	64.94	80.04	8.68
**Ca**	33.85	19.77	2.44
**Eu**	1.21	0.19	57.30

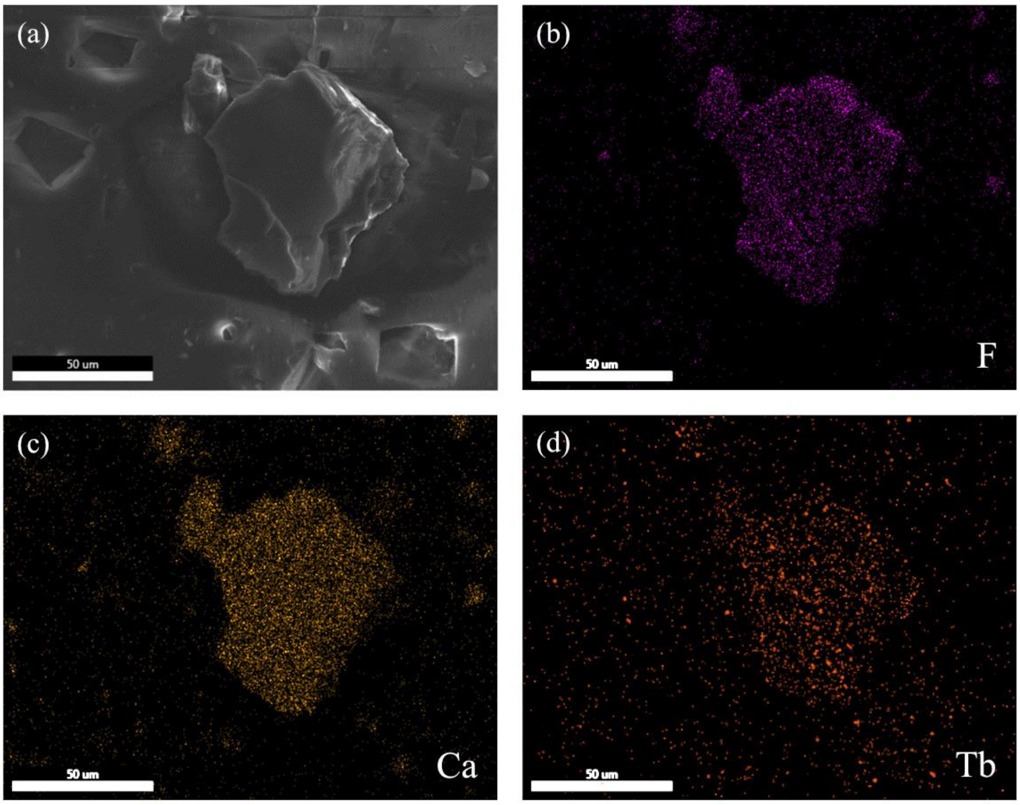

Supplementary Figure 15. Elemental distribution map of CaF_2:Tb^{3+}

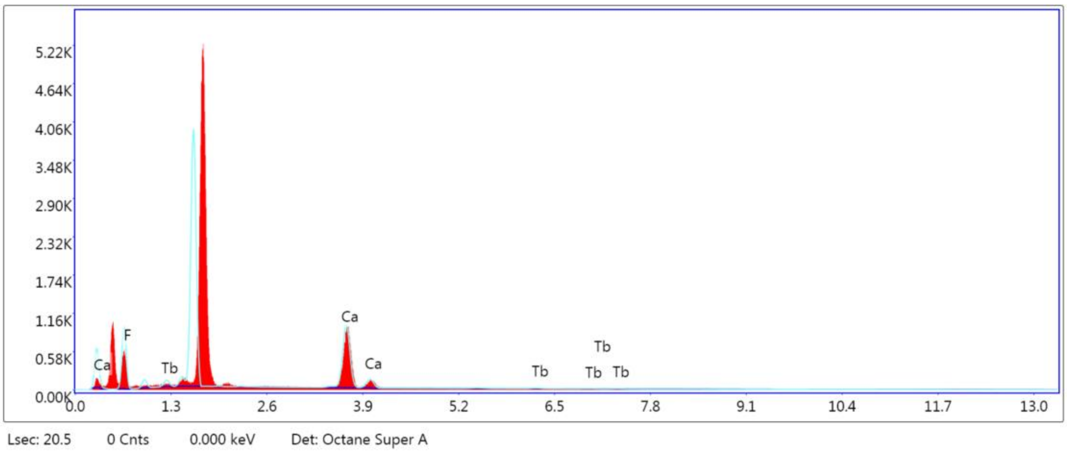

Supplementary Figure 16. Energy dispersive spectra (EDS) of CaF_2:Tb^{3+}

Supplementary Table 8. Elements analysis of CaF_2:Tb^{3+}

Element	Weight %	Atomic %	Error %
**F**	51.83	70.21	9.78
**Ca**	45.79	29.41	2.47
**Tb**	2.38	0.39	56.76

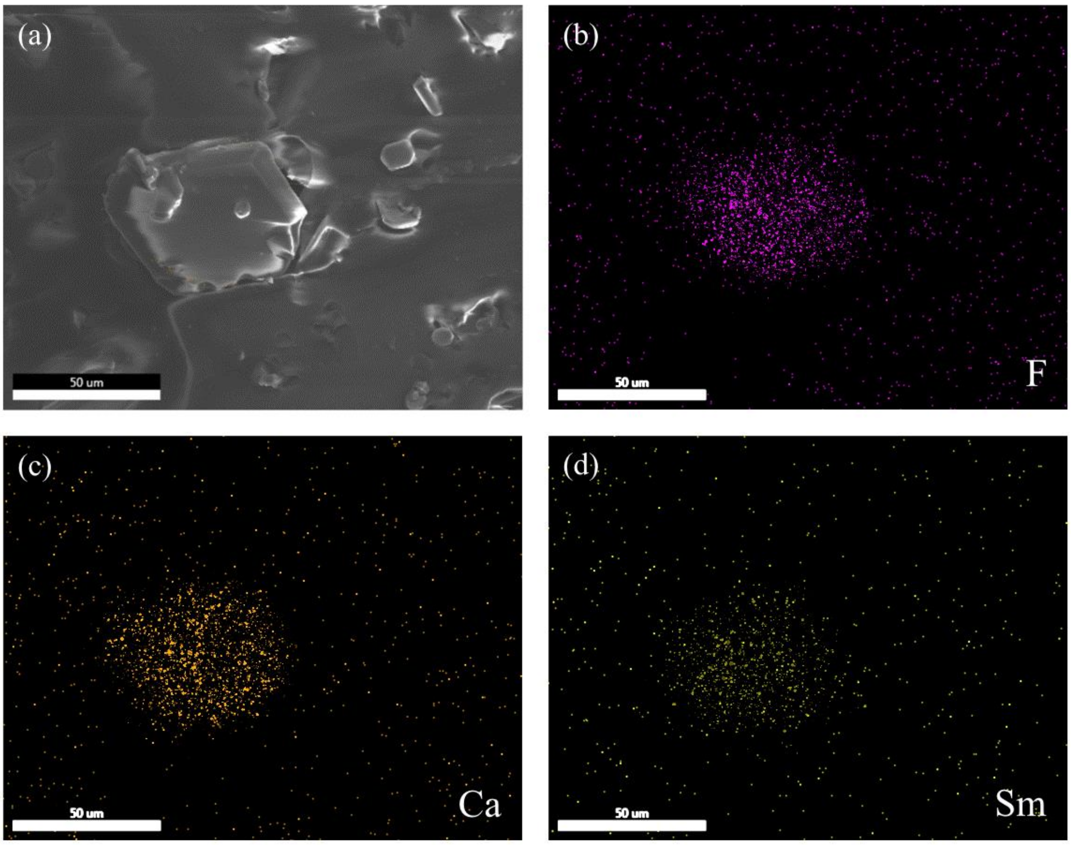

Supplementary Figure 17. Elemental distribution map of $CaF_2:Sm^{3+}$

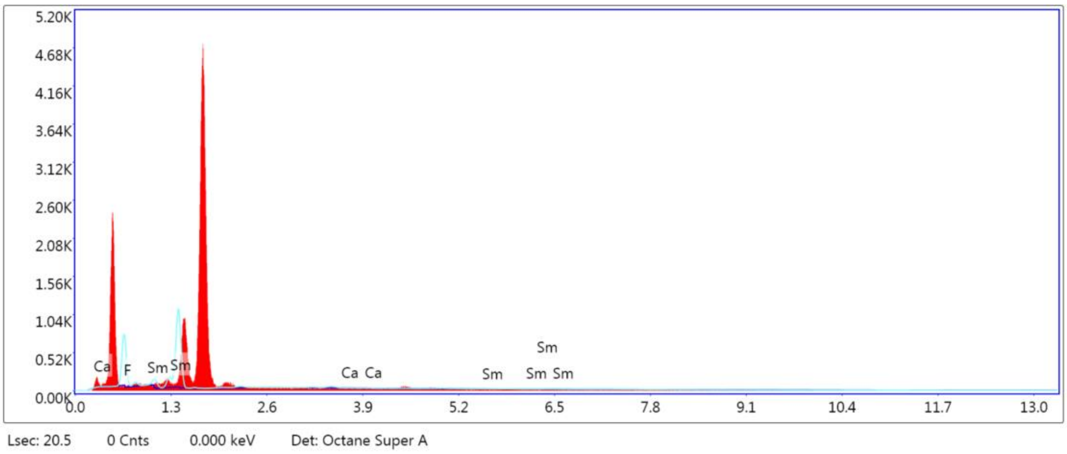

Supplementary Figure 18. Energy dispersive spectra (EDS) of $CaF_2:Sm^{3+}$

Supplementary Table 9. Elements analysis of $CaF_2:Sm^{3+}$

Element	Weight %	Atomic %	Error %
**F**	89.15	98.17	4.50
**Ca**	0.84	0.44	90.83
**Sm**	10.01	1.39	42.73

Supplementary Note 3: Charge compensation mechanism

For the CaF_2:Tb^{3+} phosphors, the Tb^{3+} ion is trivalent, but there is only one kind of bivalent calcium ion in the CaF_2 crystal, so if the Tb^{3+} replaces the Ca^{2+} site, it will inevitably induce a significant charge imbalance. Generally, there are two possible methods to keep the charge balance when the Tb^{3+} ions are doped in the CaF_2 crystal. First, two Tb^{3+} ions replace two Ca^{2+} cations and it creates one Ca^{2+} vacancy. Second, two Ca^{2+} cations are substituted for one Tb^{3+} ion and it generates one F^- vacancy and one Ca^{2+} vacancy.

To figure out the charge compensation mechanism of the non-equivalent doping of Tb^{3+} in CaF_2 crystal, three specific kinds of defects, which are $[Tb_{Ca}^{\bullet}]$, Ca^{2+} vacancy $[V_{Ca}'']$ and F^- vacancy $[V_F^{\bullet}]$ were built up in CaF_2 crystal as shown in the following Supplementary Figure 19 Then, the DFT calculations based on a VASP package are conducted. Finally, the cohesive energies for the different defects including the $[Tb_{Ca}^{\bullet}]$-$[V_F^{\bullet}]$-$[V_{Ca}'']$, 2$[Tb_{Ca}^{\bullet}]$-$[V_{Ca}'']$ and $[Tb_{Ca}^{\bullet}]$ can be evaluated and the DFT calculation results are presented in the Supplementary Table 10.

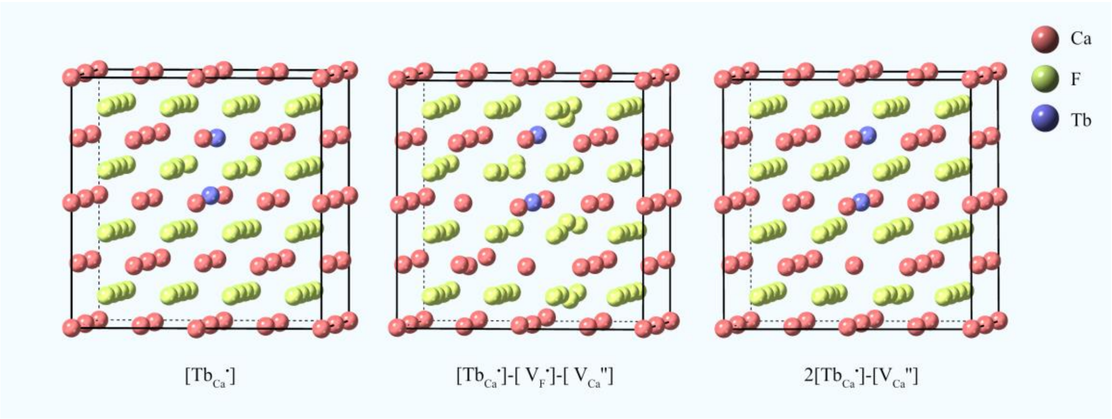

Supplementary Figure 19. Three specific kinds of defects including $[Tb_{Ca}^{\bullet}]$, Ca vacancy $[V_{Ca}'']$ and F vacancy $[V_F^{\bullet}]$ in CaF_2 crystal for DFT calculation, and the Tb^{3+} substitution ratio is unified to 2/32

Supplementary Table 10. DFT calculated cohesive energies (E_c) for the different defects including the $[Tb_{Ca}^{\bullet}]$-$[V_F^{\bullet}]$-$[V_{Ca}'']$, 2$[Tb_{Ca}^{\bullet}]$-$[V_{Ca}'']$ and $[Tb_{Ca}^{\bullet}]$ in the CaF_2 crystal

Defects	Number of atoms	Cell E_c (eV)	Atomic E_a (eV)
$[Tb_{Ca}^{\bullet}]$-$[V_F^{\bullet}]$-$[V_{Ca}'']$	92	-536.125	-5.796
2$[Tb_{Ca}^{\bullet}]$-$[V_{Ca}'']$	95	-556.630	-5.828
$[Tb_{Ca}^{\bullet}]$	96	-557.806	-5.779

Supplementary Table 10 presents the cell cohesive energies (E_c) for the different defects in the CaF_2 crystal. Since there are different number of atoms in these cells, the cell Ec should be subtracted from the free state energy of each atom and divided by the number of atoms to obtain the atomic Ea for different defects. The results indicate that the atomic E_a (-5.828 eV) for the 2$[Tb_{Ca}^{\bullet}]$-$[V_{Ca}'']$ defect cluster is the smallest as shown in Supplementary Table 10. It means that two Tb^{3+} dopants would replace two Ca^{2+} cations in the CaF_2 crystal, resulting in one Ca^{2+} vacancy $[V_{Ca}'']$ to keep the charge balance in the CaF_2 crystal.

However, the above discussion based on the DFT calculations is still a theoretical analysis, and at present there is still a lack of sound experimental evidence for which defect belongs to. Here, we can only make some theoretical discussions to help the readers understand the charge compensation mechanism of the non-equivalent doping of Tb^{3+} in the CaF_2 crystal.

Supplementary Note 4: Spectra of CaF_2: Tb^{3+}/Eu^{2+}/Sm^{3+}

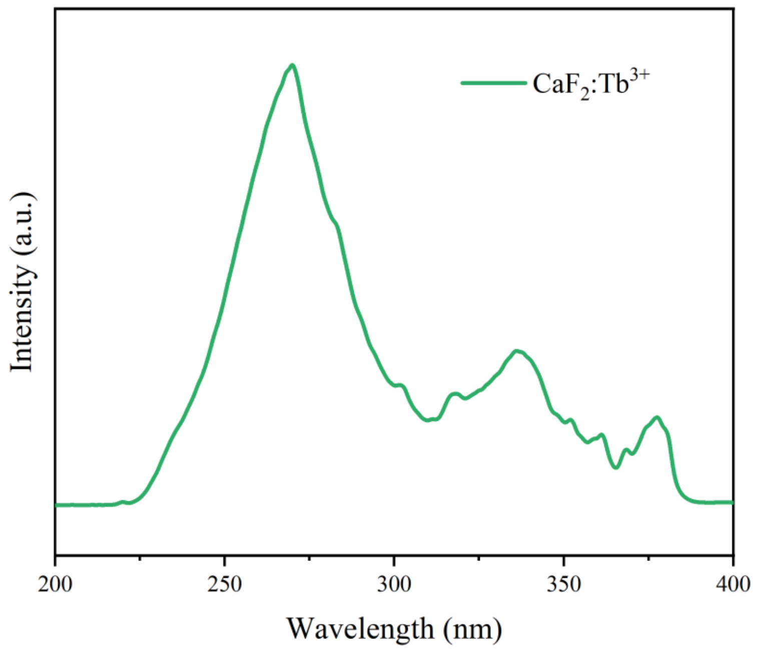

Supplementary Figure 20. Excitation spectrum of $CaF_2:Tb^{3+}$

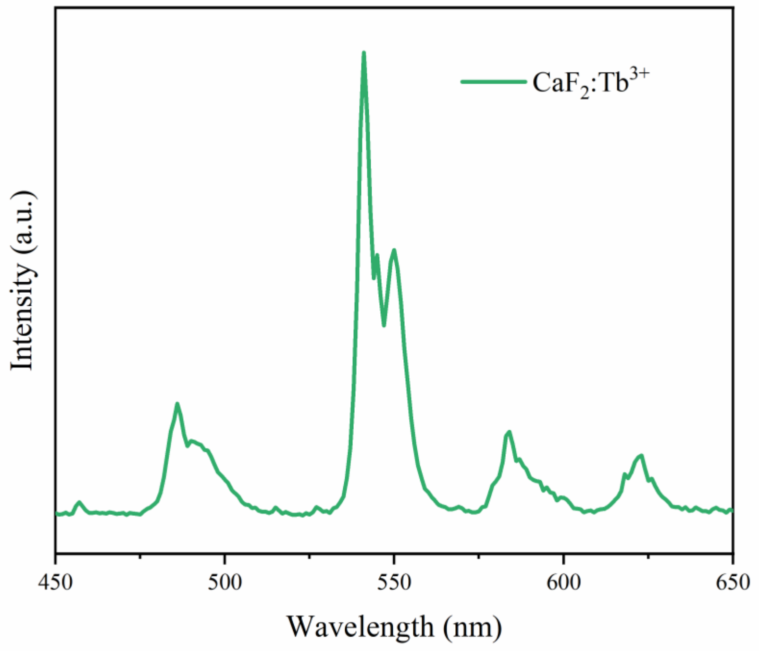

Supplementary Figure 21. Emission spectrum of $CaF_2:Tb^{3+}$

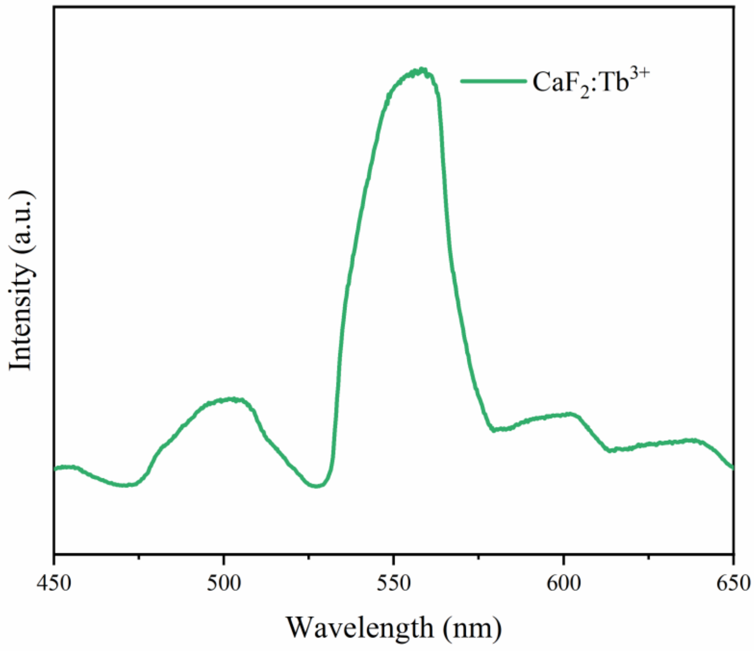

Supplementary Figure 22. ML spectrum of $CaF_2:Tb^{3+}$

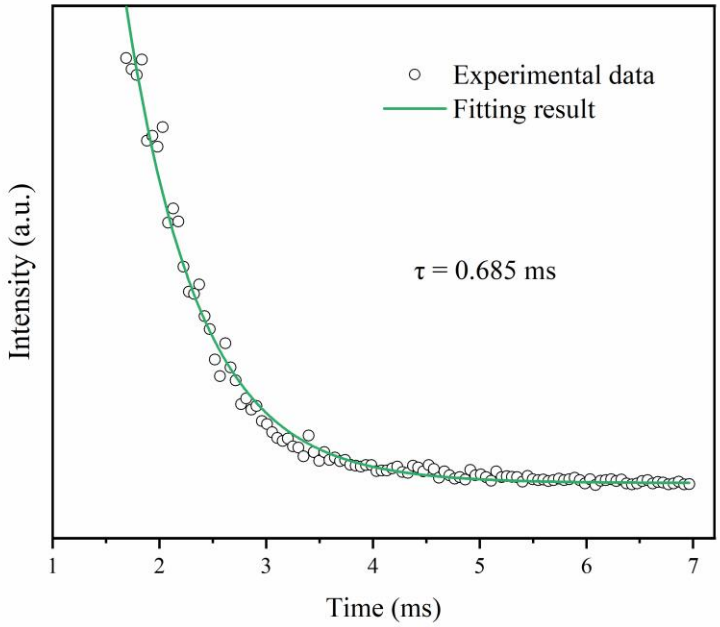

Supplementary Figure 23. Luminescence decay curve of $CaF_2:Tb^{3+}$

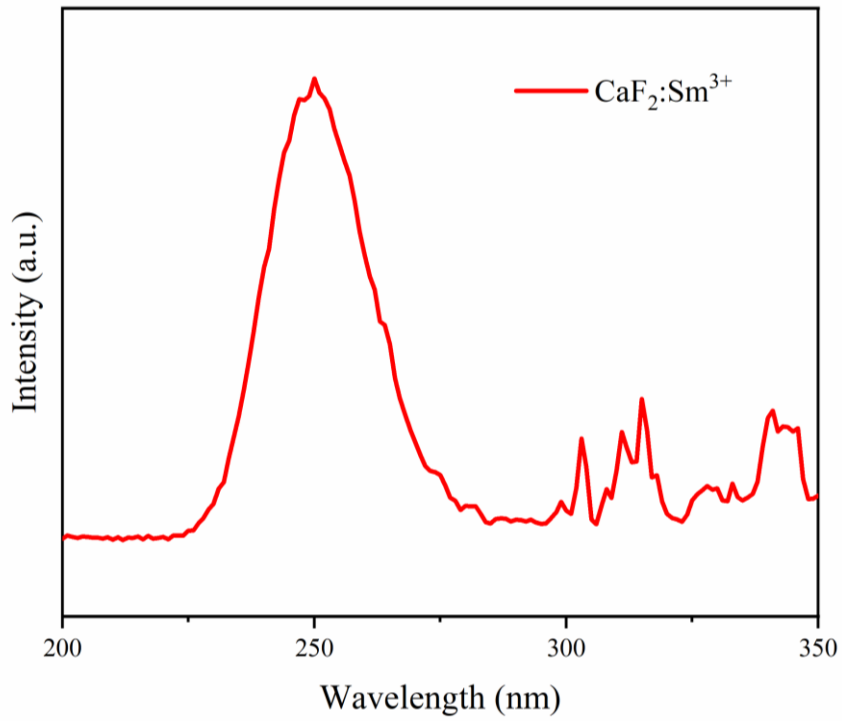

Supplementary Figure 24. Excitation spectrum of $CaF_2:Sm^{3+}$

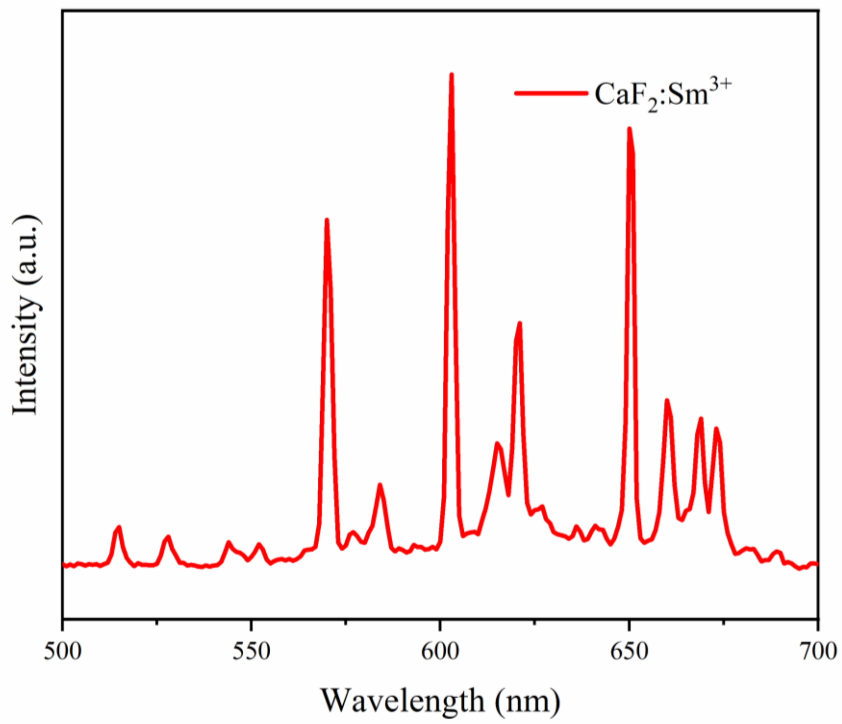

Supplementary Figure 25. Emission spectrum of $CaF_2:Sm^{3+}$

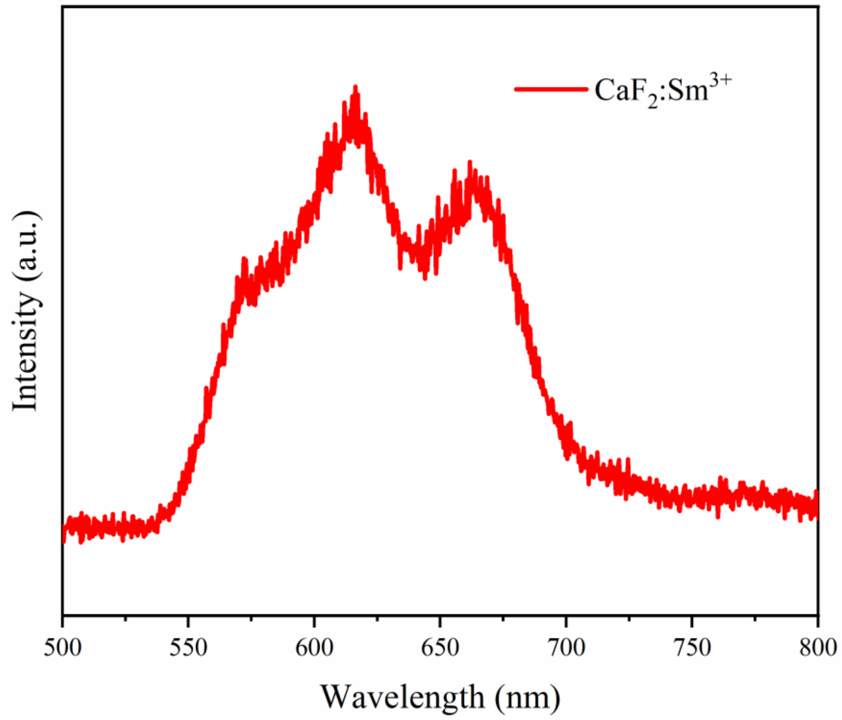

Supplementary Figure 26. ML spectrum of $CaF_2:Sm^{3+}$

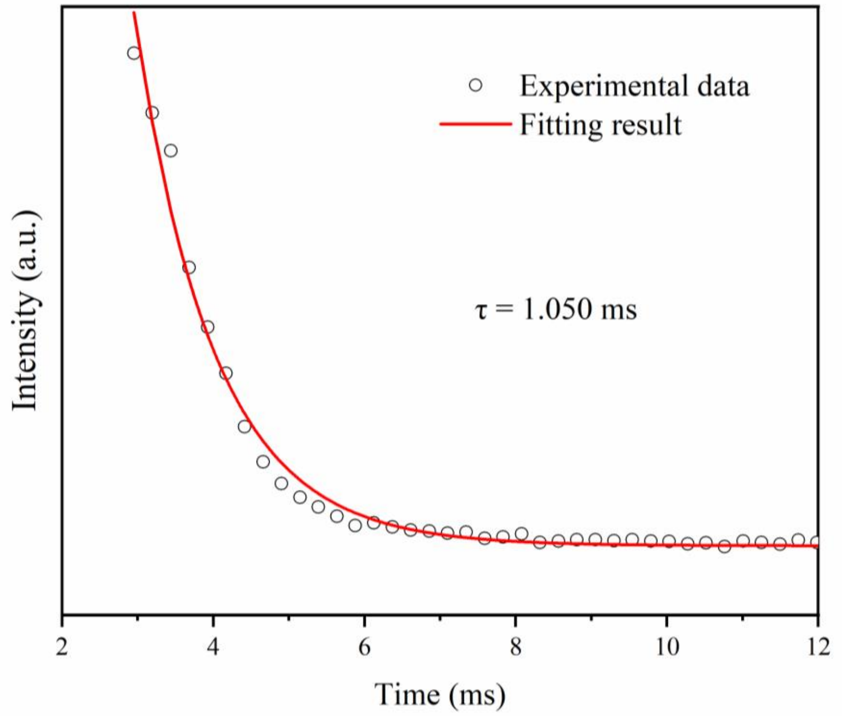

Supplementary Figure 27. Luminescence decay curve of $CaF_2:Sm^{3+}$

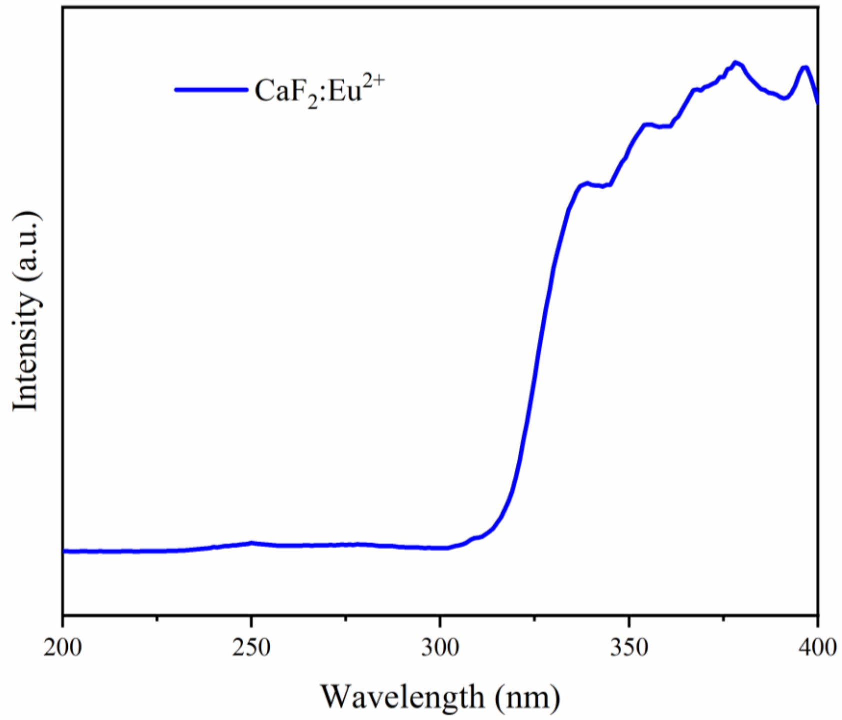

Supplementary Figure 28. Excitation spectrum of $CaF_2:Eu^{2+}$

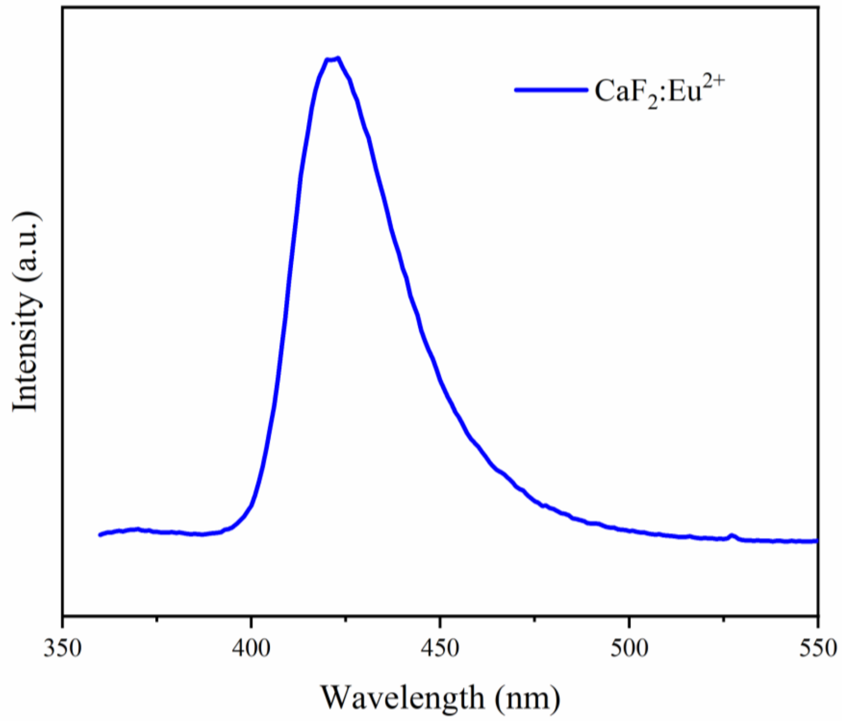

Supplementary Figure 29. Emission spectrum of $CaF_2:Eu^{2+}$

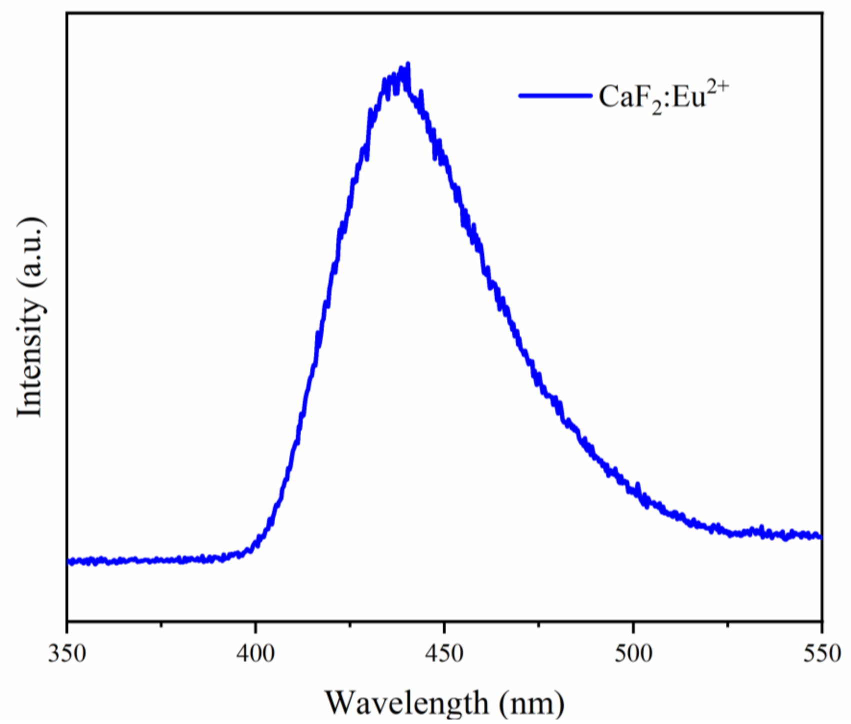

Supplementary Figure 30. ML spectrum of CaF_2:Eu^{2+}

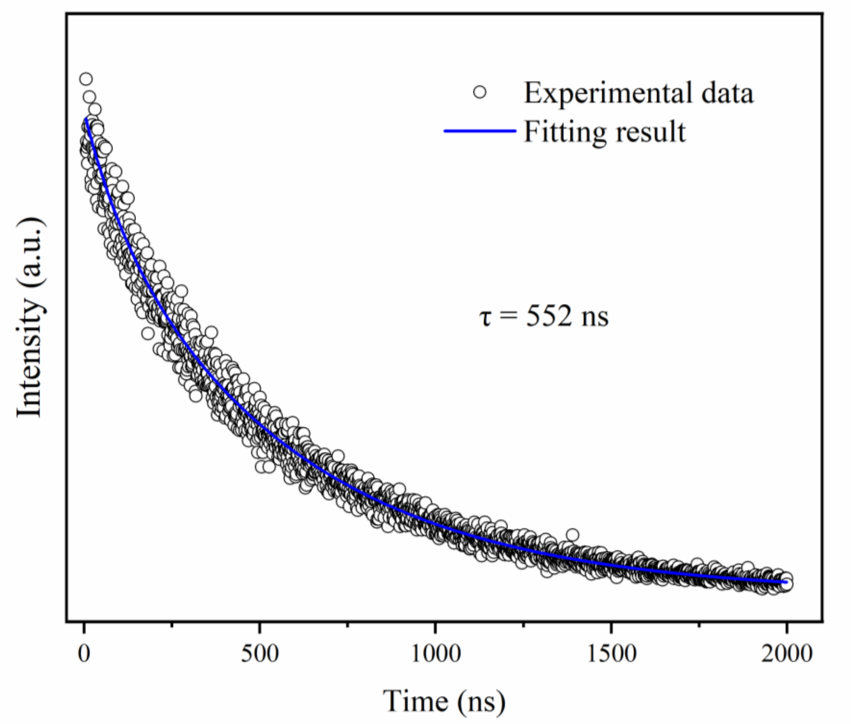

Supplementary Figure 31. Luminescence decay curve of CaF_2:Eu^{2+}

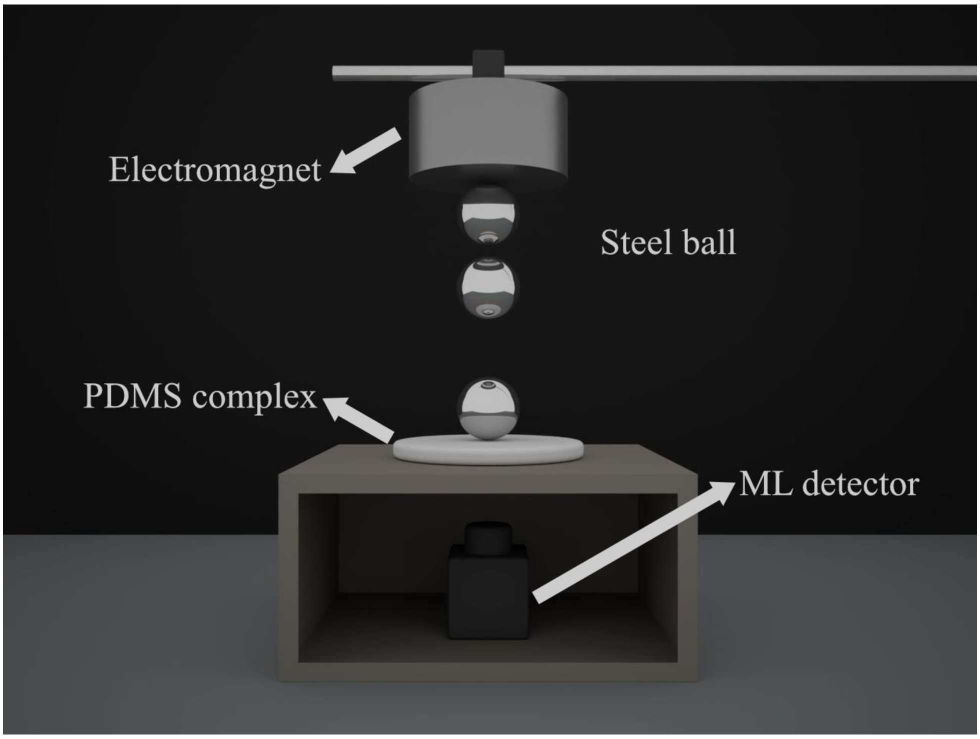

Supplementary Figure 32. Schematic diagram of a falling ball experimental setup

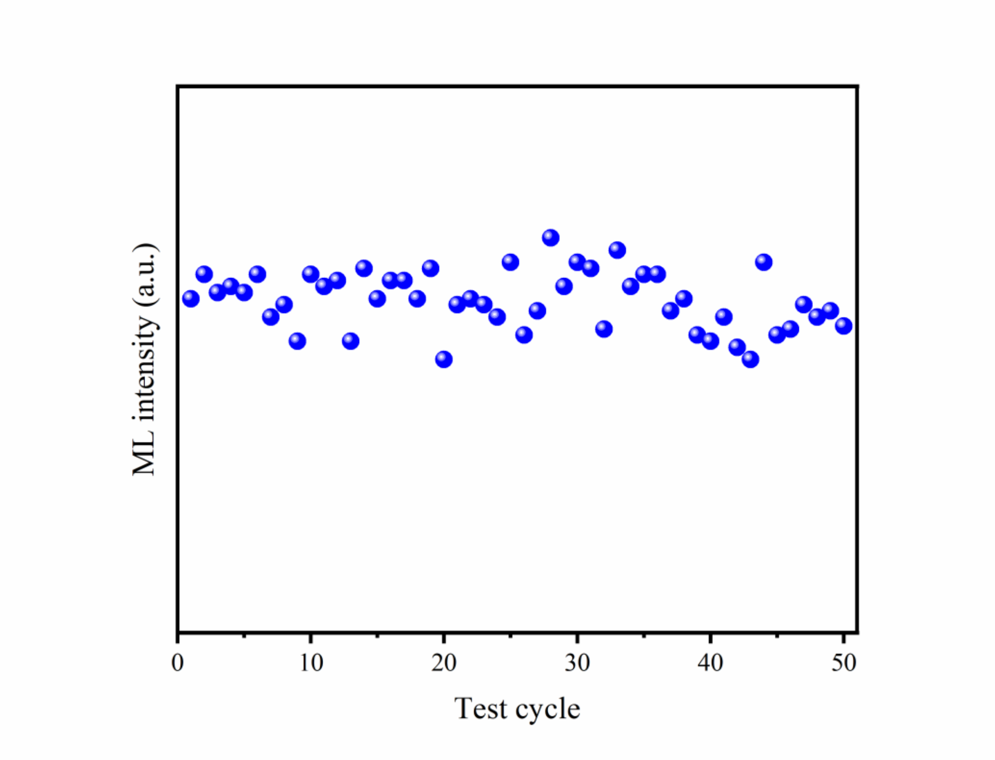

Supplementary Figure 33. ML intensity of $CaF_2:Eu^{2+}$/PDMS in 50 repeated tests

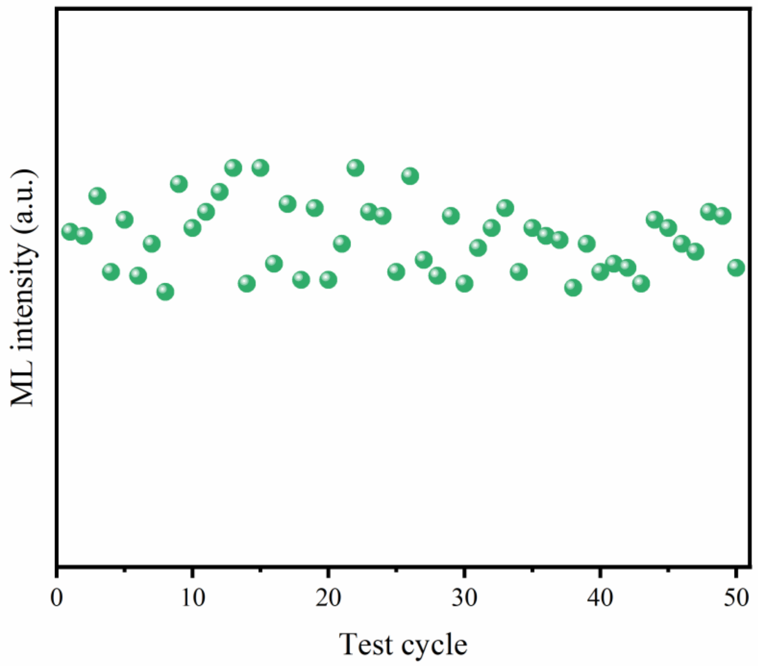

Supplementary Figure 34. ML intensity of $CaF_2:Tb^{3+}$/PDMS in 50 repeated tests

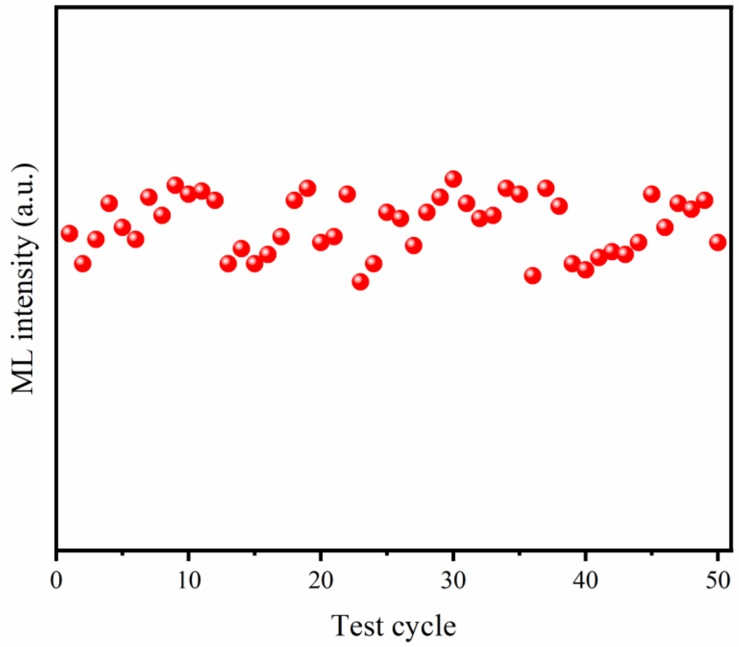

Supplementary Figure 35. ML intensity of $CaF_2:Sm^{3+}$/PDMS in 50 repeated tests

Supplementary Note 5: Two-dimensional stretching machine

To investigate the ML self-recover ability of the composite under two-dimensional alternating stretching, we developed a dedicated stretching machine featuring a mechanical structure depicted in the Supplementary Figures 36-41. To ensure utmost precision and force consistency during stretching, we equipped the machine with two high-precision stepping motors and a 1:19 planetary reducer. The control system is based on an MKS GEN-L V2.1 motherboard running on open source Marlin firmware[5,6] which was made possible by the selfless dedication of the open source community. Following the debugging sessions, we achieved a stretching accuracy of 0.1 mm, which perfectly meets our experimental requirements.

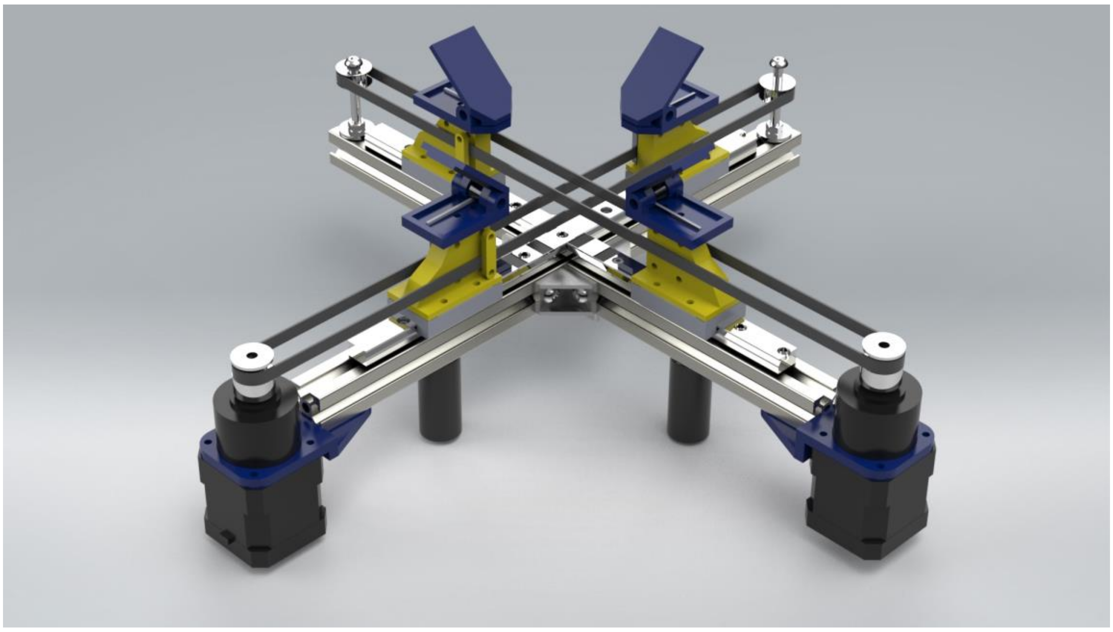

Supplementary Figure 36. Top and bottom isometric isometric view of the 2D stretching machine

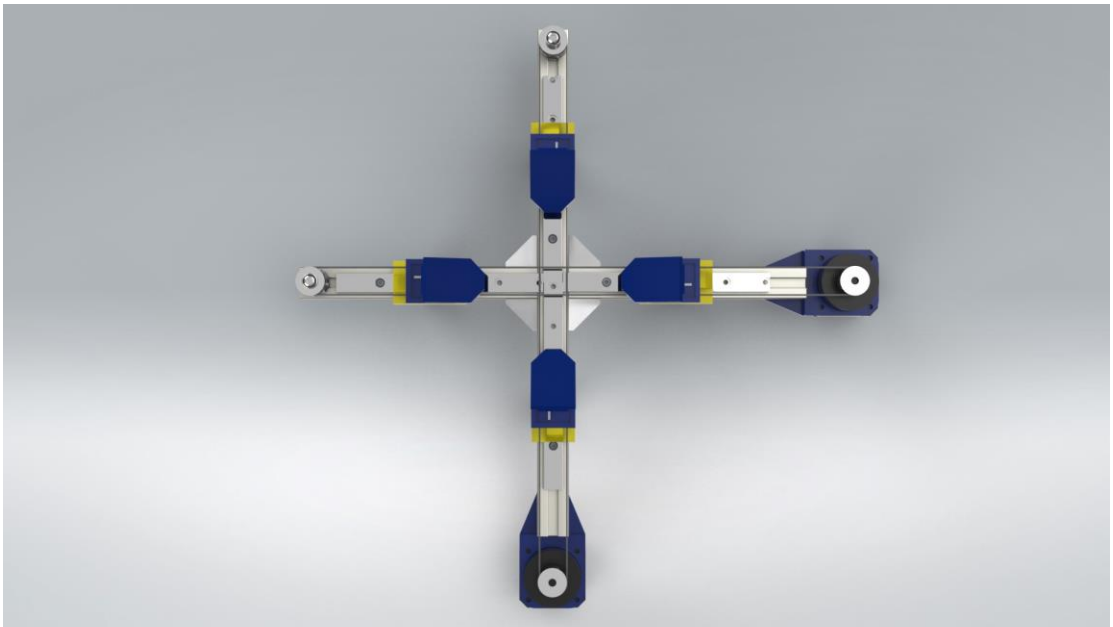

Supplementary Figure 37. Top view of the 2D stretching machine

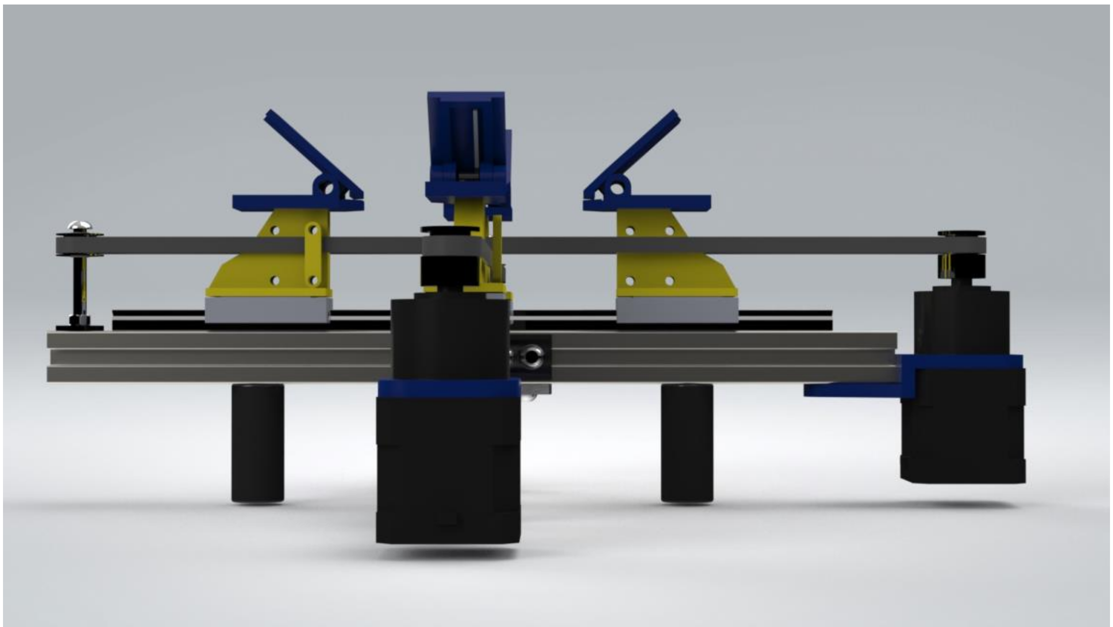

Supplementary Figure 38. Left view of the 2D stretching machine

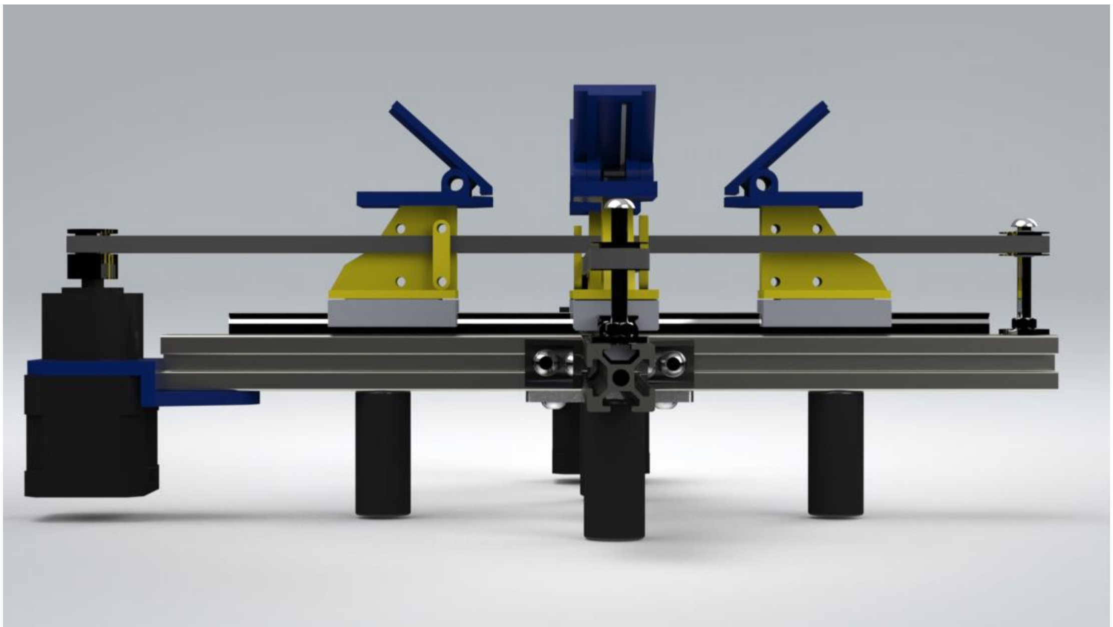

Supplementary Figure 39. Right view of the 2D stretching machine

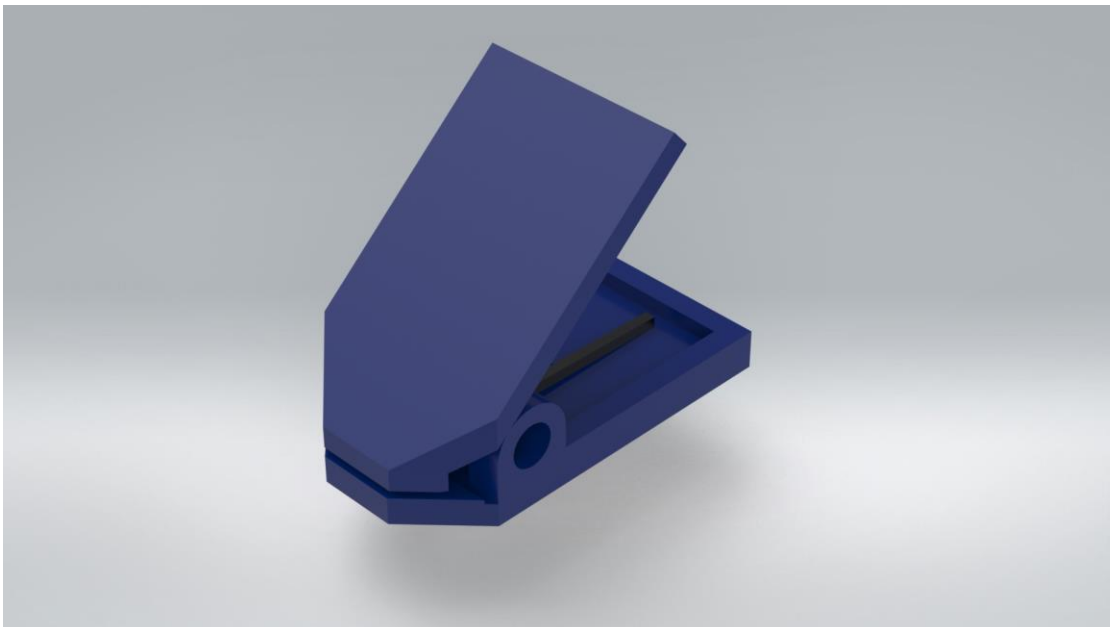

Supplementary Figure 40. Top and bottom isometric isometric view of the fixture

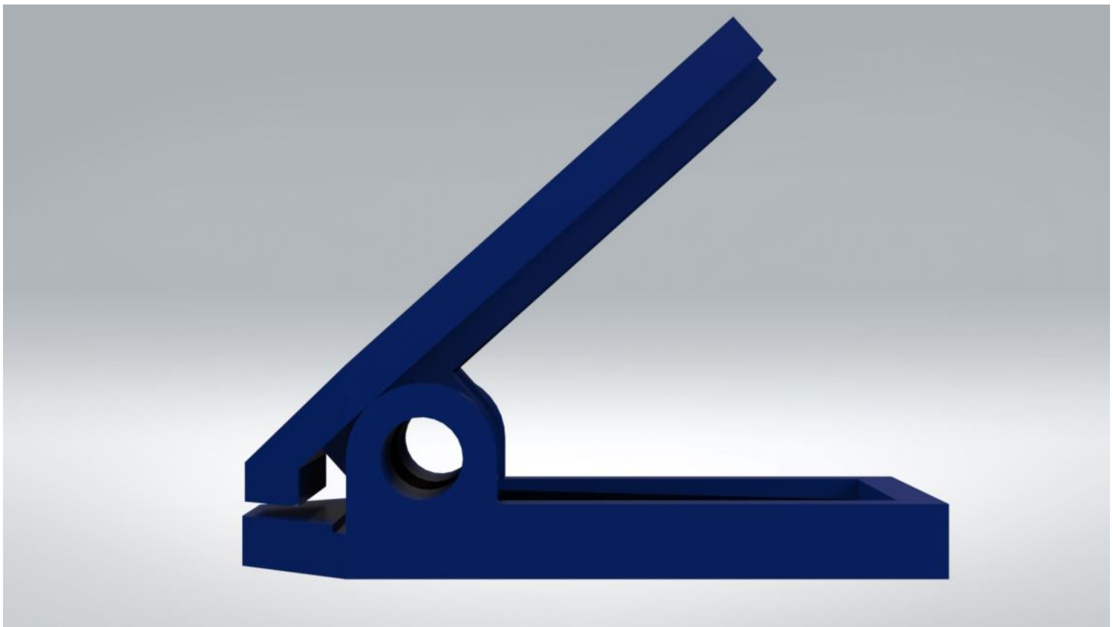

Supplementary Figure 41. Right view of the fixture

Supplementary Note 6: Sample preparation

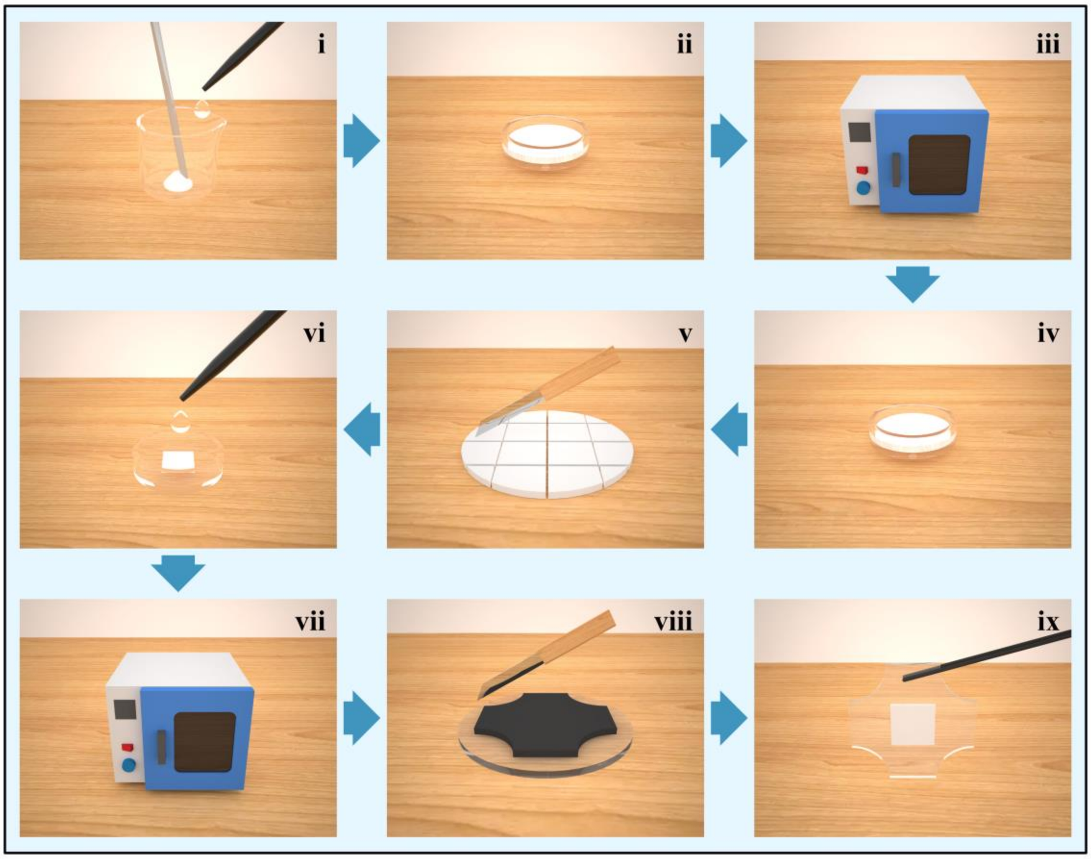

Supplementary Figure 42. Preparation process of the four-leaf clover shape sample

First, weigh and mix the raw materials at a mass ratio of 10 (PDMS matrix) to 1 (PDMS curing agent) to 5.5 (phosphor powder) (i). Transfer the resulting slurry to a 35 mm petri dish (ii) and place it in a 60 °C oven for 5 hours to cure the PDMS (iii). Remove the cured PDMS from the petri dish (iv) and cut it into cubes (v). Take a square of the cured PDMS and place it in another petri dish. Add more PDMS to cover the square (vi), and put the petri dish back in the oven (vii). After curing, take out the sample and cut it according to the mold shape (viii) to obtain the sample (ix).

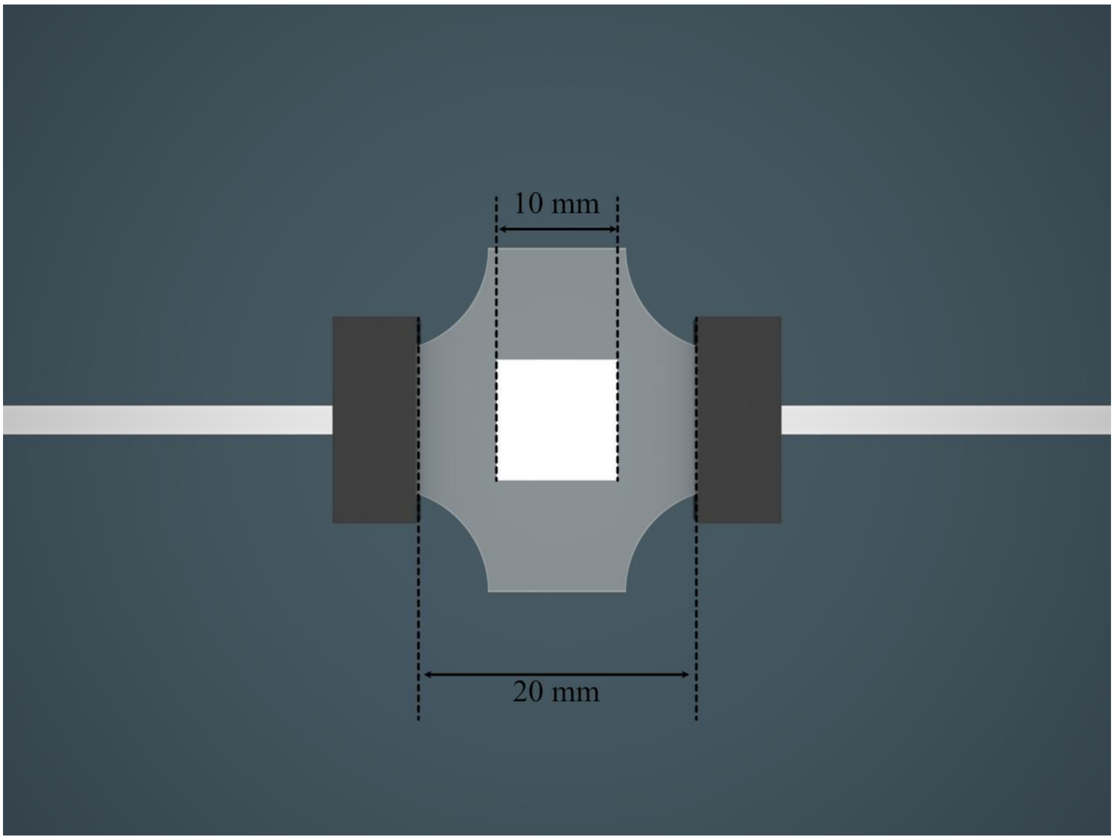

Supplementary Figure 43. The shape and size of the four-leaf clover shape sample

Supplementary Note 7: Properties of $Lu_3Al_5O_{12}$ and Y_2O_2S

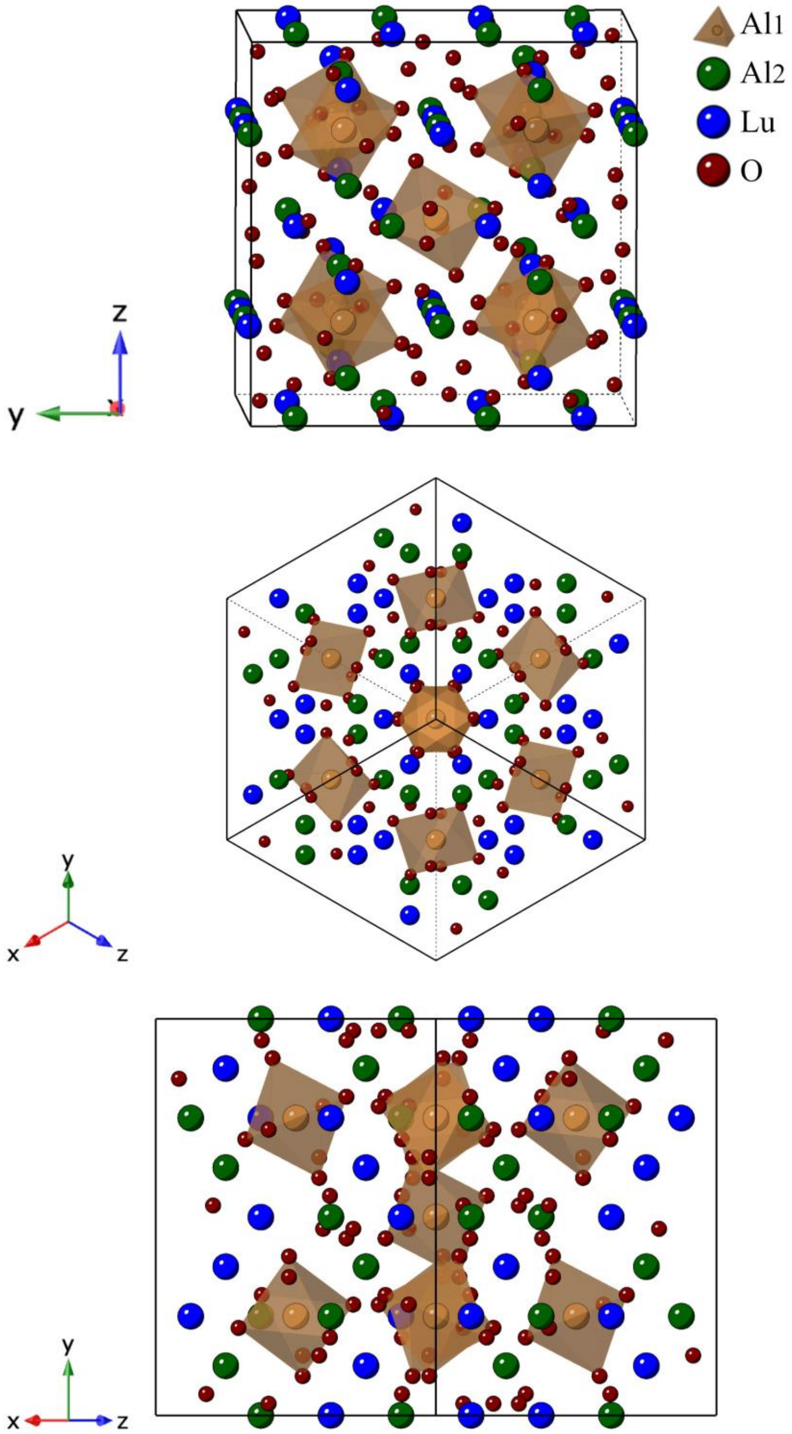

Supplementary Figure 44. Crystal structure of $Lu_3Al_5O_{12}$

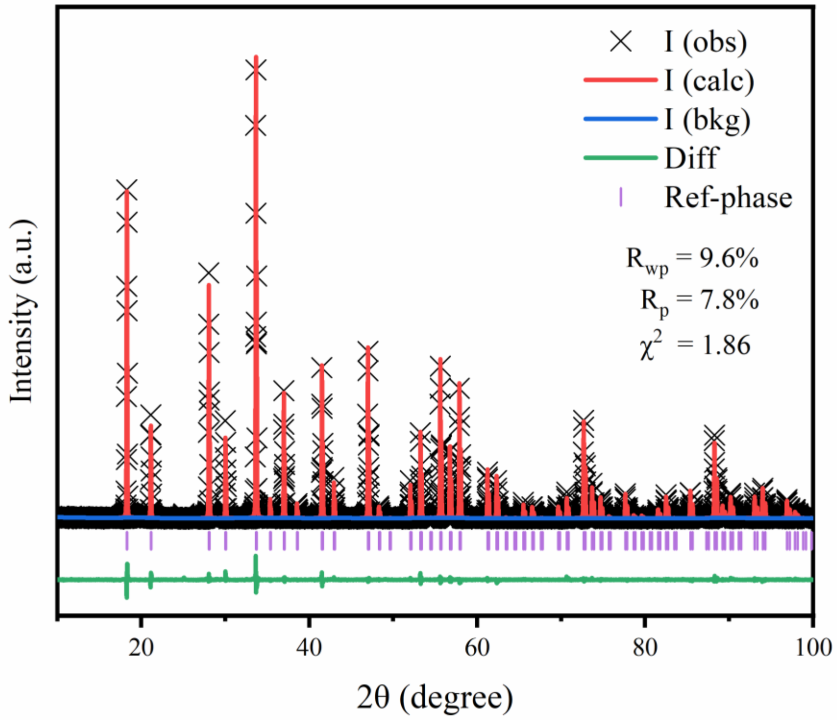

Supplementary Figure 45. Rietveld refinement of the $Lu_3Al_5O_{12}$

Supplementary Table 11. Crystallographic data of $Lu_3Al_5O_{12}$

Formula	$Lu_3Al_5O_{12}$
Crystal system	Cubic
Space group	I a -3 d
Lattice parameters	
a（Å）	11.9203
b（Å）	11.9203
c（Å）	11.9203
α° = β° = γ°	90
Cell volume（Å3 ）	1693.7978
T /K	289
Diffractometer	Rigaku D /Max-2400
Radiation /Å	Cu-Ka（λ= 1.5405）
Absorption correction	multi-scan
2θ range° /	10-100
Z	8
Calculated Density	6.6830 g/cm^3
R-factors	
Rwp	0.096
Rp	0.078

Supplementary Table 12. Refined coordinates of all atoms, bond lengths of X-O and the unit cell parameters of the $Lu_3Al_5O_{12}$

Space group	I a -3 d - Cubic				
Cell	a = b = c = 11.9203				
Ion coordinates	x	y	z	Occupancy	Uiso
Al1	0.000000	0.000000	0.000000	1.0000	0.03356
Al2	0.375000	0.000000	0.250000	1.0000	0.02022
Lu1	0.125000	0.000000	0.250000	1.0000	0.01093
O1	0.031599	0.051802	0.150646	1.0000	0.00573

The bond lengths of X-O			
Vector	Length	Vector	Length
Al1-O1	1.936(8)	Lu1_O1	2.295(7)
Al2_O1	1.739(8)	Lu1_O1	2.412(10)

Supplementary Table 13. The bandgap of $Lu_3Al_5O_{12}$ Calculated by Vienna Ab-initio Simulation Package (VASP)

Band Character	Direct
Band Gap (eV)	5.0676
Eigenvalue of VBM (eV)	3.5630
Eigenvalue of CBM (eV)	8.6306
Fermi Energy (eV)	3.7840
HOMO & LUMO Bands	228 229
Location of VBM	0.000000 0.000000 0.000000
Location of CBM	0.000000 0.000000 0.000000

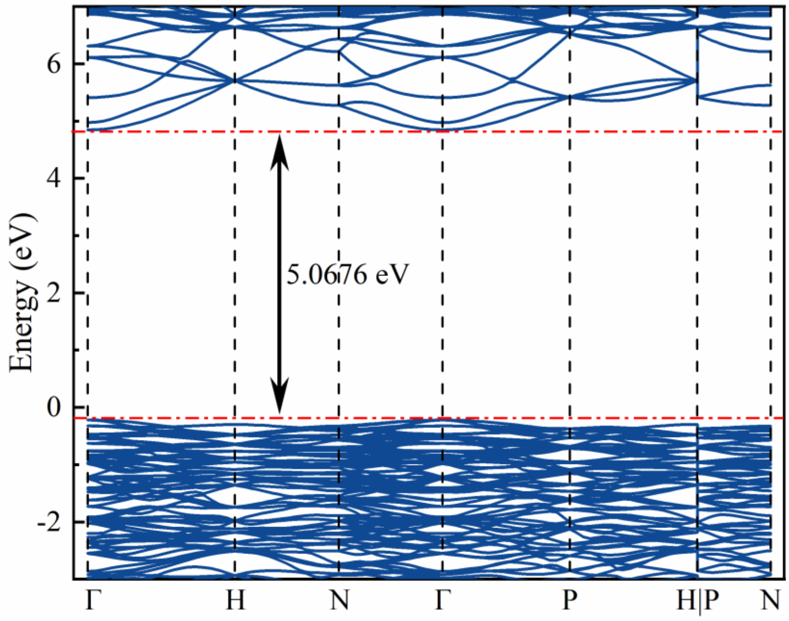

Supplementary Figure 46. Calculated energy band structure of $Lu_3Al_5O_{12}$

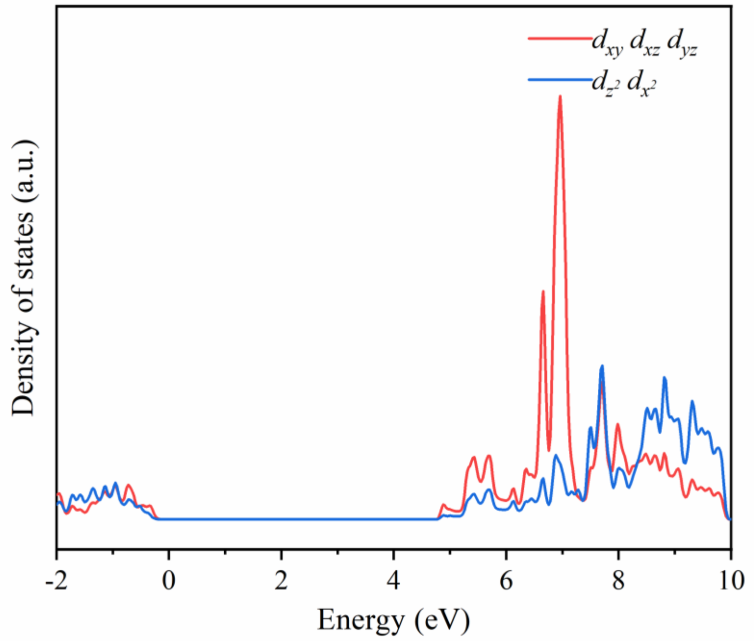

Supplementary Figure 47. Partial density of states (Lu atoms) of $Lu_3Al_5O_{12}$

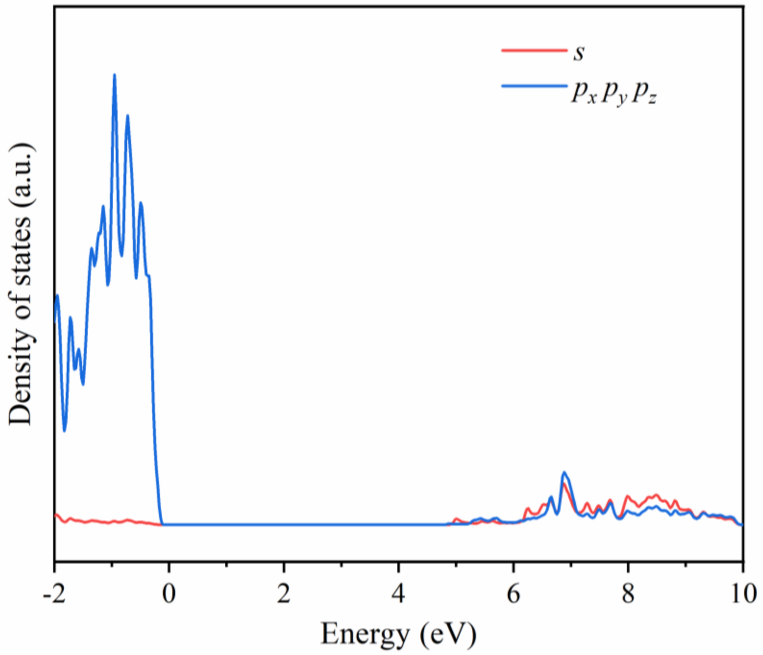

Supplementary Figure 48. Partial density of states (O atoms) of $Lu_3Al_5O_{12}$

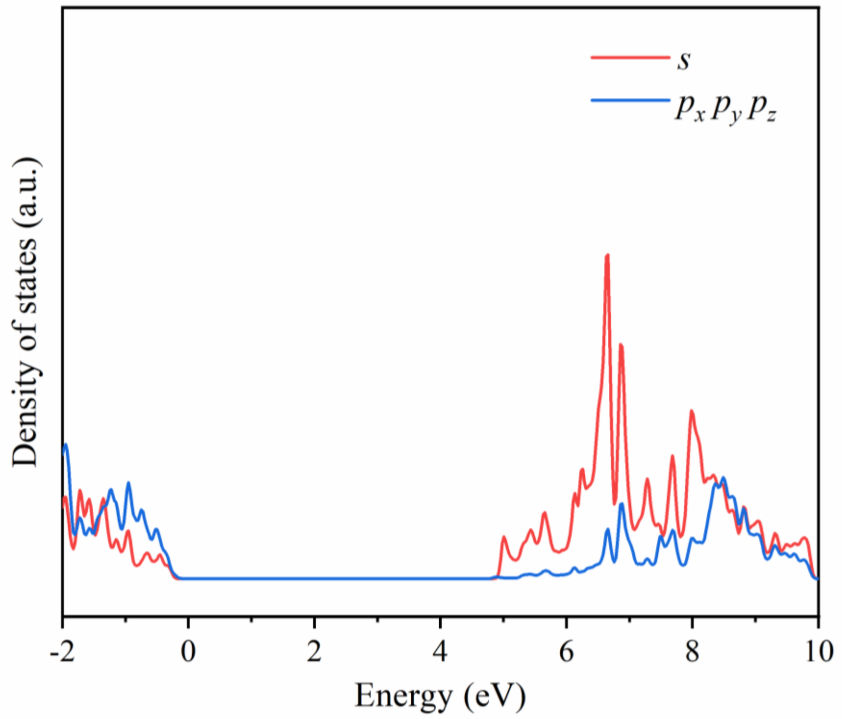

Supplementary Figure 49. Partial density of states (Al atoms) of $Lu_3Al_5O_{12}$

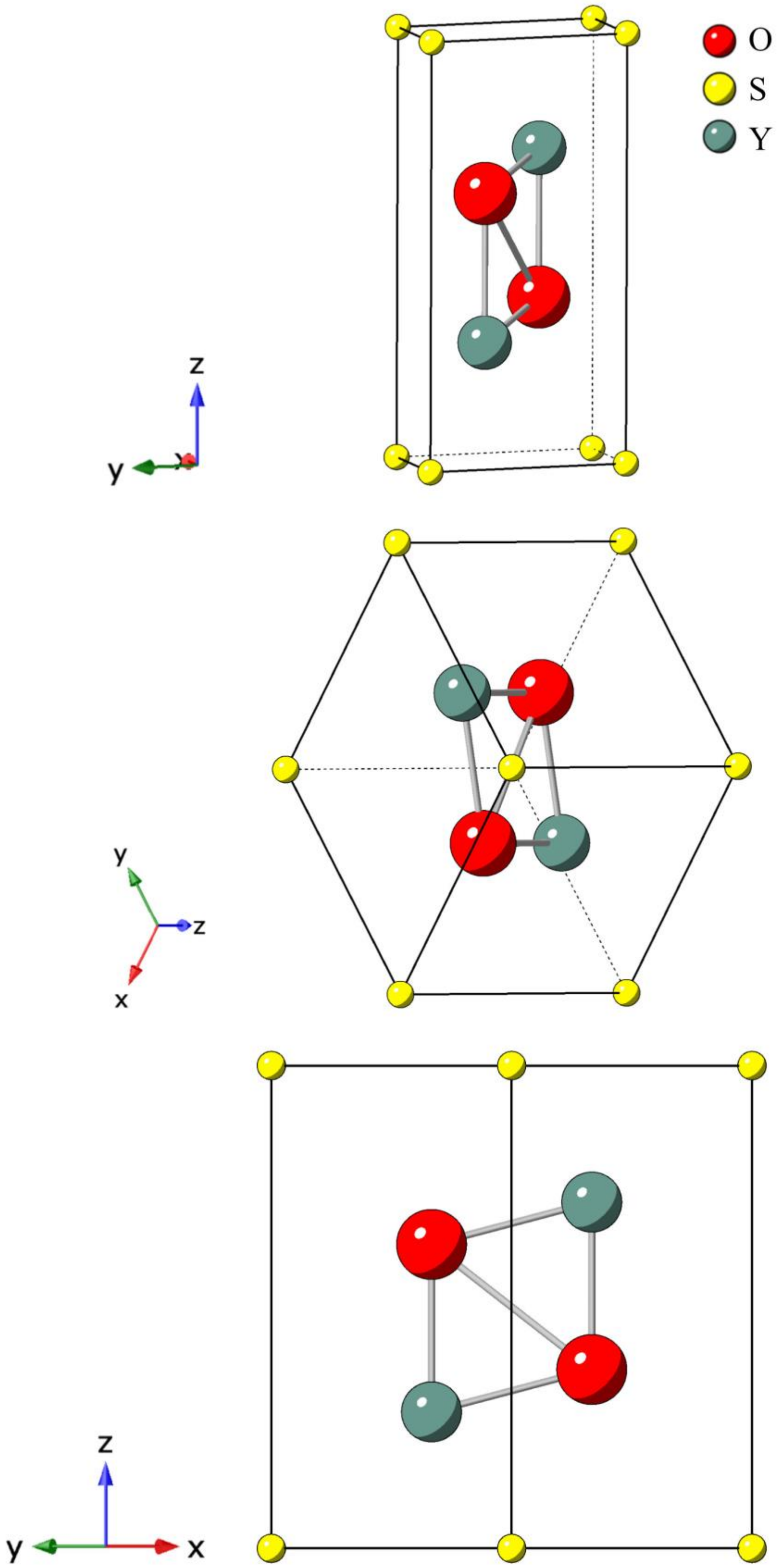

Supplementary Figure 50. Crystal structure of Y_2O_2S

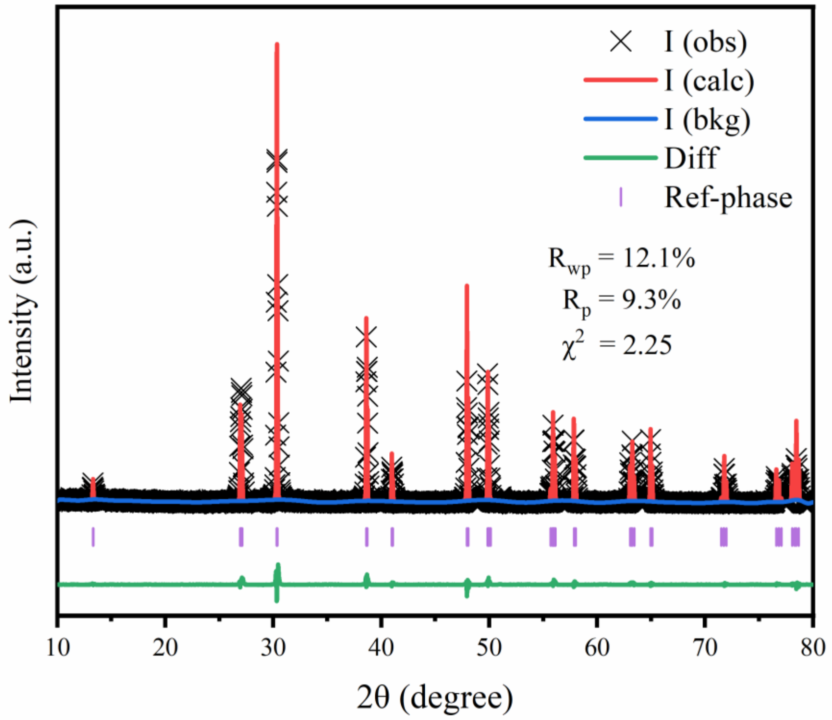

Supplementary Figure 51. Rietveld refinement of the Y_2O_2S

Supplementary Table 14. Crystallographic data of Y_2O_2S

Formula	Y_2O_2S
Crystal system	Trigonal
Space group	P -3 m 1
Lattice parameters	
a（Å）	3.7817
b（Å）	3.7817
c（Å）	6.5822
α° = β°	90
γ°	120
Cell volume（Å3 ）	81.521
T /K	289
Diffractometer	Rigaku D /Max-2400
Radiation /Å	Cu-Ka（λ= 1.5405）
Absorption correction	multi-scan
2θ range° /	10-80
Z	1
Calculated Density	4.9270 g/cm^3
R-factors	
Rwp	0.1205
Rp	0.0932

Supplementary Table 15. Refined coordinates of all atoms, bond lengths of X-O/S and the unit cell parameters of the Y_2O_2S

Space group	P -3 m 1 - Trigonal				
Cell	a = b = 3.7817 c = 6.5822				
Ion coordinates	x	y	z	Occupancy	Uiso
Y1	0.333300	0.666700	0.282000	1.0000	0.00032
O1	0.333300	0.666700	0.631000	1.0000	0.00032
S1	0.000000	0.000000	0.000000	1.0000	0.00032
The bond lengths of X-O/S					
Vector	Length	Vector	Length		
Y1_O1	2.29719	Y1_S1	2.86582		
Y1_O1	2.25699	Y1_S1	2.86557		
Y1_O1	2.25762				

Supplementary Table 16. The bandgap of Y_2O_2S Calculated by Vienna Ab-initio Simulation Package (VASP)

Band Character	Indirect
Band Gap (eV)	3.0457
Eigenvalue of VBM (eV)	3.5572
Eigenvalue of CBM (eV)	6.6029
Fermi Energy (eV)	3.7839
HOMO & LUMO Bands	20 21
Location of VBM	0.000000 0.000000 0.500000
Location of CBM	0.333333 0.333333 0.000000

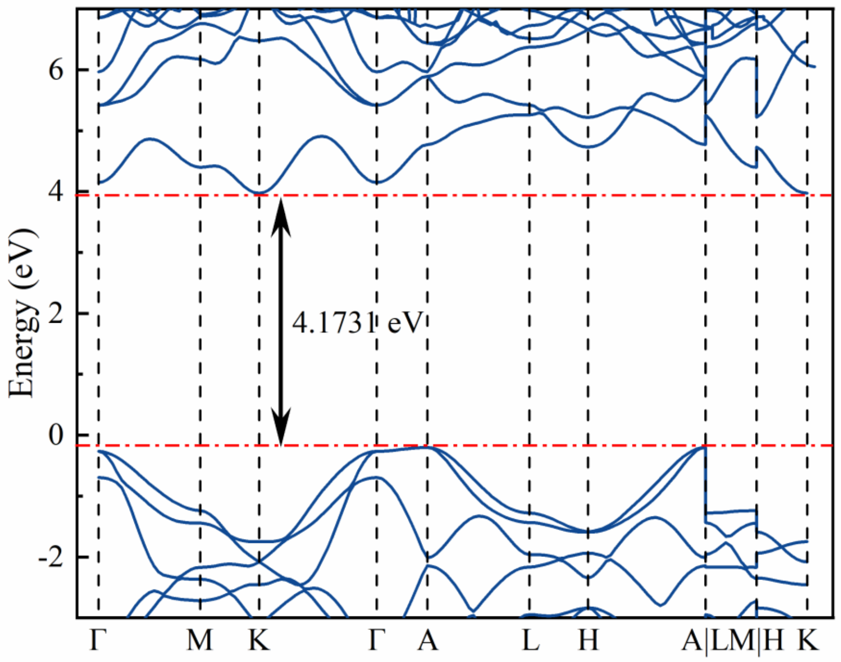

Supplementary Figure 52. Calculated energy band structure of Y_2O_2S

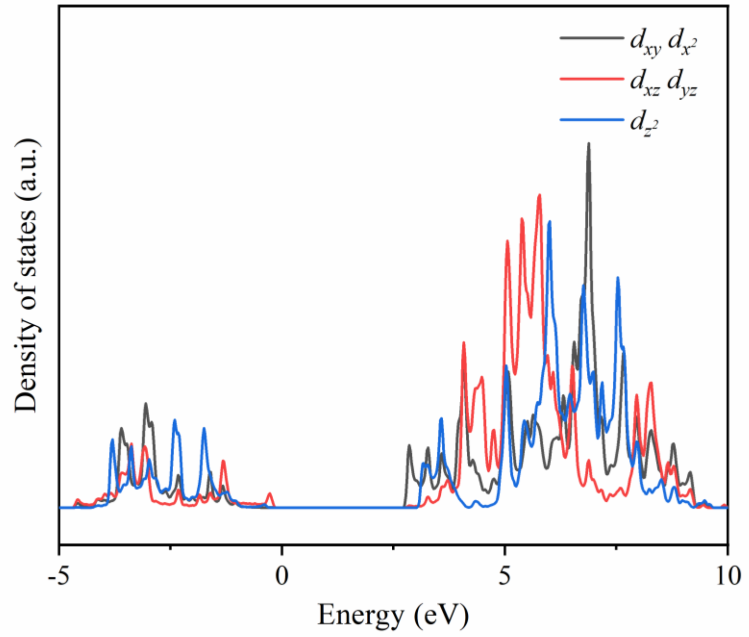

Supplementary Figure 53. Partial density of states (Y atoms) of Y_2O_2S

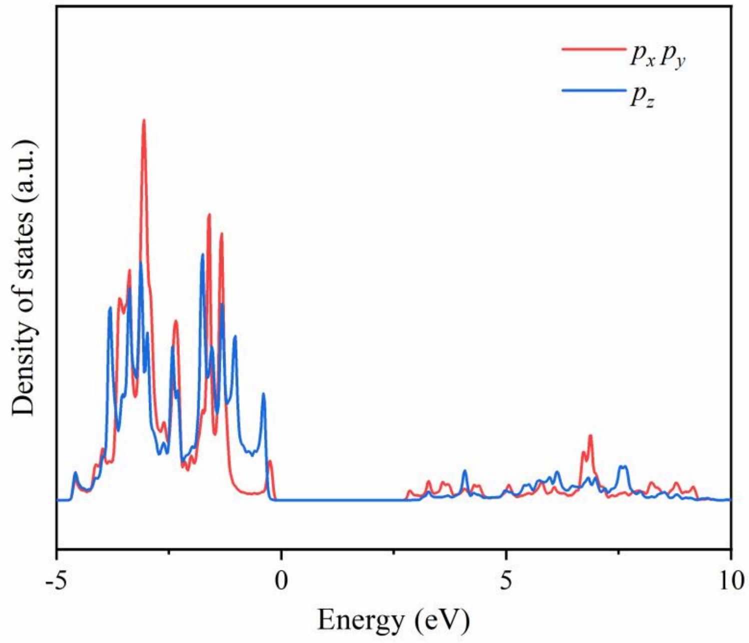

Supplementary Figure 54. Partial density of states (O atoms) of Y_2O_2S

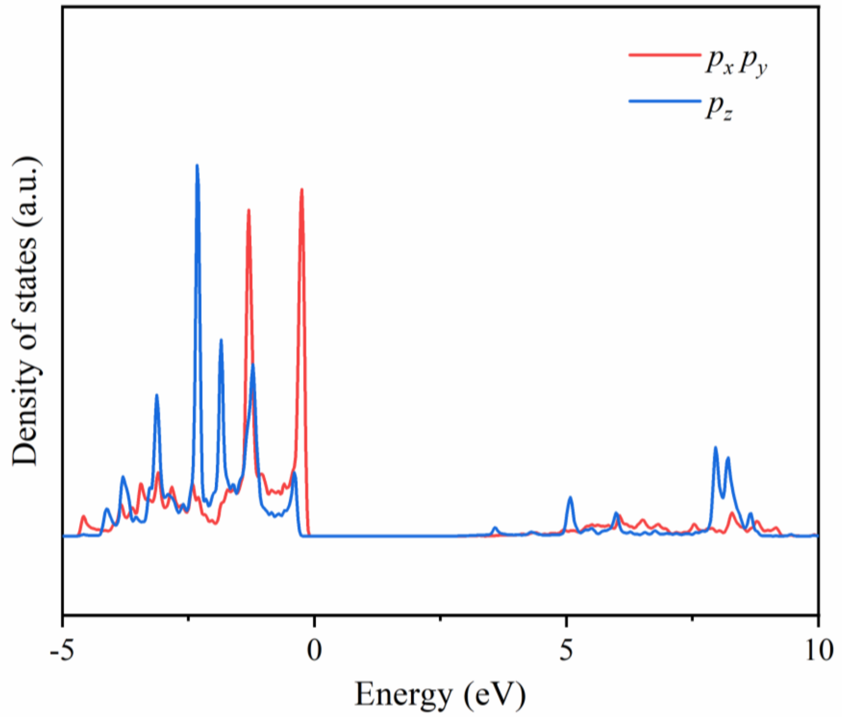

Supplementary Figure 55. Partial density of states (S atoms) of Y_2O_2S

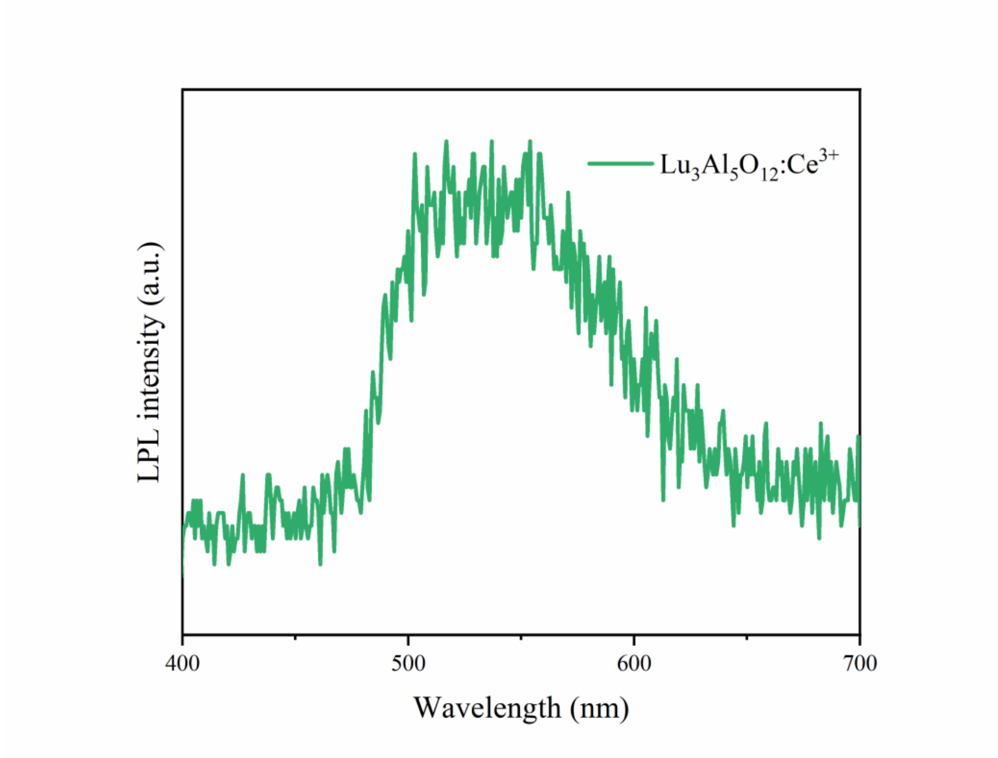

Supplementary Figure 56. LPL spectrum of $Lu_3Al_5O_{12}:Ce^{3+}$

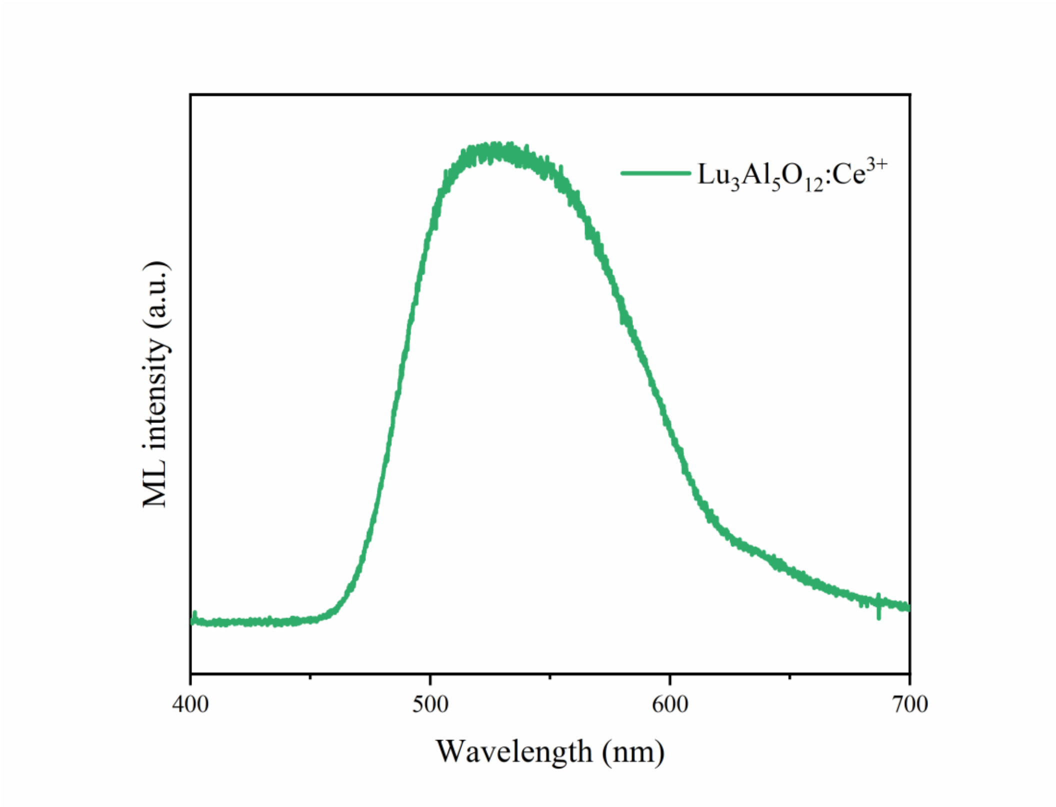

Supplementary Figure 57. ML spectrum of $Lu_3Al_5O_{12}:Ce^{3+}$

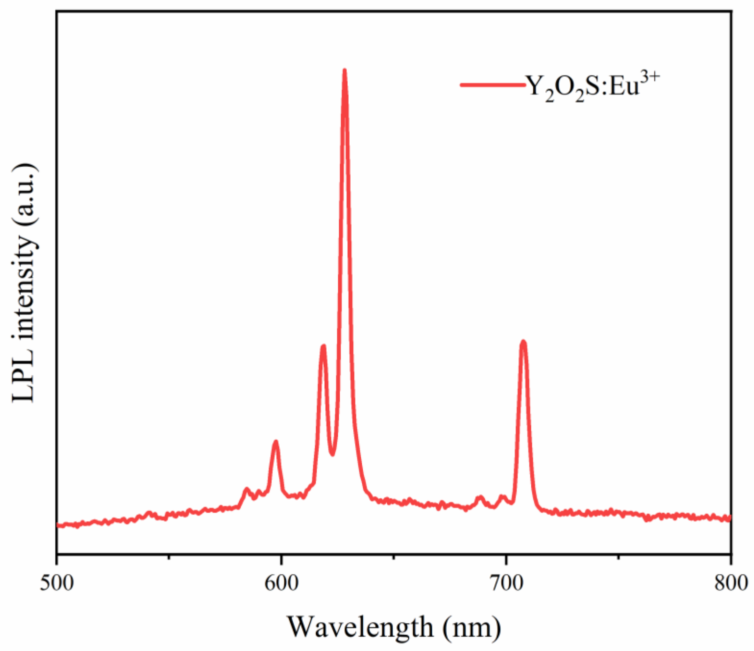

Supplementary Figure 58. LPL spectrum of $Y_2O_2S:Eu^{3+}$

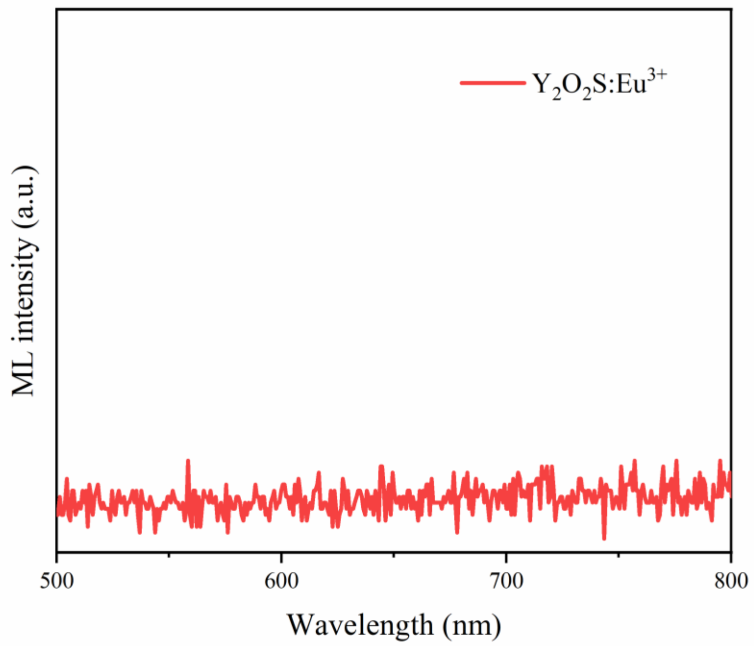

Supplementary Figure 59. ML spectrum of $Y_2O_2S:Eu^{3+}$

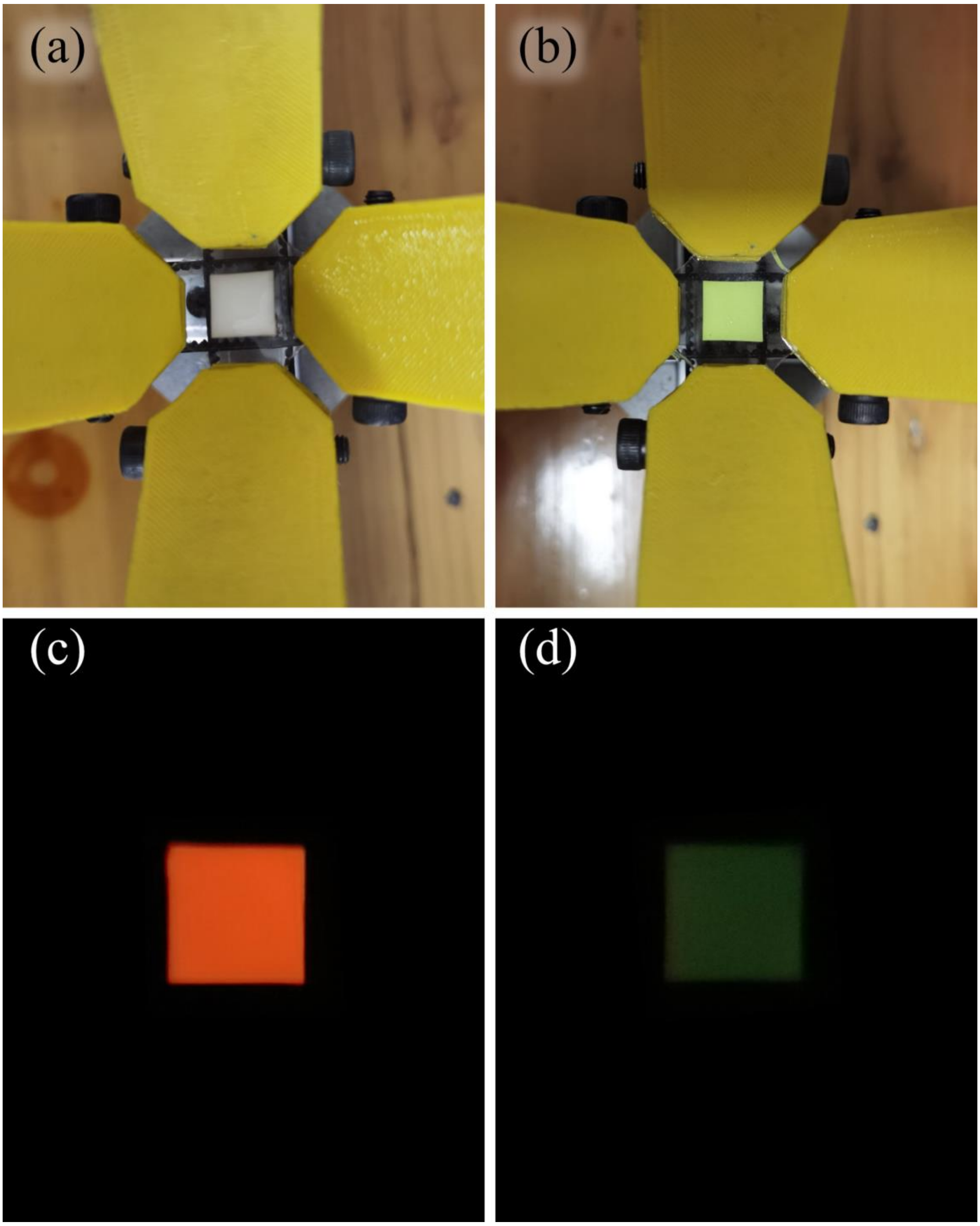

Supplementary Figure 60. Bright field photographs(a, b) and dark field LPL photographs(c, d) of $Y_2O_2S{:}Eu^{3+}$/PDMS(a, c) and $Lu_3Al_5O_{12}{:}Ce^{3+}$/PDMS(b, d) elastomers

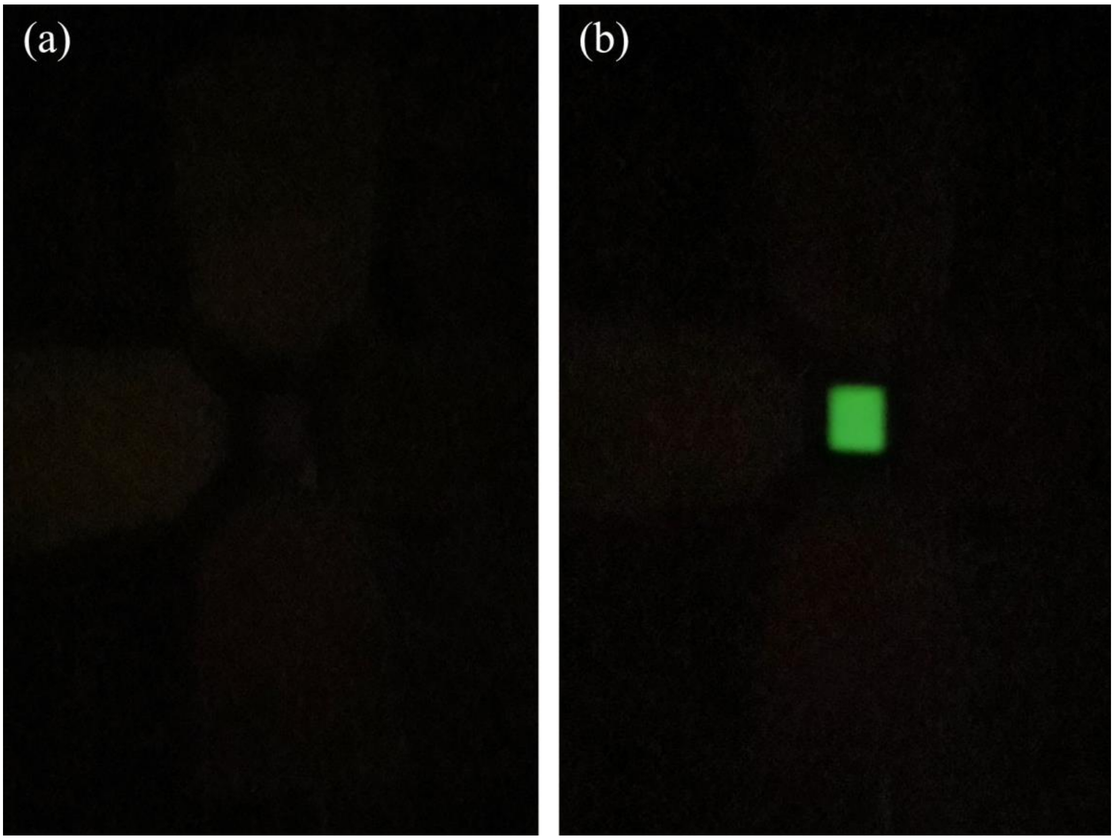

Supplementary Figure 61. ML photographs of $Y_2O_2S:Eu^{3+}$/PDMS(a) and $Lu_3Al_5O_{12}:Ce^{3+}$/PDMS(b) elastomers

$Lu_3Al_5O_{12}:Ce^{3+}$ is a commercially available phosphor with a centrosymmetric structure that exhibits excellent ML properties when used in a PDMS composite, as demonstrated in our previous work.[7] Interestingly, it was found that the ML properties of this phosphor are independent of the trap level, making it an ideal candidate for comparison. In contrast, $Y_2O_2S:Eu^{3+}$ is another commercially available phosphor with a centrosymmetric structure that is known for its excellent long persistent performance. While some reports have suggested that this phosphor exhibits ML, we did not observe any ML in the $Y_2O_2S:Eu^{3+}$/PDMS composite(Supplementary Figure 59, 61), possibly due to the lower tensile and tear strengths of PDMS. Therefore, this phosphor was chosen to represent sulfide in our study.

Supplementary Note 8: Influence of light exposure on ML

In general, for the previously reported long-persistent luminescence phosphors represented by $SrAl_2O_4:Eu^{2+}$, their PDMS-based elastomers always require sufficient UV/blue pre-irradiation to charge traps, and then stored carriers in the traps can be stimulated for ML under external stress. Therefore, it always requires to strictly avoid the influences of the indoor fluorescent or sunlight exposure. In this work, two strategies were adopted to rule out the potential influence of the indoor fluorescent or sunlight exposure on the ML properties of the samples.

First, all experiments in this work including the phosphor synthesis, elastomer preparation and ML measurement were carried out in a dark room illuminated by a red LED lamp covering 680-690 nm (Boxing Corp: YS-GS-MR-00, 10 W).

Second, our experiments (Supplementary Figure 62) demonstrate that ML properties of the new $CaF_2:Tb^{3+}$/PDMS elastomers are completely unaffected even when directly exposed to ultraviolet or blue light. Unlike the previously reported long-persistent luminescence phosphors, where ML always depends on the carrier detrapping in traps, ML of this new $CaF_2:Tb^{3+}$/PDMS elastomer is excited by the contact electrification, so its ML is only related to the interaction between the phosphors and PDMS, and is completely independent of whether it has been irradiated by fluorescent or sunlight exposure containing ultraviolet or blue light.

As evidence, we conducted a serial of comparative experiments and prepared five identical $CaF_2:Tb^{3+}$/PDMS elastomers. One was not exposed, while the others were sufficiently exposed to different lights (254/365/405 nm) and natural sunlight for 10 minutes, respectively. Accordingly, Supplementary Figure 62 (a) presents the ML intensities of these elastomers at the first stretch, and it can be seen that there is no significant difference. Furthermore, Supplementary Figure 62 (b) presents the normalized ML intensities of these $CaF_2:Tb^{3+}$/PDMS elastomers under continuous stretching. It demonstrates that all these elastomer samples show the similar ML decrease curves. Supplementary Figure 62 (c) also exhibits the photographs of these elastomers under different irradiations and their ML photographs at the first stretch. It can be seen that the sufficient irradiation exposure does not significantly affect the ML properties of the $CaF_2:Tb^{3+}$/PDMS elastomer samples. In fact, the contact-separation-induced ML of this new $CaF_2:Tb^{3+}$/PDMS elastomer is only affected by the interaction between the phosphors and PDMS, and is not affected by the irradiation exposure.

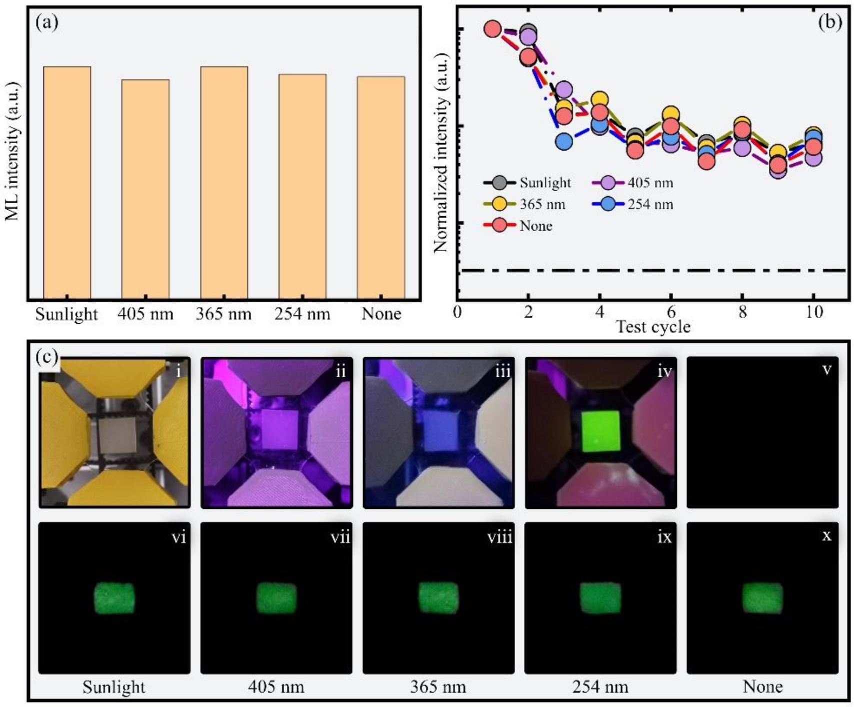

Supplementary Figure 62. a) ML intensities of the CaF_2:Tb^{3+}/PDMS elastomers at the first stretch; b) normalized ML intensities of the elastomers under continuous stretching; c) photographs of the elastomers under different irradiations and their ML photographs at the first stretch

Supplementary Note 9: Differences of scratching and stretching

In this case, ML can be clearly observed when the pressure applied to the CaF_2:Tb^{3+}/PDMS elastomer is greater than 0.2 MPa. Generally, the entire elastomer is stressed under stretching, while only a very tiny area in contact with the nail is stressed under scratching. Therefore, the stress-bearing area of the elastomer under stretching is much larger than that under scratching. Consequently, even if the same pressure is applied, the stress under stretching must be much greater than the stress under scratching due to the much larger stress-bearing area. Correspondingly, Supplementary Table 17 shows the stretching stresses and scratching stresses to the CaF_2:Tb^{3+}/PDMS elastomer under different pressures. For example, it shows that when the pressure applied to the elastomer is 0.2341 MPa, the stretching stress (2.5571 N) is even more than 20 times the scratching stress (0.1176 N). Consequently, we always feel that the stress under scratching is very "slight" and the stress under stretching is very "hard".

Supplementary Table 17. Stretching stresses and scratching stresses to the CaF_2:Tb^{3+}/PDMS elastomer under different pressures.

Pressure (MPa)	Stretching stress (N)	Scratching stress (N)
0.1261	1.3778	0.0634
0.2341	2.5571	0.1176
0.3304	3.6101	0.1660
0.4215	4.6051	0.2118
0.5074	5.5434	0.2549
0.5926	6.4743	0.2977
0.6798	7.4264	0.3415
0.7729	8.4442	0.3883
0.8799	9.6127	0.4421
1.0013	10.9390	0.5030

Supplementary Note 10: Analysis of the contact interface

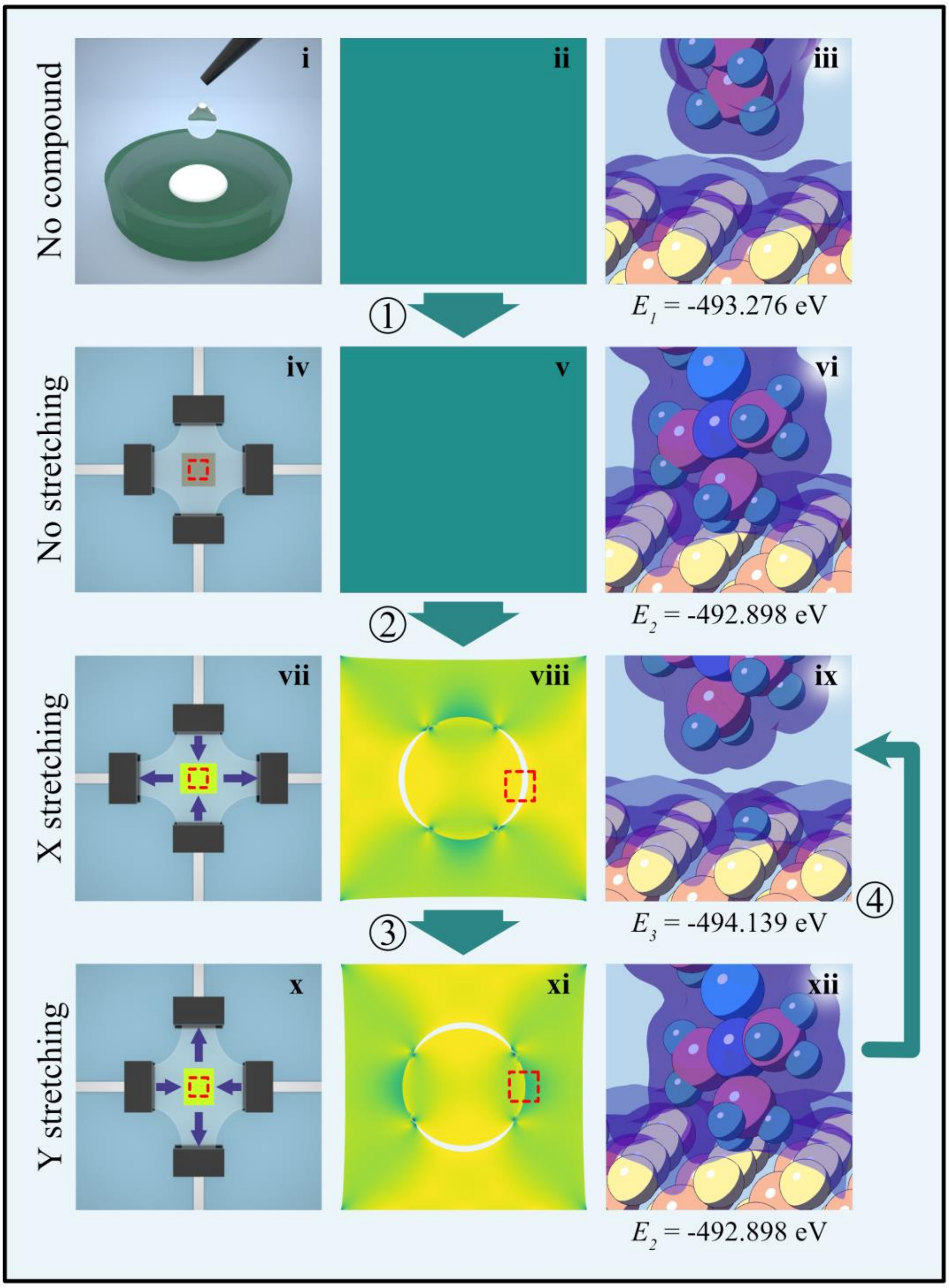

Supplementary Figure 63. Stress distribution and binding energies of the elastomer

Supplementary Figure 63 illustrates the states and binding energies of the complex at different stages during two-dimensional stretching. Originally, prior to incorporating the phosphor into PDMS (Supplementary Figure 63 i), there was no contact between

the phosphor and PDMS, hence no interfacial stress (Supplementary Figure 63 ii). The binding energy of the complex was E_1 = -493.276 eV (Supplementary Figure 63 iii). Upon incorporating the phosphor into PDMS (Supplementary Figure 63 iv), the phosphor and PDMS were in contact but without interfacial stress (Supplementary Figure 63 v). The binding energy of the complex was E_2 = -492.898 eV (Supplementary Figure 63 vi). Subsequently, the complex was stretched horizontally (Supplementary Figure 63 vii) and concurrently subjected to tensile forces in the horizontal direction and compressive forces in the vertical direction (Supplementary Figure 63 viii). Horizontally, the phosphor and PDMS were stretched apart. Calculation showed that the complex had the lowest binding energy when hydrogen atoms remained on the surface of the phosphor. The binding energy at this stage was E_3 = -494.139 eV (Supplementary Figure 63 ix), releasing energy of ΔE_{23} = 1.241 eV. The complex was then stretched vertically (Supplementary Figure 63 x) and simultaneously subjected to tensile forces in the vertical direction and compressive forces in the horizontal direction (Supplementary Figure 63 xi). Under the effect of horizontal compression , the phosphor and PDMS came into close contact again. At this point, the complex absorbed energy of ΔE_{23} = 1.241 eV and returned to E_2, completing one two-dimensional stretching cycle.

In summary, during the cycling process, energy was released when the phosphor and PDMS separated and absorbed when they came into contact again. The requisite energy was provided by the external tensile forces.

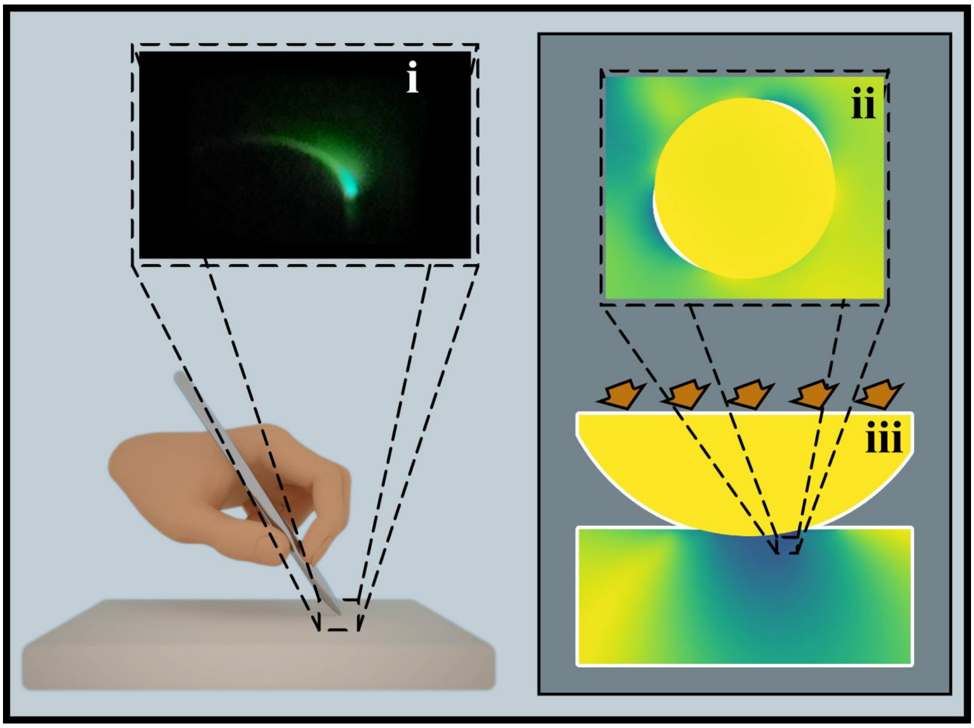

Supplementary Figure 64. The stress distribution and ML photo for the elastomer under stretching

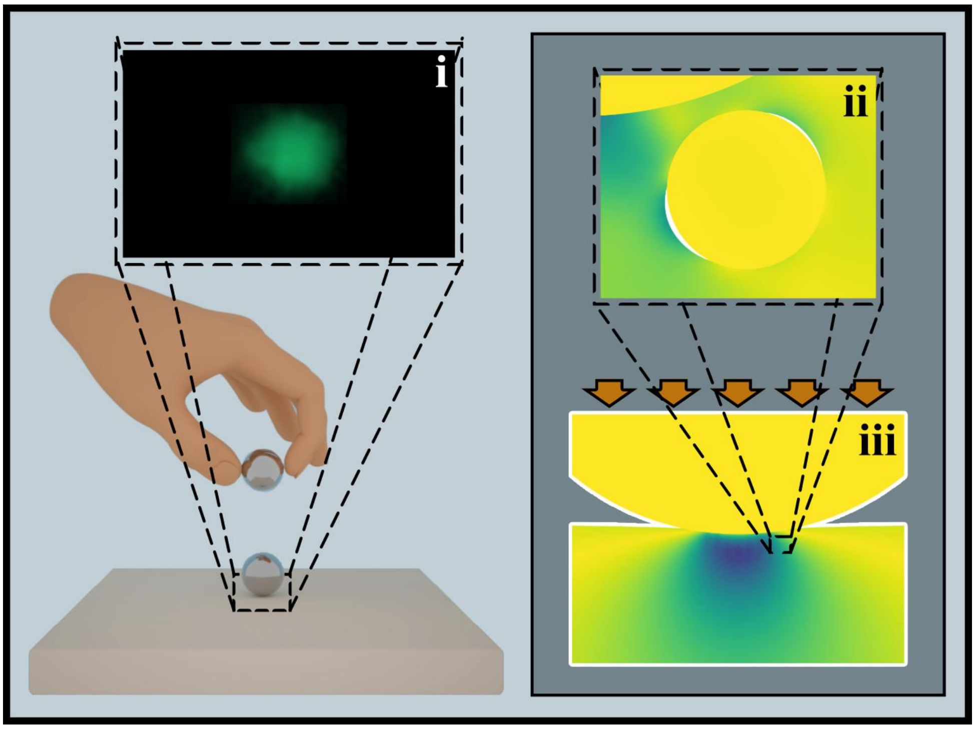

Supplementary Figure 65. The stress distribution and ML photo for the elastomer under falling ball

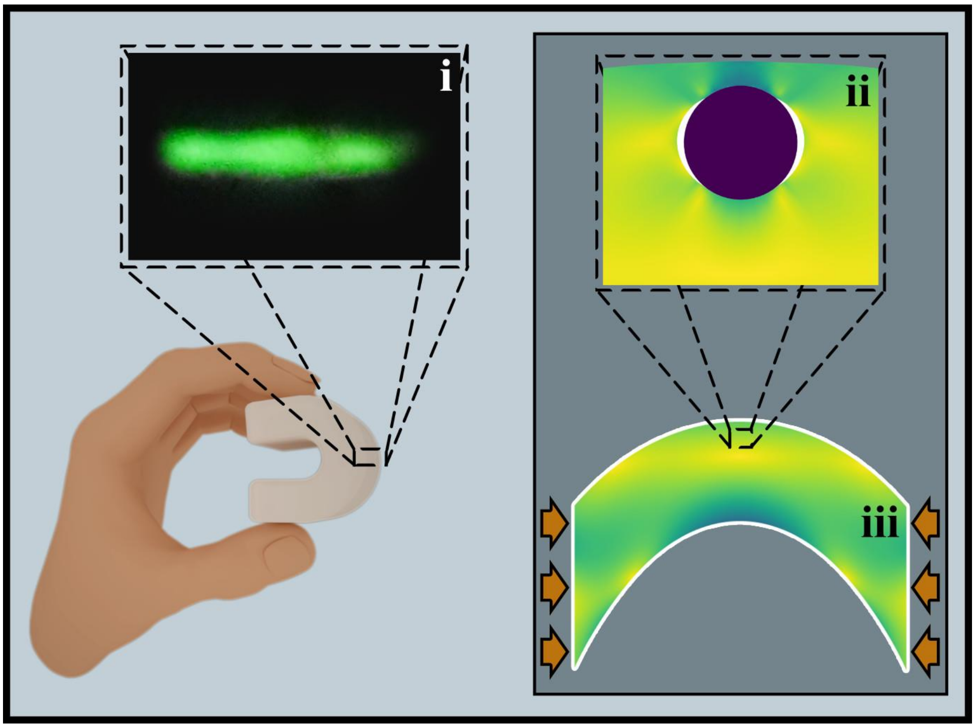

Supplementary Figure 66. The stress distribution and ML photo for the elastomer under bending

Supplementary Figure 63-66 present ML images of the complexes under different action modes, along with the corresponding stress distribution at the interface. The Figures indicate that the action modes which elicit ML also result in the separation of phosphor powder from the PDMS.

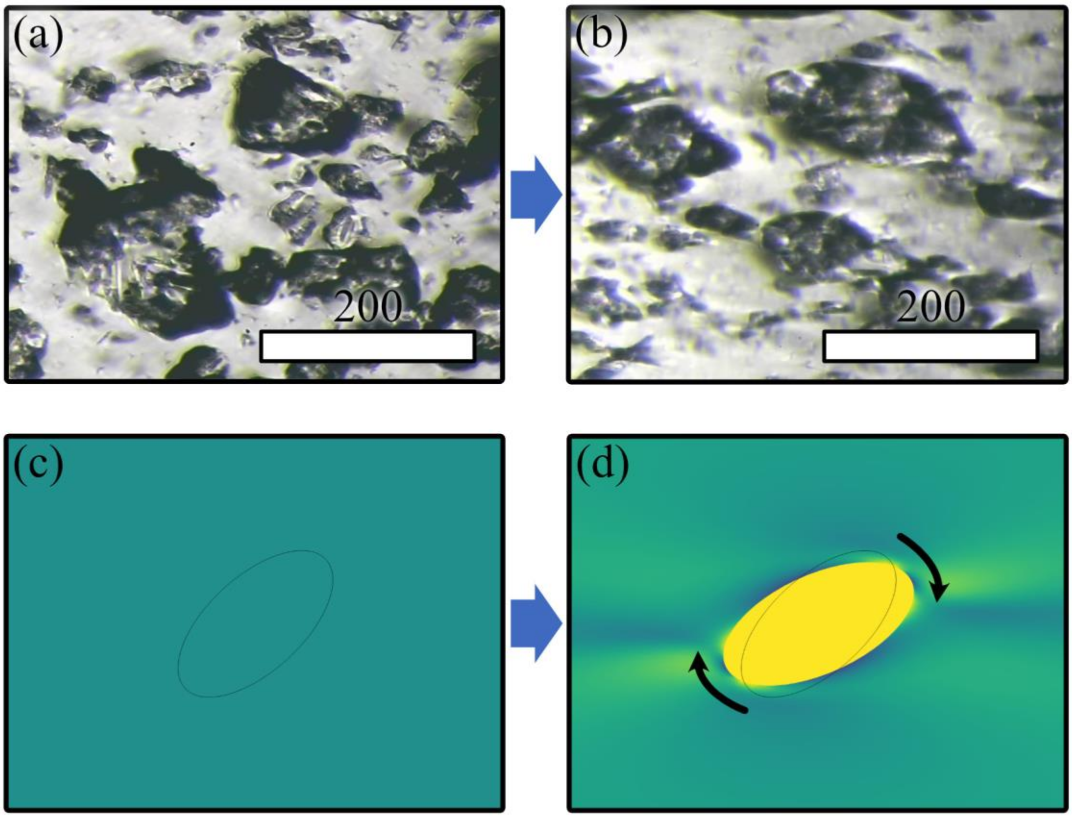

Supplementary Figure 67. In situ micrographs(a, b) and stress distribution(c, d) before(a, c) and after(b, d) stretching

When stretched, the randomly arranged phosphor particles (Supplementary Figure 67 (a)) tend to become ordered, as shown in Supplementary Figure 67 (b), which could be simulated by the finite element method as shown in Supplementary Figure 67 (c-d).

Supplementary Note 11: ML performance during a single stretching

Supplementary Figure 68 (i) exhibits the ML intensities and stretching distances of the $CaF_2:Tb^{3+}$/PDMS elastomer during a single stretching. It indicates that the ML of the $CaF_2:Tb^{3+}$/PDMS elastomer only happens once during the stretching process, and we can never observe any ML when the stretched elastomer is released. However, as a comparison, the ML of the ZnS:Cu/PDMS elastomer can be detected during both the stretching and releasing processes, as shown in Supplementary Figure 68 (ii). Since the ML of $CaF_2:Tb^{3+}$/PDMS elastomer is due to contact electrification, it is understandable that its ML can be only observe once when the contacted surfaces of the phosphors and PDMS are separated during the stretching process. However, the ML of the ZnS:Cu/PDMS elastomer is essentially associated with strain-induced piezoelectricity. Generally, strain can be created during both stretching and releasing processes of the elastomers, resulting in piezoelectricity. Therefore, the ML of the ZnS:Cu/PDMS elastomer can be also observed during both the stretching and releasing processes.

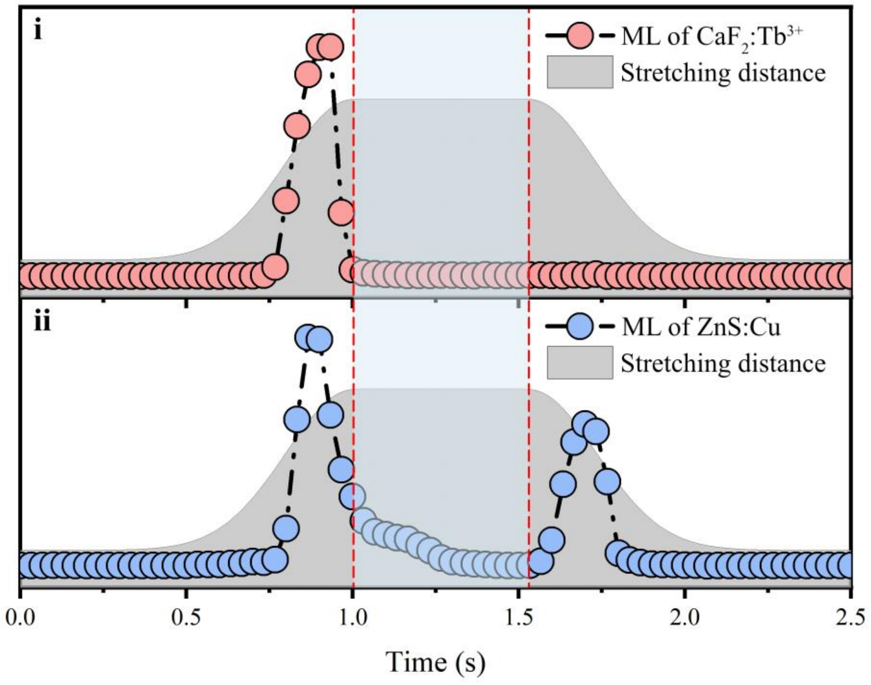

Supplementary Figure 68. ML intensities and stretching distances of the $CaF_2:Tb^{3+}$/PDMS (i) and ZnS:Cu/PDMS (ii) elastomers during a single stretching.

As shown in Supplementary Figure 68, the areas marked with red dotted lines correspond to the ML performances of the CaF_2:Tb^{3+}/PDMS (i) and ZnS:Cu/PDMS (ii) elastomers when one holds the elastomer in the stretched position. The experiment results show that we cannot observe any ML when the CaF_2:Tb^{3+}/PDMS (i) elastomer is held in the stretched position, while the ML can be still detected when the ZnS:Cu/PDMS (ii) elastomers is held. According to contact electrification, carrier recombination occurs only at the moment of surface separation during the stretching process. When the elastomer is held in the stretched position, the surfaces of the phosphors and PDMS have been separated during the previous stretching process and therefore we can no longer observe the ML. However, it is well known that piezoelectricity is due to the stress-induced strain in a material. Even if the elastomer is held in the stretched position, there is still stress-induced strain in the elastomer, so we can still observe the piezoelectricity-induced ML of the ZnS:Cu/PDMS (ii) elastomer.

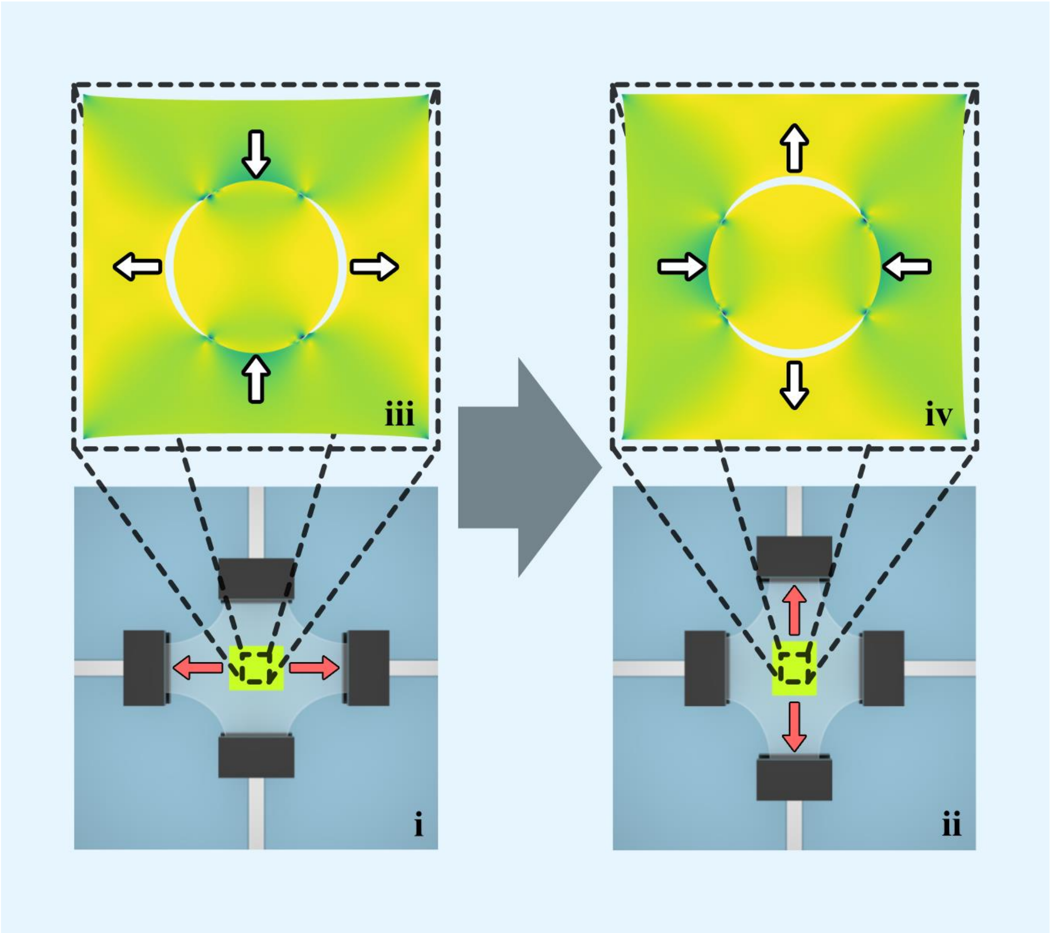

Supplementary Figure 69. Schematic diagram and finite element stress (von Mises) simulations of the CaF_2:Tb^{3+}/PDMS elastomer under a two-dimensional stretching.

Moreover, Supplementary Figure 69 presents the schematic diagram and finite element stress (von Mises) simulations of the CaF_2:Tb^{3+}/PDMS elastomer under a two-dimensional stretching. It shows that when the elastomer is stretched horizontally in the x direction (i), the internal phosphor particles and PDMS will be separated by the horizontal stretching stress, while they contact due to the contraction of the elastomer in the vertical direction (iii). Then, when the elastomer is vertically stretched in the y direction (ii), the previously contacted surfaces of the phosphor and PDMS in the vertical direction will be separated under stretching stress, while the previously separated surfaces in the horizontal direction will be recontacted due to the contraction of the elastomer, as shown in Supplementary Figure 69 (iv). This result shows that surface separation occurs during the stretching process, resulting in carrier recombination and ML of the elastomers, while surface contact occurs during the contracting/releasing process, corresponding to electron transfer due to contact electrification. Therefore, nothing will happen when the CaF_2:Tb^{3+}/PDMS elastomer is held in the stretched position.

Supplementary Note 12: Relatively electronegativity of the phosphors and PDMS

Generally, each and every material exhibits triboelectrification. To standardize its quantification, the triboelectric series for a wide range of polymers have been quantified by Prof. Z.L. Wang's group[8]. According to the triboelectric series, the PDMS is relatively electronegative compared to most polymers, but it may be not the case for the phosphor-PDMS couple. Supplementary Figure 70 presents the surface electron distribution of the new CaF_2:Tb^{3+}/PDMS elastomer, and it shows that the sprayed electrons are mainly concentrated on the phosphor particles. This result experimentally demonstrates that the phosphor particles attract electrons more easily than the PDMS polymer.

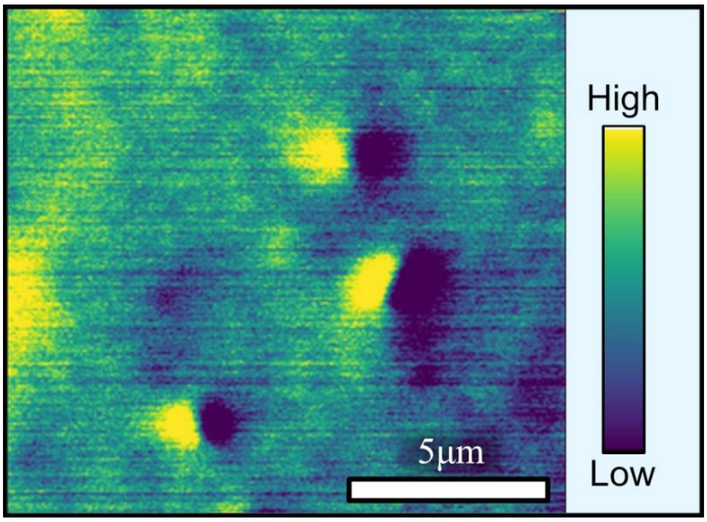

Supplementary Figure 70. Surface electron distribution of the CaF_2:Tb^{3+}/PDMS elastomer.

At this stage, we can only give a possible explanation for the relatively electronegativity of the phosphors. Generally, the ability of a substance to attract electrons is essentially related to the elemental composition and structural characteristics of the substance itself[9]. For PDMS, it is relatively electronegative in the polymers. However, its main constituent is carbon with relatively low electronegativity. Additionally, PDMS does not contain electron-withdrawing groups such as benzene rings (C_6H_6), halogen atoms (e.g., fluorine, chlorine, bromine), or nitro groups ($-NO_2$)

that strongly attract electrons[10]. On the contrary, inorganic phosphors usually contain non-metallic elements with higher electronegativities, such as oxygen, nitrogen, and fluorine, which strongly attract surrounding electrons[11]. Furthermore, metallic elements in inorganic materials often have low electronegativities but can lose electrons to form metal cations, which possess strong electron-attracting capabilities. In my opinion, the inorganic $CaF_2:Tb^{3+}$ phosphors containing highly electronegative fluorine and Ca^{2+} cations should be more electronegative than the organic PDMS polymers.

Supplementary Note 13: The role of friction on the triboelectrification

It is well known that the concept of the contact electrification was proposed by Prof. Zhonglin Wang and has been introduced in detail in many important references[12–15]. At present, it has been widely accepted that the charges are produced due to physical contact, and mechanical friction is not necessary although it can aid in delivering the charges. (The original text of this statement appears on page 35 of the reference 3 listed below.) To help broader readers better understand the contact-separation-induced self-recoverable ML mechanism of this new $CaF_2:Tb^{3+}$/PDMS elastomer, it is necessary to make an explicit introduction to the contact-separation process based on contact electrification.

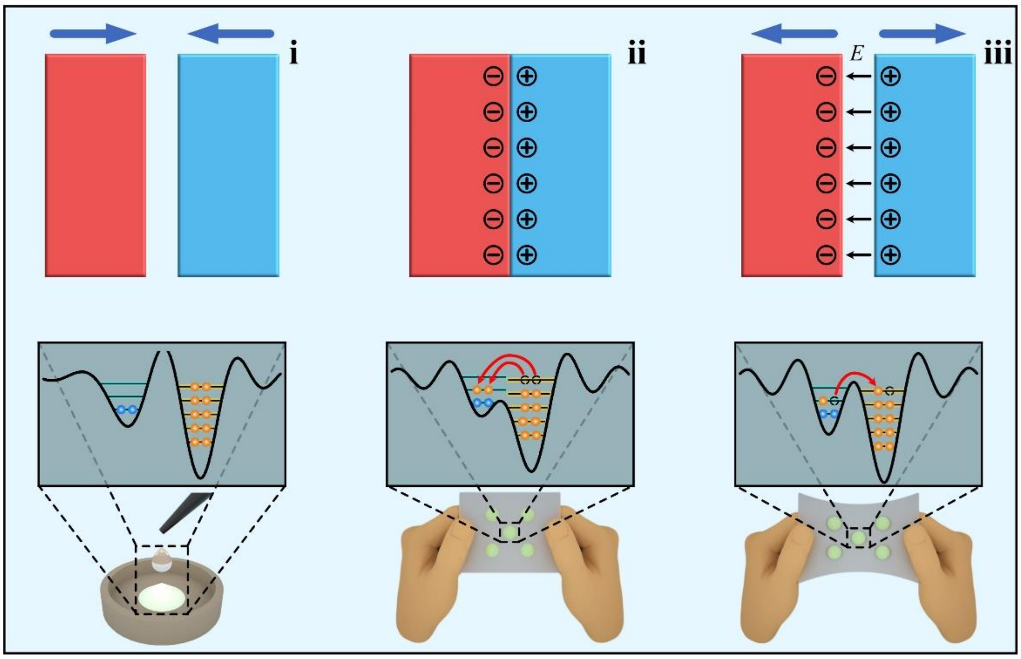

Supplementary Figure 71. Formation and recombination mechanism of the electron-hole pairs for the $CaF_2:Tb^{3+}$/PDMS elastomer

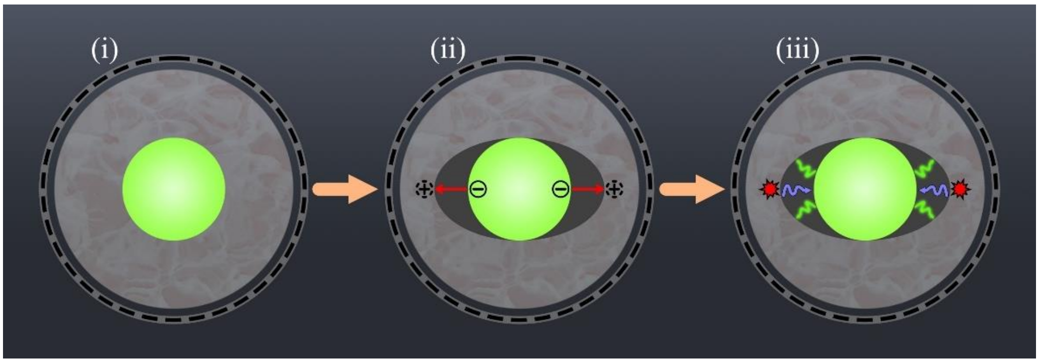

Supplementary Figure 72. Contact-separation-induced ML mechanism of the new CaF_2:Tb^{3+}/PDMS elastomer.

Generally, contact electrification is the scientific term for triboelectrification. The formation and recombination mechanism of the electron-hole pairs for the CaF_2:Tb^{3+}/PDMS elastomer can be depicted in Supplementary Figure 71. First (i), before being compounded, the potential wells of the phosphors and PDMS are separated, and no electrostatic charges are created on their surfaces. However, owing to the different energy bands of each material, the energy of the occupied surface states of the PDMS is higher than that of the unoccupied surface states of the phosphors. Second (ii), when the phosphors and PDMS are in close contact after being compounded, their atomic electron clouds overlap to form covalent bonds. The two single potential wells become an asymmetric double potential well, and the energy barrier between the two wells is lowered due to the strong electron-cloud overlap. Consequently, the electrons at higher levels transfer from PDMS to phosphors to maintain energy level balance even if there is no friction between the phosphor and PDMS. At this stage, no electrostatic charges are created due to the formation of new energy level balance of the asymmetric double potential well. Third (iii), when the elastomer is stretched, the previously contacted surfaces of the phosphors and PDMS is slightly separated. However, the electrons still remain on the surface of phosphors, resulting in negative electrostatic charges (electrons) on the phosphors and positive electrostatic charges (holes) on the PDMS due to contact electrification effect[12–15]. At the same time, the electron-hole pairs generate a strong electrostatic field. Because the gaps between the phosphors and the PDMS are very tiny, the distance between the electron and hole is actually very short as well. Therefore, the negative electrons on the surface of the phosphors can be

attracted back to the PDMS by the opposite charge (positive hole) in a short time. Consequently, the electron-hole recombination occurs at the phosphor-PDMS interfaces to release sufficient excitation energy, thereby exciting the nearby Tb^{3+} emitters for ML. In our lives, this electron-hole recombination generally occurs to induce some interesting static electricity phenomena, such as static sparks and beeping noise. For example, we may see the bright static sparks when we take off a sweater on a dry winter night. According to the above results and discussions, the contact-separation-induced ML mechanism of the new CaF_2:Tb^{3+}/PDMS elastomer can be depicted in Supplementary Figure 72.

Supplementary Note 14: Humidity and other factors affecting ML intensity

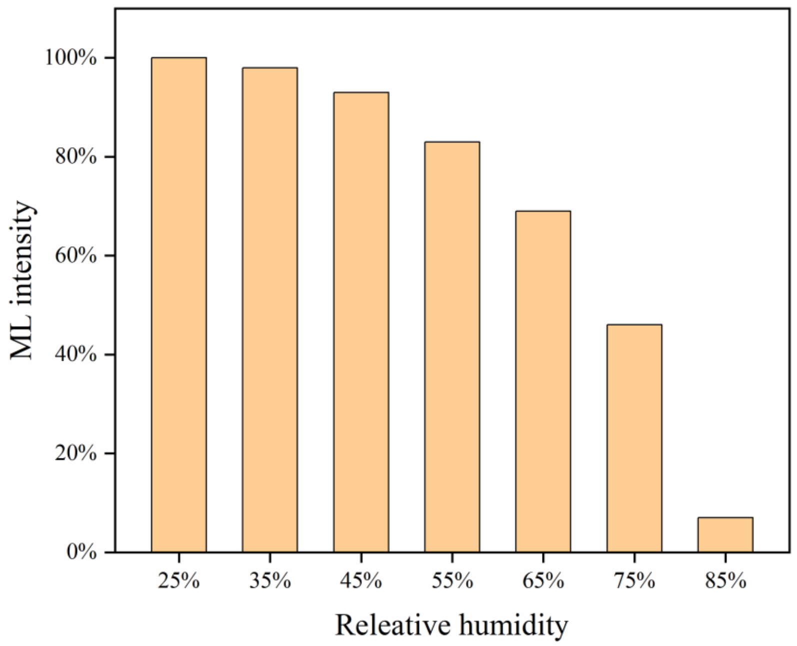

Supplementary Figure 73. ML intensities of the elastomer dependent on relative humidity.

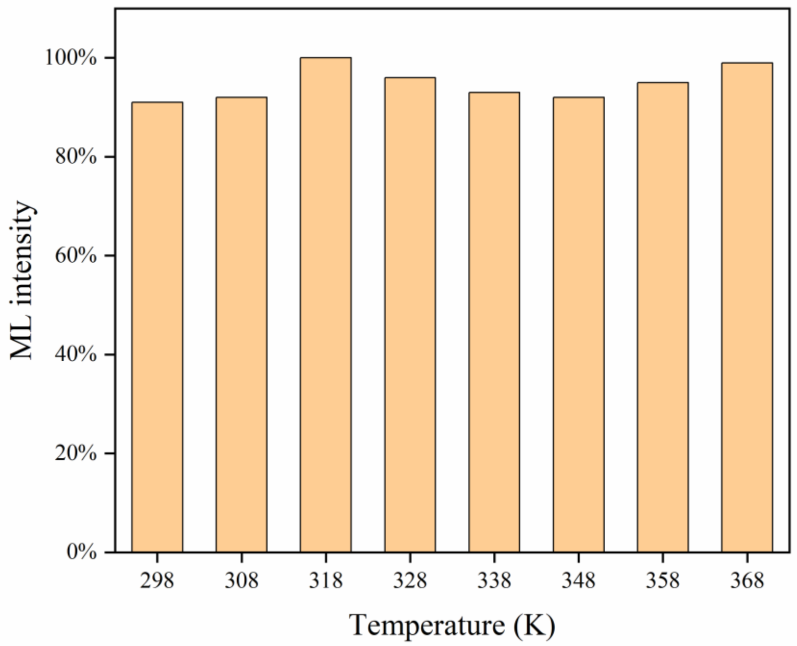

Supplementary Figure 74. ML intensities of the elastomer dependent on temperature.

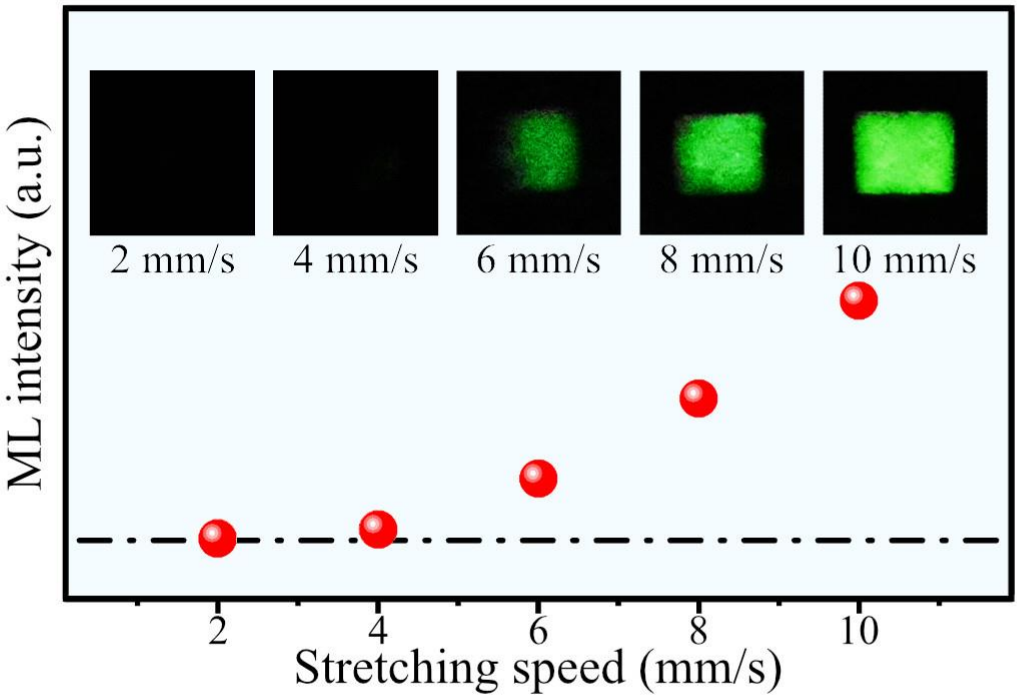

Supplementary Figure 75. ML intensity of CaF_2:Tb^{3+}/PDMS dependent on stretching speeds

The Supplementary Figure 75 reveals a positive correlation between ML intensity and stretching speed, with greater ML intensity observed at higher stretching speeds. This can be attributed to the higher pulling force required for faster stretching speeds, which increases the probability of phosphor particles separation from the PDMS.

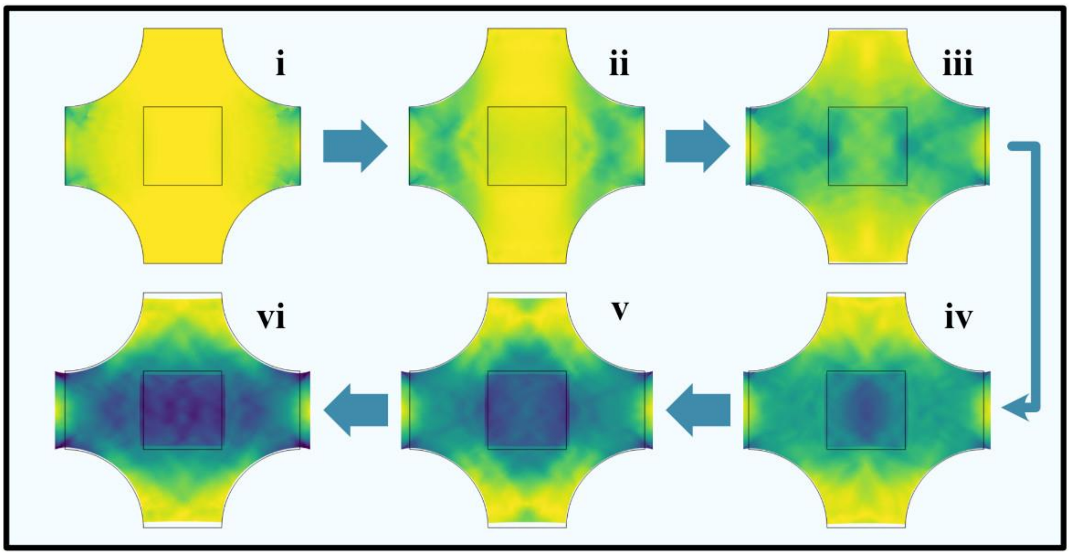

Supplementary Figure 76. Transient stress distribution during stretching

Supplementary Figure 76 displays the transient stress distribution of the sample during stretching, while Supplementary Figure 77 depicts the stress curves of the composite center at various tensile speeds. The stress propagates in waves during stretching, and when the stretching speed is too slow (<6 mm/s), the stress is insufficient to excite ML, which agrees with the experimental findings.

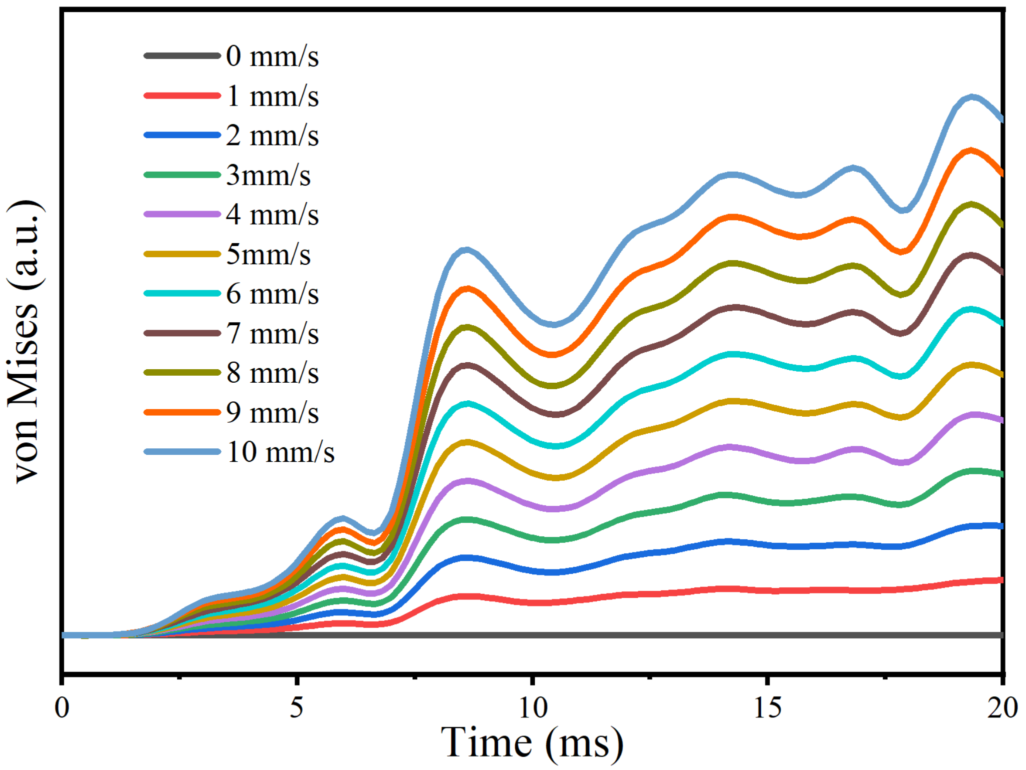

Supplementary Figure 77. Stress curve at the center of the elastomer dependent on stretching speeds

Supplementary Note 15: More explanation on the scratching-induced separation

Generally, we see nothing doesn't mean nothing happened. In this case, although it seems that we cannot “see” the scratching-induced separation between the $CaF_2:Tb^{3+}$ phosphors and PDMS with our naked eyes, it does occur.

Supplementary Table 18. Stretching stresses and scratching stresses to the $CaF_2:Tb^{3+}$/PDMS elastomer under different pressures.

Pressure (MPa)	Stretching stress (N)	Scratching stress (N)
0.1261	1.3778	0.0634
0.2341	2.5571	0.1176
0.3304	3.6101	0.1660
0.4215	4.6051	0.2118
0.5074	5.5434	0.2549
0.5926	6.4743	0.2977
0.6798	7.4264	0.3415
0.7729	8.4442	0.3883
0.8799	9.6127	0.4421
1.0013	10.9390	0.5030

In this case, the entire elastomer is stressed under stretching, while only a very tiny area in contact with the nail is stressed under scratching. Therefore, the stress-bearing area of the elastomer under stretching is much larger than that under scratching. Consequently, even if the same pressure is applied, the stress under stretching must be much greater than the stress under scratching due to the much larger stress-bearing area. Accordingly, igure 18 shows the stretching stresses and scratching stresses to the $CaF_2:Tb^{3+}$/PDMS elastomer under different pressures. For example, it shows that when the pressure applied to the elastomer is 0.2341 MPa, the stretching stress (2.5571 N) is even more than 20 times the scratching stress (0.1176 N). Consequently, we always feel that the stress under scratching is very “slight” and the stress under stretching is very “hard”, but in fact the pressure on the elastomer is still the same for both models. In this case, we may think we don't “see” the separation because we feel the stress under scratching is very “slight”, but in fact this is an illusion. As mentioned in the above example, although the scratching stress applied to the $CaF_2:Tb^{3+}$/PDMS elastomer is as

small as 0.117592 N, the local pressure on the elastomer is still as high as 0.23406 MPa, which is sufficient to induce strong strain and tiny interface separation between the phosphor particles and PDMS.

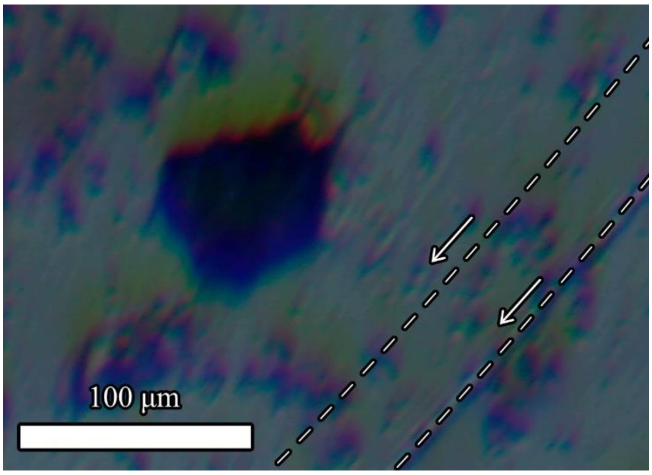

Supplementary Figure 78. Microscope image of the CaF_2:Tb^{3+}/PDMS elastomer after scratching 200 times.

As evidences, Supplementary Figure 78 shows the microscope image of the CaF_2:Tb^{3+}/PDMS elastomer after scratching 200 times. It shows that the edge area of the phosphor particles in the elastomer is clearly dyed red corresponding to the tiny gaps (separation) at the interface between the phosphors and PDMS. Furthermore, Supplementary Figure 79 depicts that the scratching-induced strain in the elastomer and the tiny gaps (separation) between the phosphor particles and PDMS under scratching based on finite element calculation. It presents that during the scratch, the scratching-induced strain (green region) of the elastomer can be clearly observed, resulting in the nearby separation (white region) between the phosphor particles and PDMS. However, the separation under scratching is clearly slighter, and therefore the scratching-induced damages are also slighter, corresponding to a weaker ML and a more stable ML under continuous scratching.

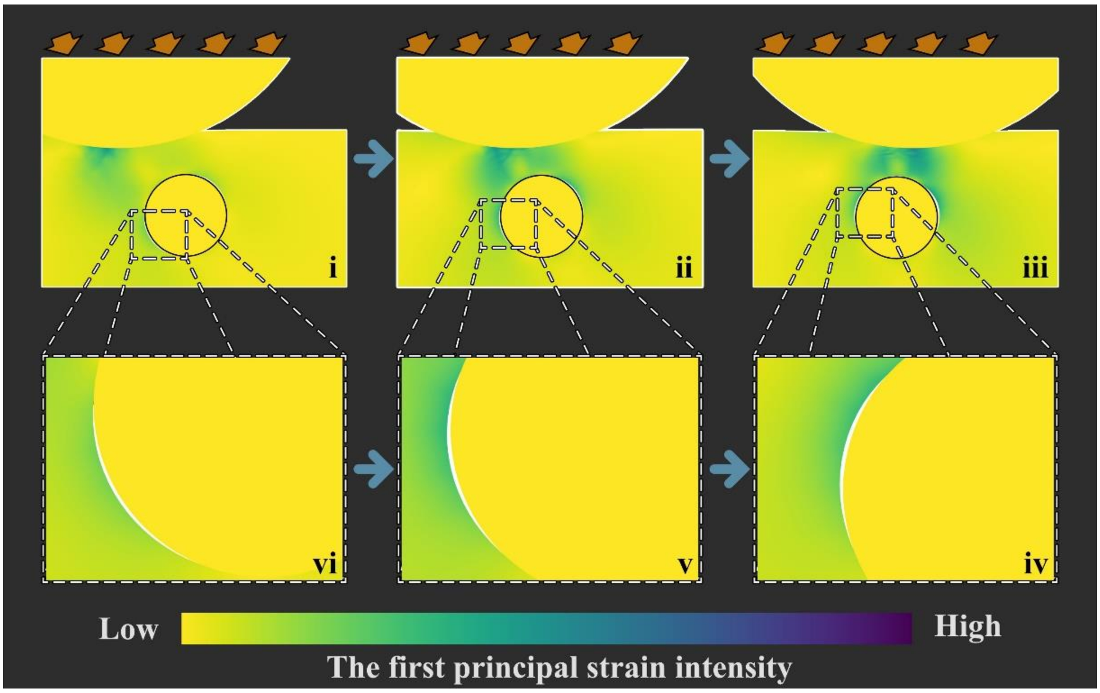

Supplementary Figure 79. Strain of the elastomer and the separation between the phosphors and PDMS under scratching based on finite element calculation.

Supplementary Note 16: First-principles calculation of contact-separation

Calculation Method：First-principles calculations are performed by vienna ab initio simulation package (VASP)[16–20]. The generalized gradient approximation(GGA) of Perdew-Burke-Ernzerhof (PBE) is used to describe the exchange-correlation functional. The cut-off energy for the plane wave basis is set to 400 eV and a 2×2×1 mesh is employed. All the structures were fully relaxed (atomic position) up to 10^{-5} eV /Å force minimization and max force of 0.01 eV/Å.

All transition states were calculated using the Transition State Tools for VASP-VTST method, with the stretching frequencies analyzed in order to characterize whether a stationary point is a minimum state without imaginary frequency or a transition state with only one imaginary frequency. For the transition state structures, frequency analysis is carried out, and zero point energy correction is carried out for all the energy obtained to eliminate the impact of vibration on the energy.

The reaction energy are defined as:

$$\Delta E_{21} = E_2 - E_1$$

$$\Delta E_{31} = E_3 - E_1$$

$$E_{23} = E_2 - E_3$$

Calculation parameters summary:

Cutoff energy: 400 eV

Electronic self-consistent convergence criterion: 10^{-5} eV/Å

Atomic relaxation convergence criterion: -0.01 eV/Å

K-point grid: 2×2×1

Functional: PAW-PBE

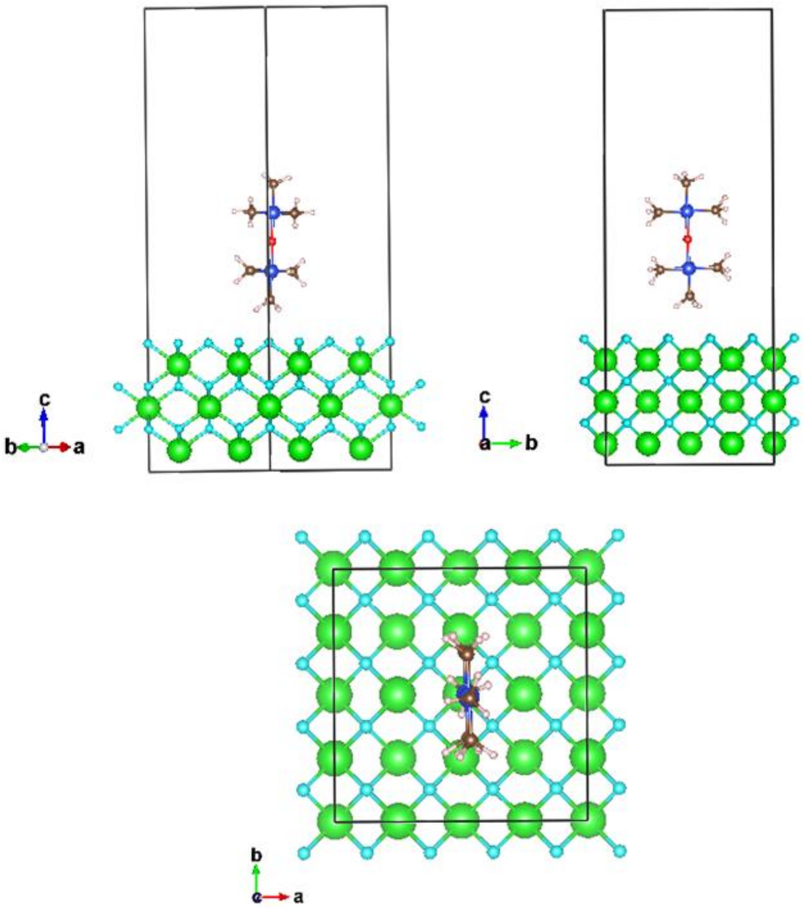

Supplementary Figure 80. Initial state of CaF_2/PDMS elastomer

E_1 = -493.27614475 eV

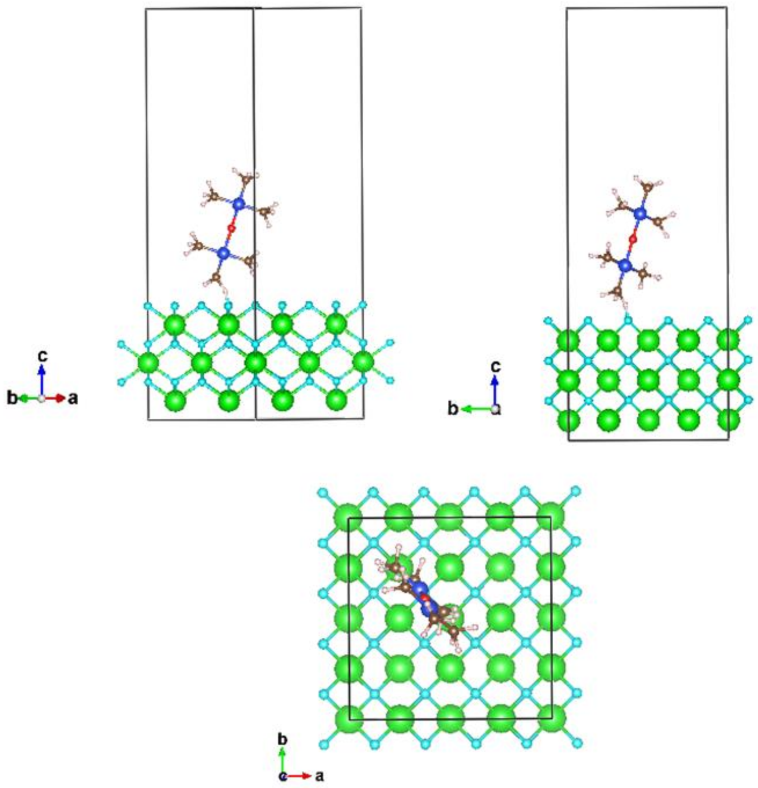

Supplementary Figure 81. Contact state of CaF_2/PDMS elastomer

E_2 = -492.89820746 eV

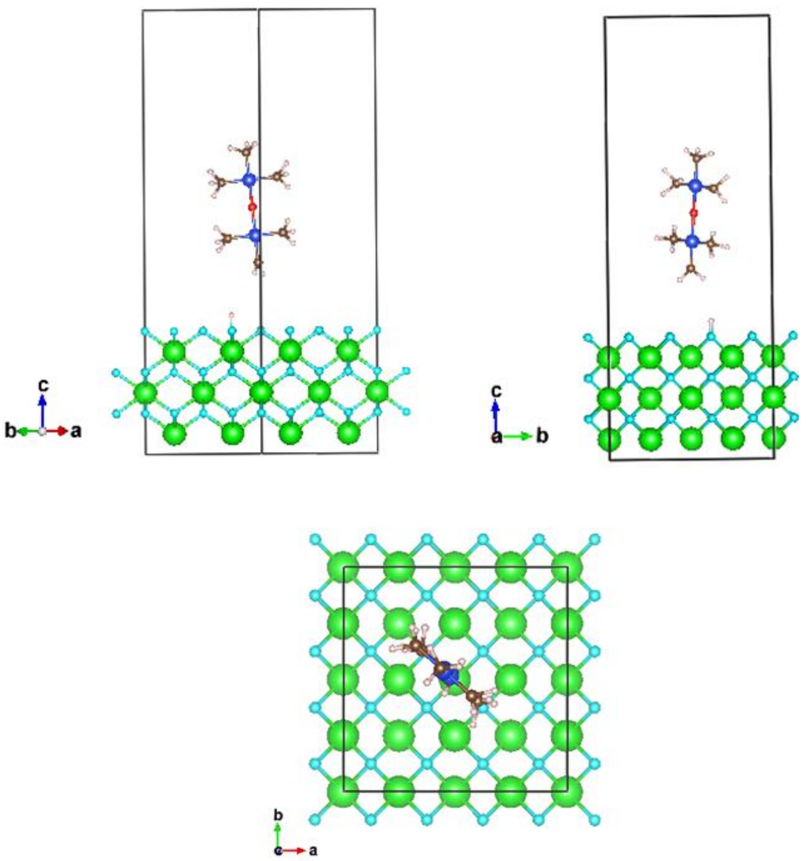

Supplementary Figure 82. Separation state of CaF_2/PDMS elastomer

E_3 = -494.13918038 eV

Calculated to get:

$$\Delta E_{21} = E_2 - E_1 = -492.89820746 - -493.27614475 = 0.378 \text{ eV}$$
$$\Delta E_{31} = E_3 - E_1 = -494.13918038 - -493.27614475 = -0.863 \text{ eV}$$
$$\Delta E_{23} = E_2 - E_3 = -492.89820746 - -494.13918038 = 1.241 \text{ eV}$$

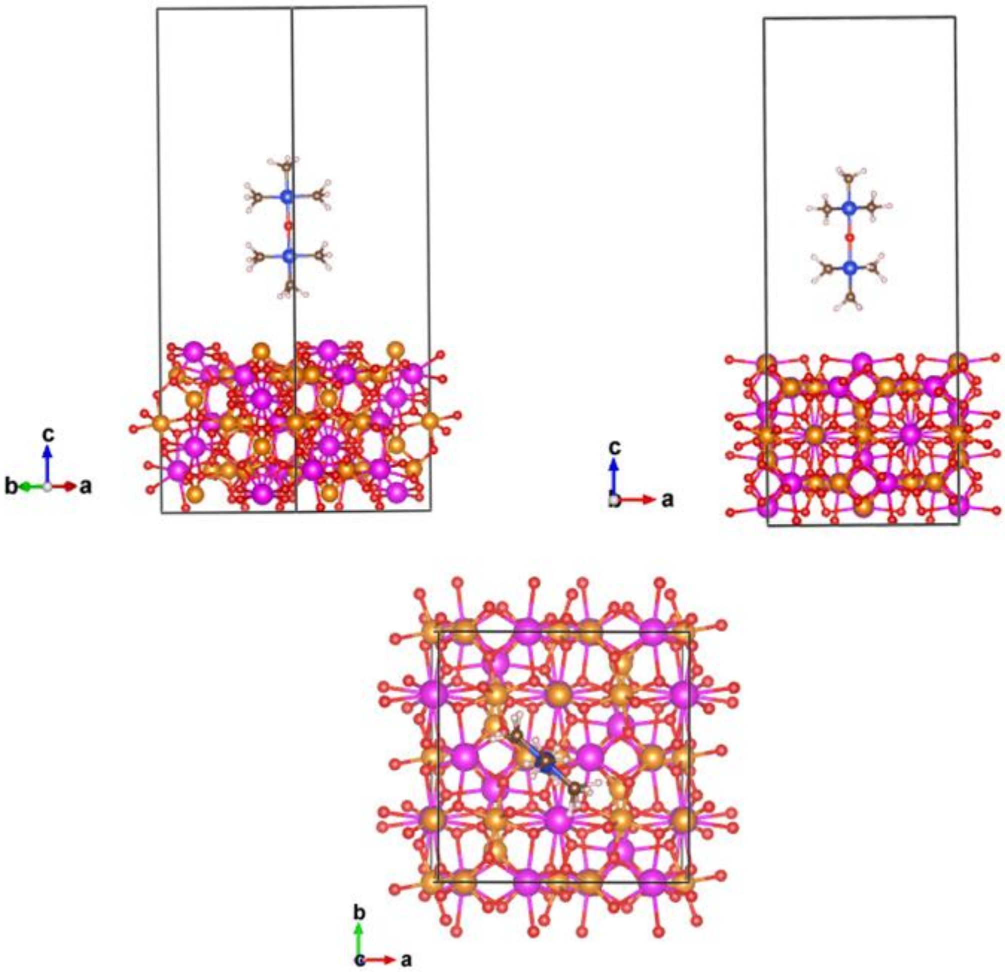

Supplementary Figure 83. Initial state of $Lu_3Al_5O_{12}$/PDMS elastomer

E_1 = -1122.80316728 eV

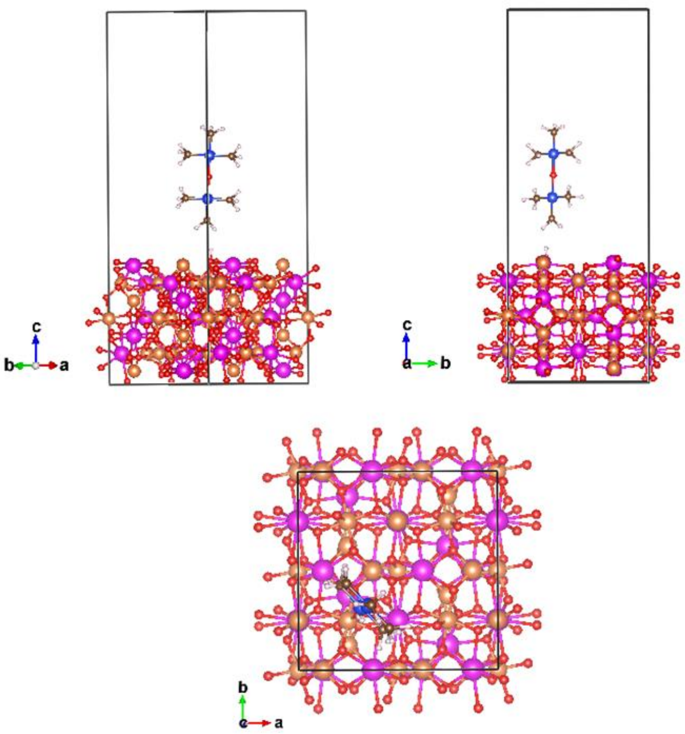

Supplementary Figure 84. Contact state of $Lu_3Al_5O_{12}$/PDMS elastomer

E_2 = -1121.72060209 eV

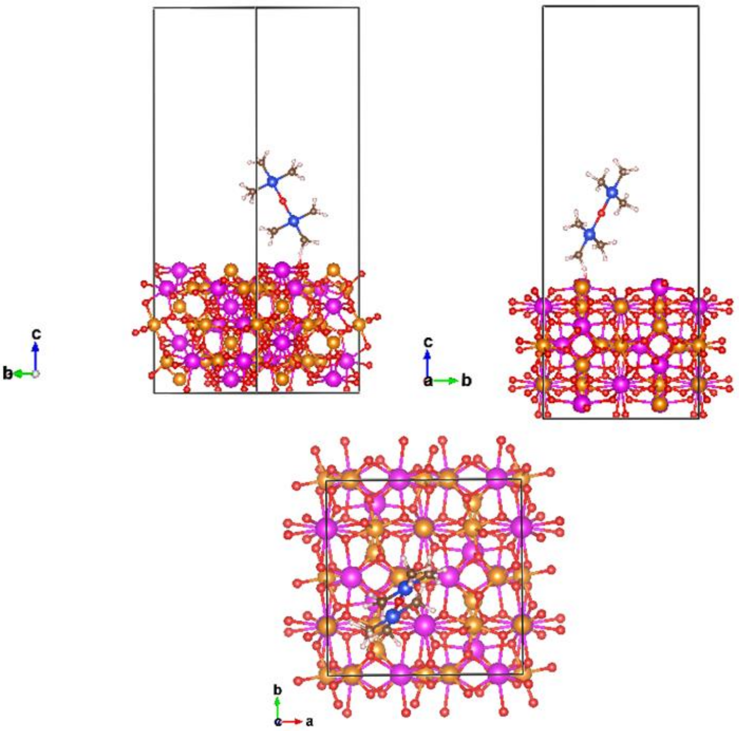

Supplementary Figure 85. Separation state of $Lu_3Al_5O_{12}$/PDMS elastomer

E_3 = -1123.97150073 eV

Calculated to get:

$$\Delta E_{21} = E_2 - E_1 = -1121.72060209 - -1122.80316728 = 1.083 \text{ eV}$$

$$\Delta E_{31} = E_3 - E_1 = -1123.97150073 - -1122.80316728 = -1.168 \text{ eV}$$

$$\Delta E_{23} = E_2 - E_3 = -1121.72060209 - -1123.97150073 = 2.251 \text{ eV}$$

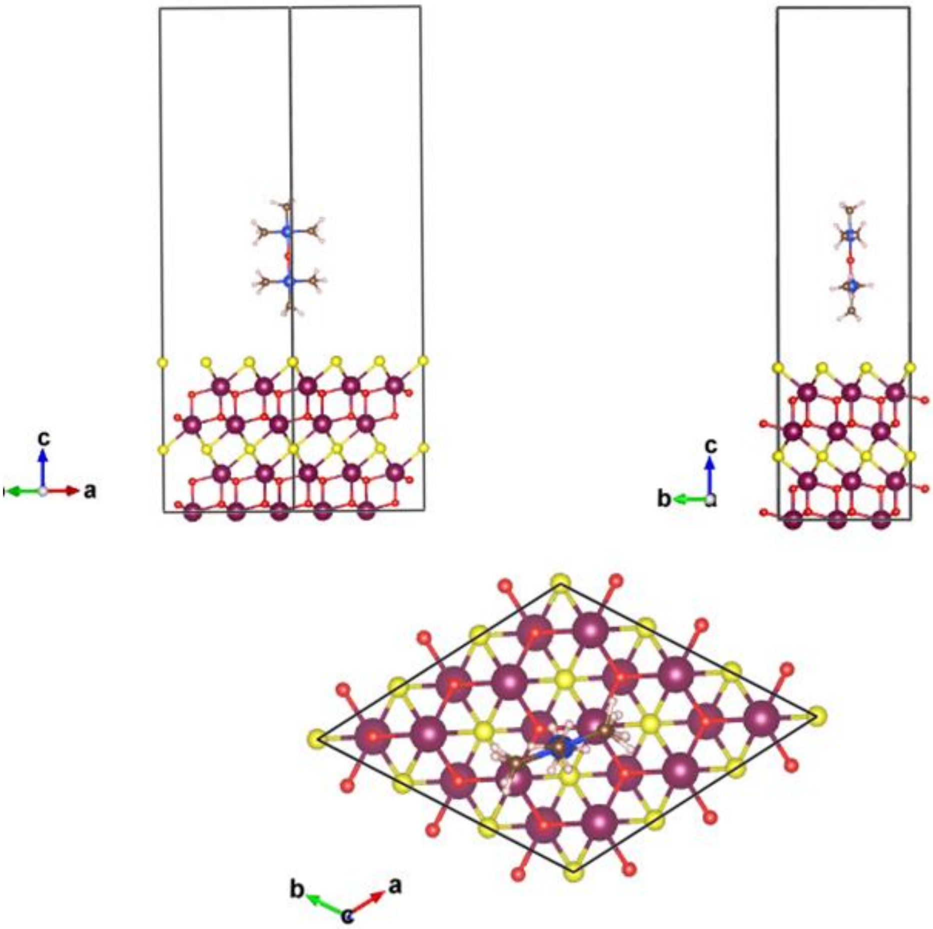

Supplementary Figure 86. Initial state of Y_2O_2S/PDMS elastomer

E_1 = -860.42780451 eV

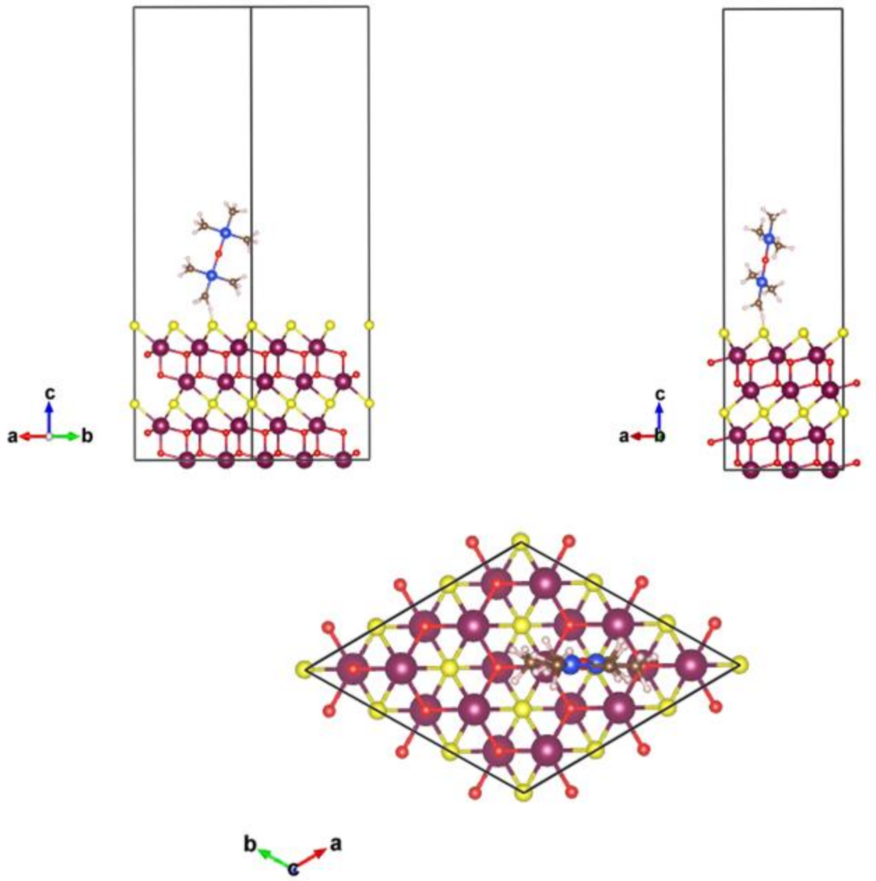

Supplementary Figure 87. Contact state of Y_2O_2S/PDMS elastomer

E_2 = -858.92551482 eV

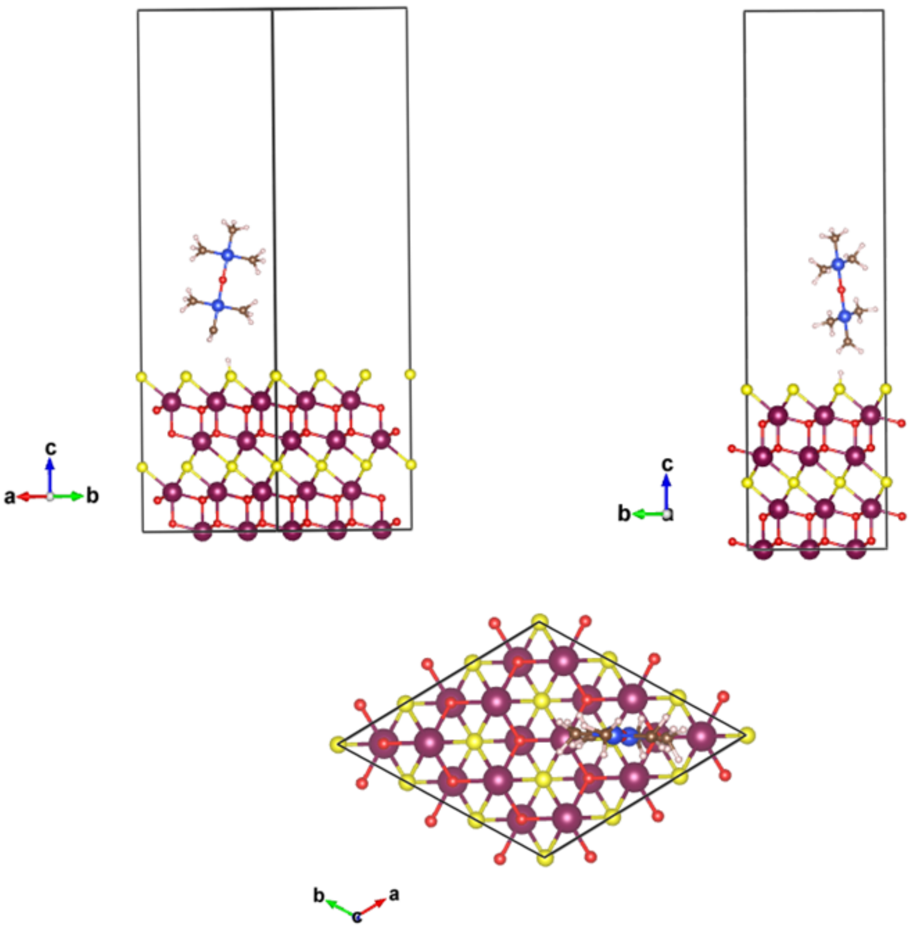

Supplementary Figure 88. Separation state of Y_2O_2S/PDMS elastomer

$$E_3 = -861.16371424 \text{ eV}$$

Calculated to get:

$$\Delta E_{21} = E_2 - E_1 = -858.92551482 - -860.42780451 = 1.502 \text{ eV}$$

$$\Delta E_{31} = E_3 - E_1 = -861.16371424 - -860.42780451 = -0.736 \text{ eV}$$

$$\Delta E_{23} = E_2 - E_3 = -858.92551482 - -861.16371424 = 2.238 \text{ eV}$$

Supplementary Note 17: Additional first-principles calculations

Calculation Method： First-principles calculations are performed by vienna ab initio simulation package (VASP)[16–20]. The generalized gradient approximation(GGA) of Perdew-Burke-Ernzerhof (PBE) is used to describe the exchange-correlation functional. The cut-off energy for the plane wave basis is set to 400 eV and a 2×2×2 Monkhorst-pack mesh is employed. All the structures were fully relaxed (atomic position) up to 10-5 eV /Å force minimization and max force of 0.01 eV/ Å.

Molecular dynamics methodology: Each polymer chain consists of 14 DMS monomers arbitrarily cross-linked to represent a simplified photo-polymerization process. The choice of such a short chain with a consistent degree of polymerization is in accordance with many polymer composite models employed in MD simulations to improve computational efficiency whilst maintain essential polymer features such as contact resistance, glass transition behavior, and thermo-mechanical properties, etc.

And the nanorods were homogenously distributed in the resin matrix due to the periodic boundary conditions applied in the three dimensions.

Calculation parameters summary:
Cutoff energy: 400 eV
Electronic self-consistent convergence criterion: 10^{-5} eV/Å
Atomic relaxation convergence criterion: -0.01 eV/Å
K-point grid: 2×2×1
Functional: PAW-PBE

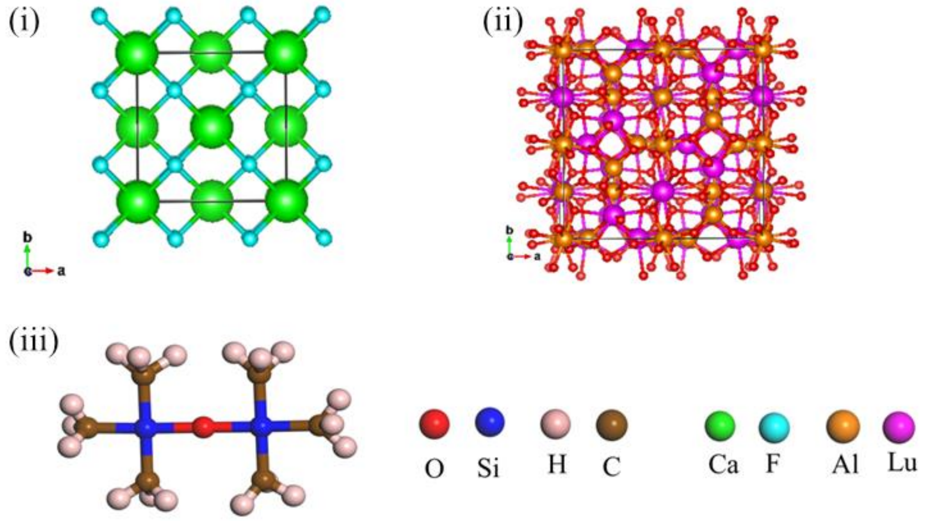

Supplementary Figure 89. Structures used in calculations

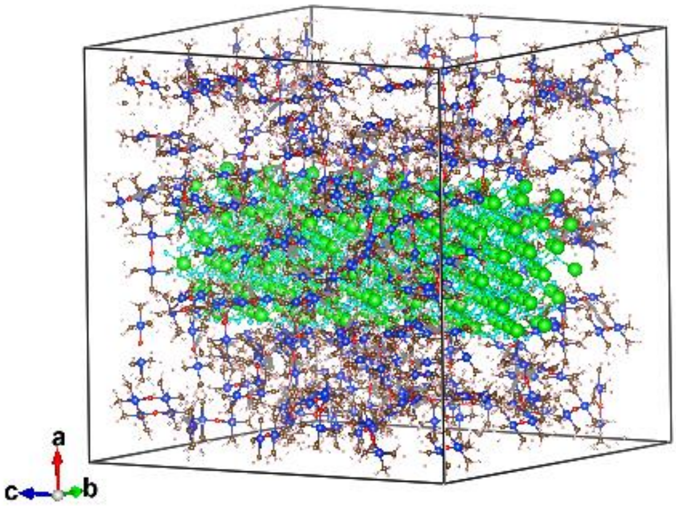

a=4.485 nm, b=4.485 nm, c= 4.55 nm

Supplementary Figure 90. CaF_2/PDMS composites with a mass ratios of 10.25%

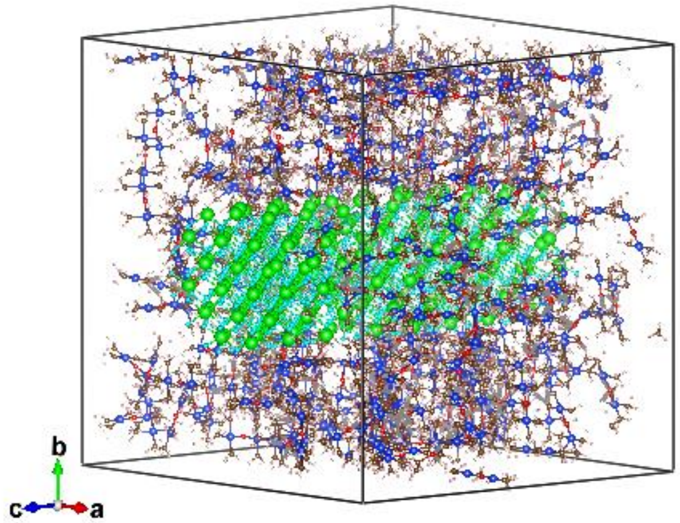

a=4.773 nm, b=4.773 nm, c= 4.55 nm

Supplementary Figure 91. CaF_2/PDMS composites with a mass ratios of 13.73%

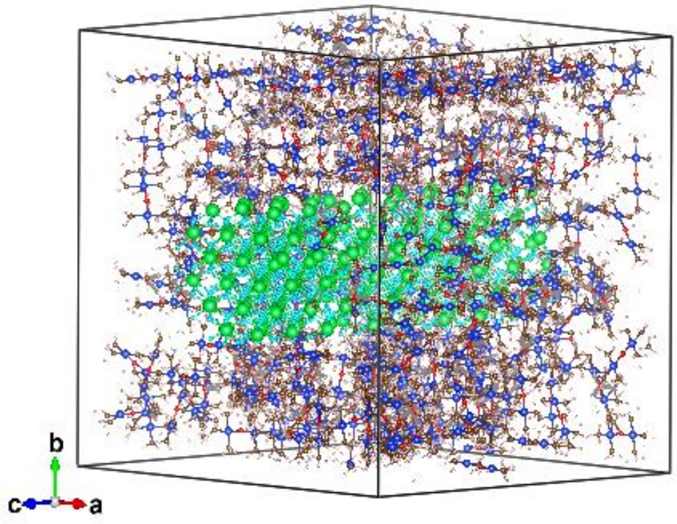

a=4.972 nm, b=4.972 nm, c= 4.55 nm

Supplementary Figure 92. CaF_2/PDMS composites with a mass ratios of 16.64%

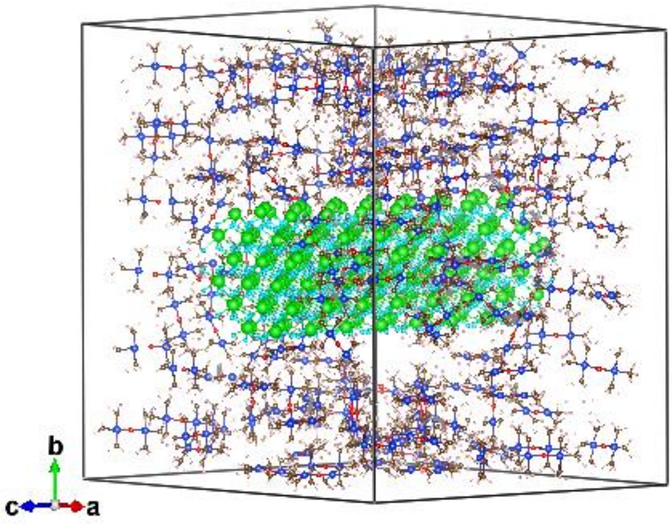

a=5.462 nm, b=5.462 nm, c= 4.55 nm

Supplementary Figure 93. CaF_2/PDMS composites with a mass ratios of 20.55%

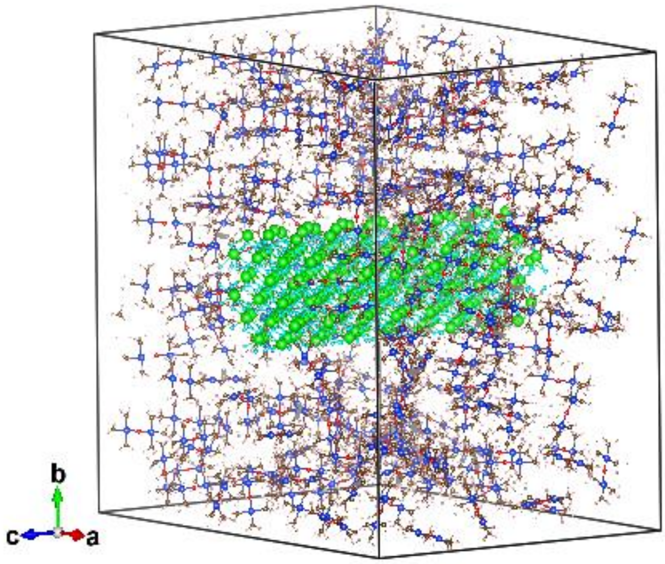

a=6.342 nm, b=6.342 nm, c= 4.55 nm

Supplementary Figure 94. CaF_2/PDMS composites with a mass ratios of 22.57%

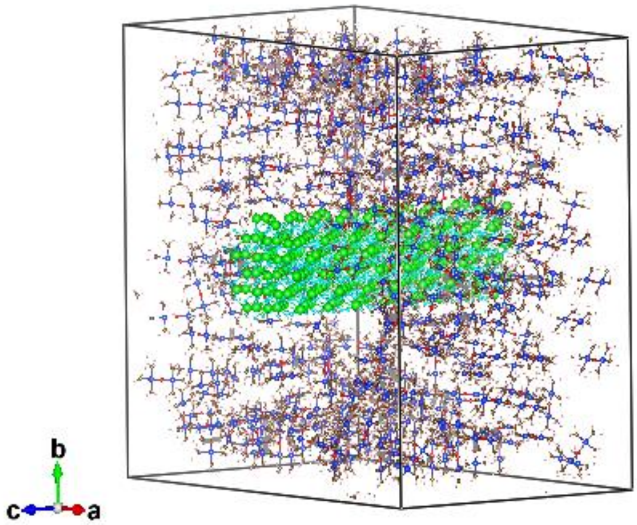

a=7.154 nm, b=7.154 nm, c= 4.55 nm

Supplementary Figure 95. CaF_2/PDMS composites with a mass ratios of 25.39%

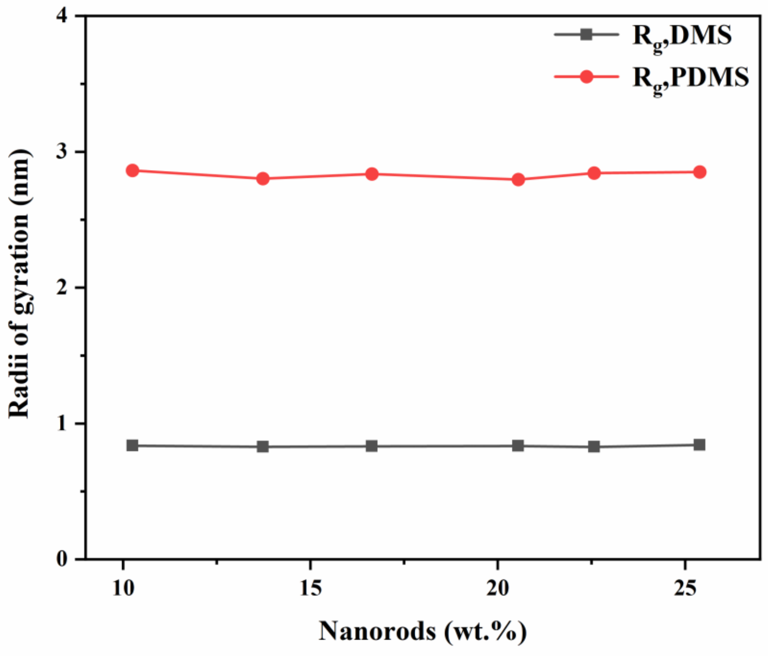

Supplementary Figure 96. Radius of gyration of CaF_2/PDMS composites dependent on mass ratios

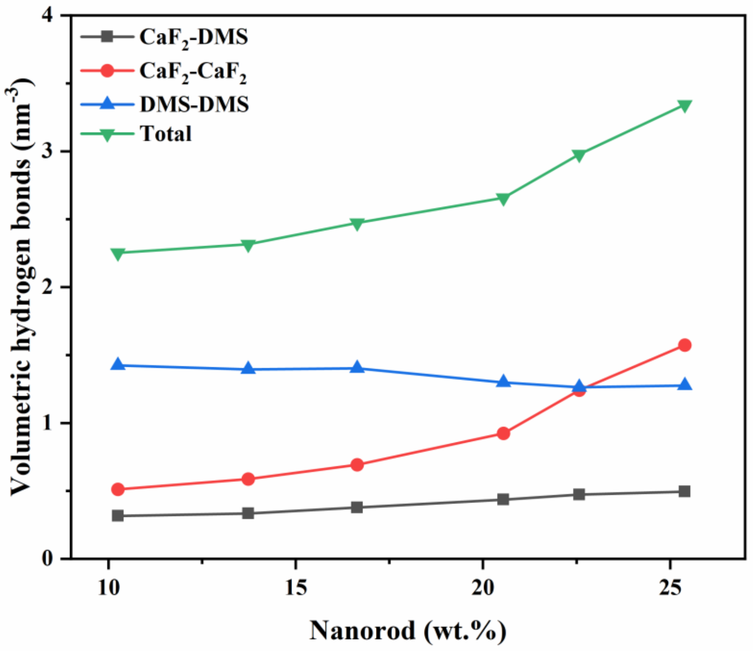

Supplementary Figure 97. Volumetric hydrogen bonds of CaF_2/PDMS composites dependent on mass ratios

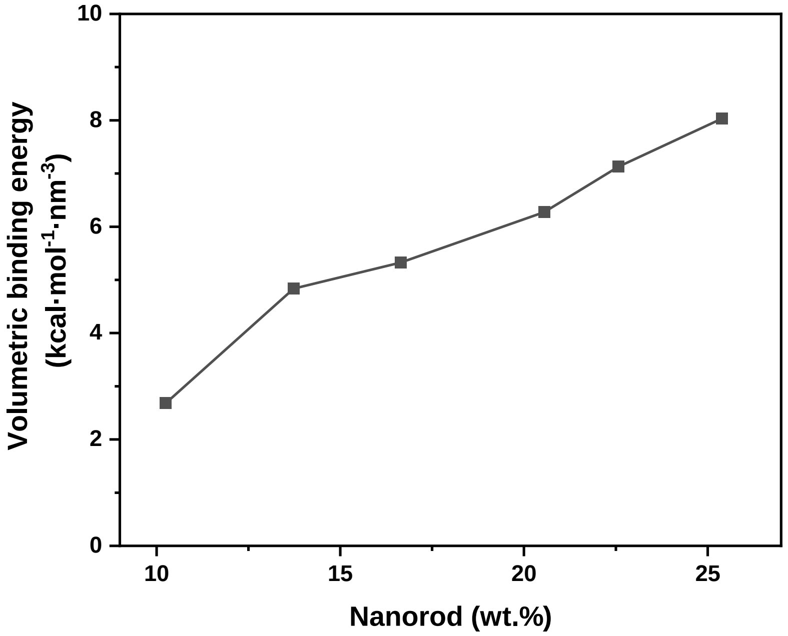

Supplementary Figure 98. Volumetric binding energy of CaF_2/PDMS composites dependent on mass ratios

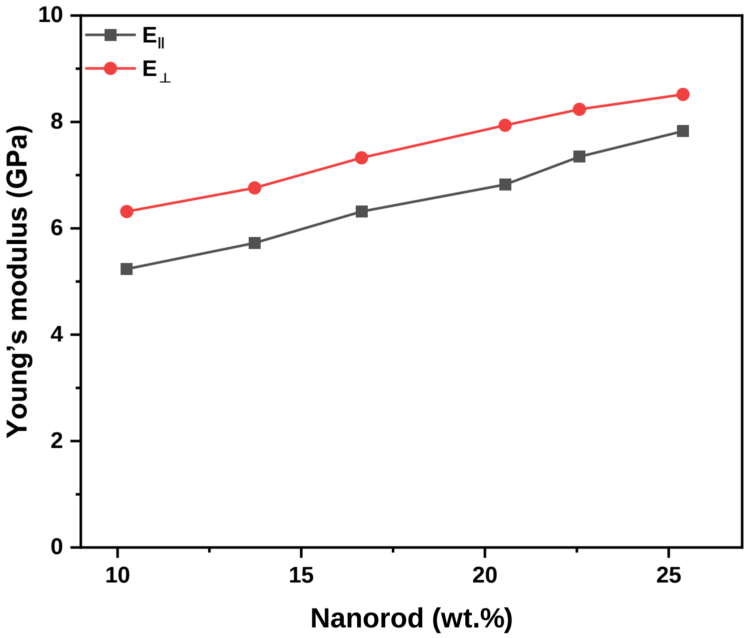

Supplementary Figure 99. Young's modulus of CaF_2/PDMS composites dependent on mass ratios

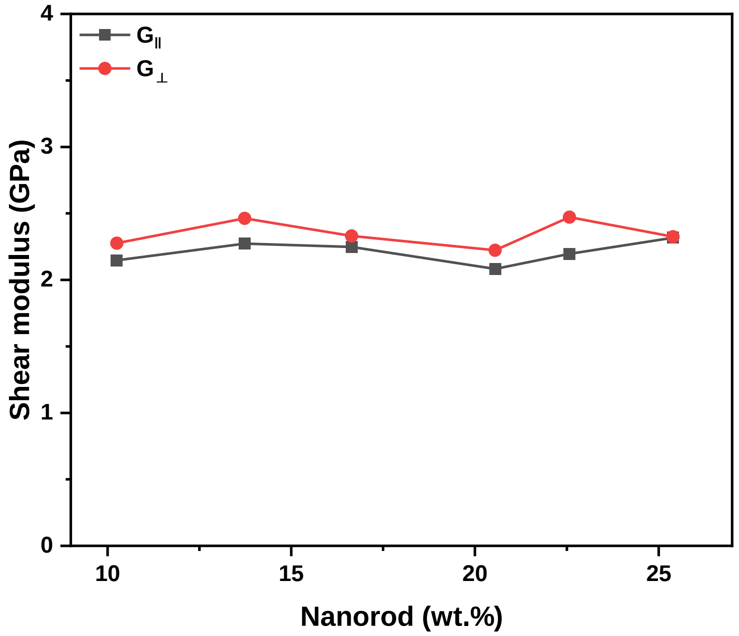

Supplementary Figure 100. Shear modulus of CaF_2/PDMS composites dependent on mass ratios

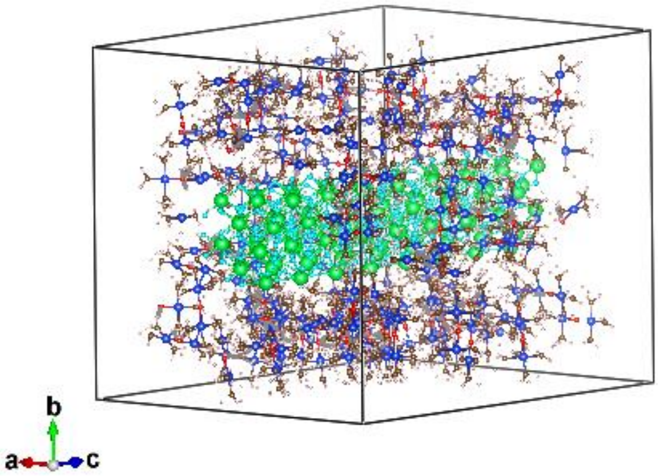

a=3.657 nm、b=3.657 nm、c= 4.55 nm

Supplementary Figure 101. CaF_2/PDMS composites with a diameter of 1 nm

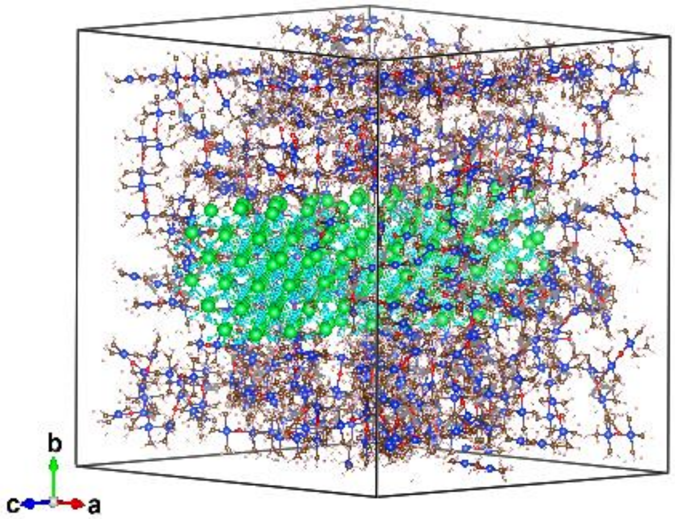

a=4.972 nm、b=4.972 nm、c= 4.55 nm

Supplementary Figure 102. CaF_2/PDMS composites with a diameter of 1.5 nm

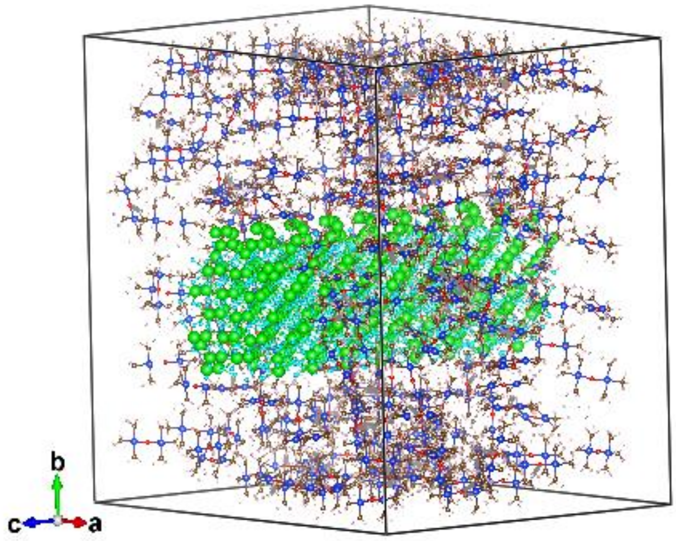

a=5.872 nm、b=5.872 nm、c= 4.55 nm

Supplementary Figure 103. CaF_2/PDMS composites with a diameter of 2 nm

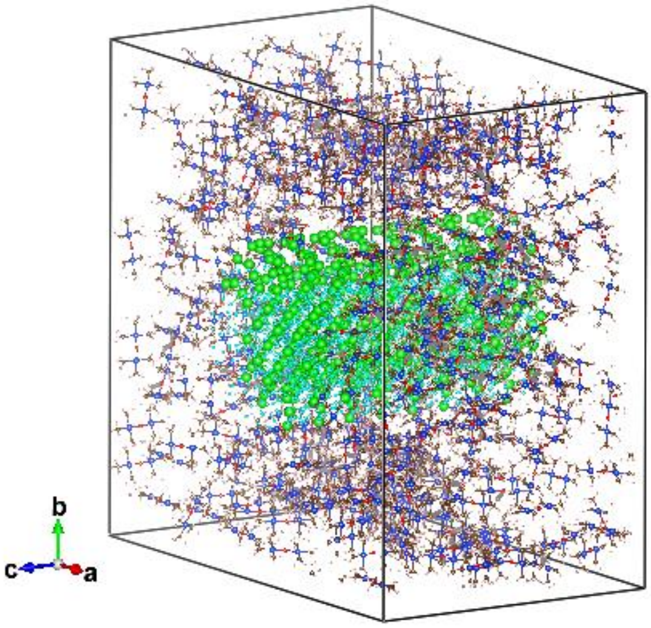

a=7.416 nm、b=7.416 nm、c= 4.55 nm

Supplementary Figure 104. CaF_2/PDMS composites with a diameter of 2.5 nm

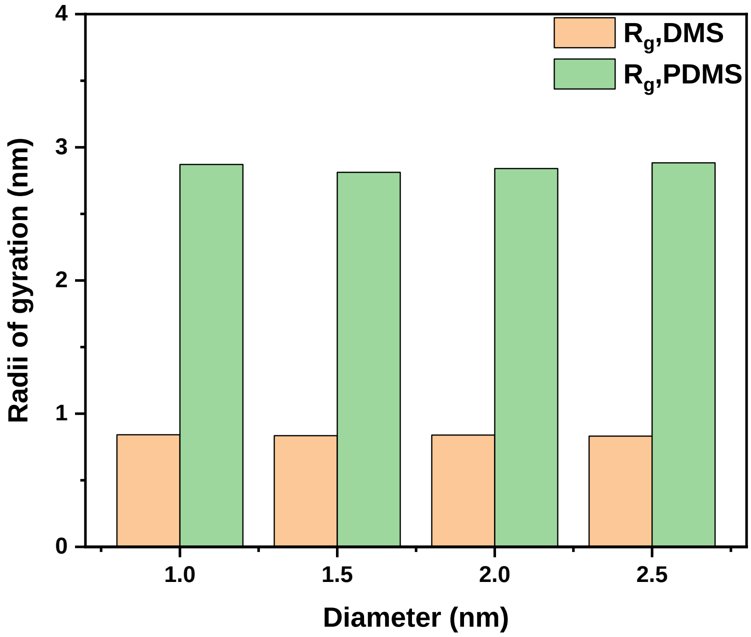

Supplementary Figure 105. Radius of gyration of CaF_2/PDMS composites dependent on diameter

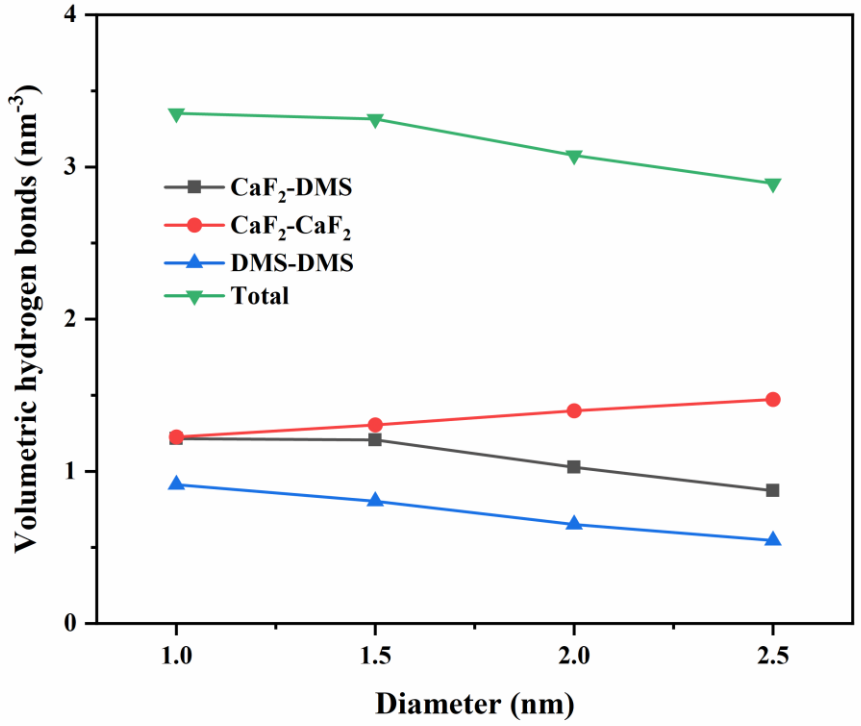

Supplementary Figure 106. Volumetric hydrogen bonds of CaF_2/PDMS composites dependent on diameter

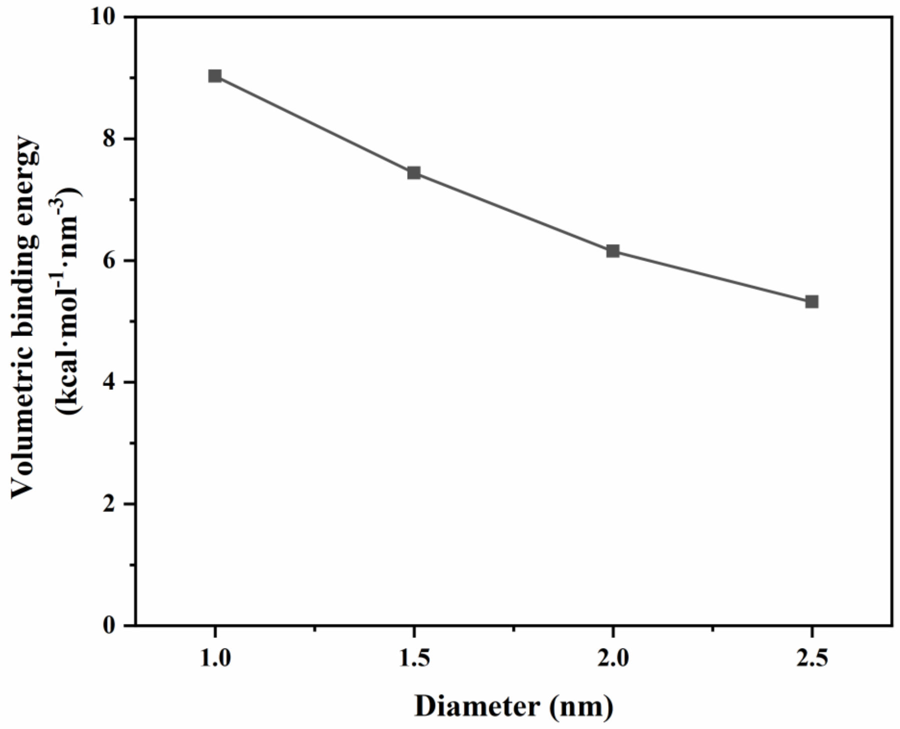

Supplementary Figure 107. Volumetric binding energy of CaF_2/PDMS composites dependent on diameter

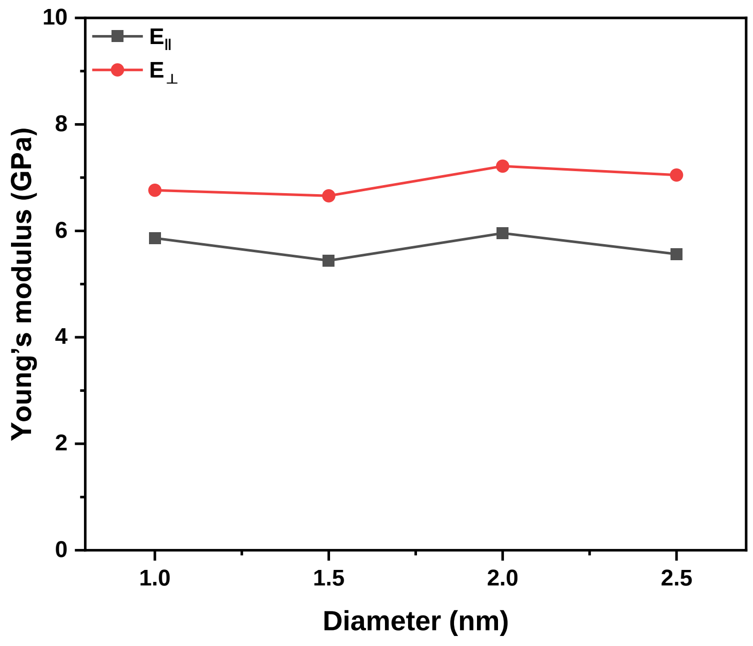

Supplementary Figure 108. Young's modulus of CaF_2/PDMS composites dependent on diameter

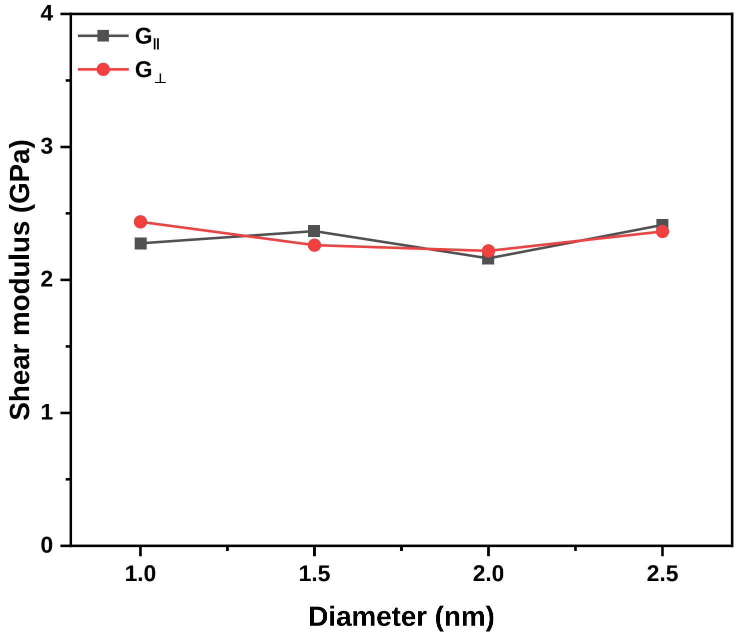

Supplementary Figure 109. Shear modulus of CaF_2/PDMS composites dependent on diameter

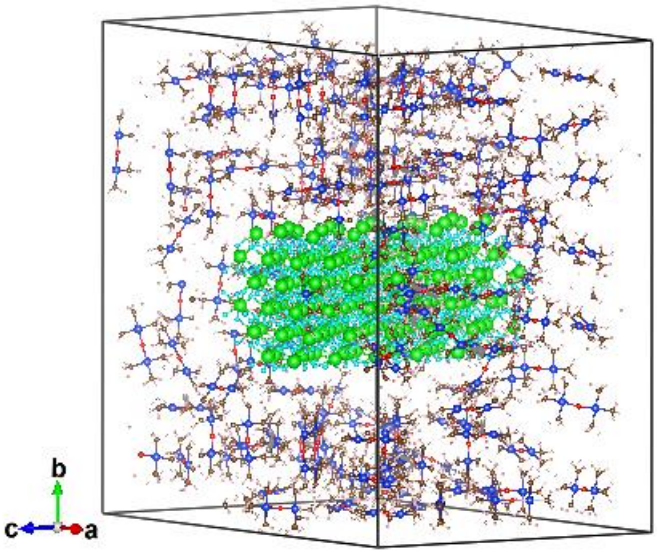

a=5.462 nm、b=5.462 nm、c= 3.55 nm

Supplementary Figure 110. CaF_2/PDMS composites with a length of 3 nm

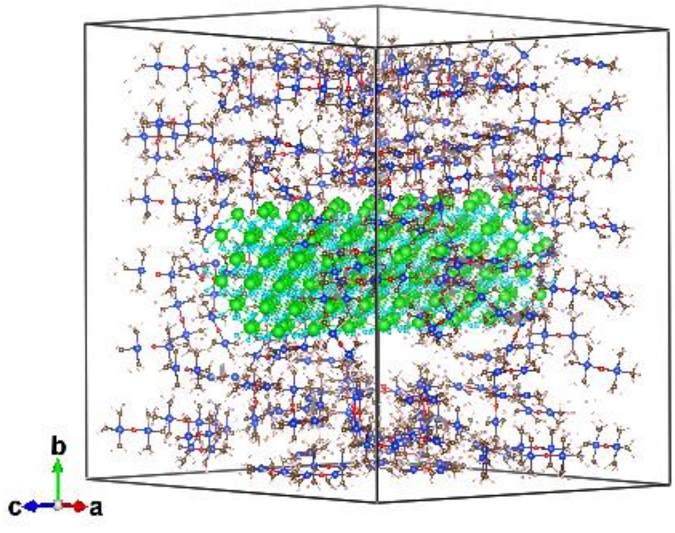

a=5.462 nm、b=5.462 nm、c= 4.55 nm

Supplementary Figure 111. CaF_2/PDMS composites with a length of 4 nm

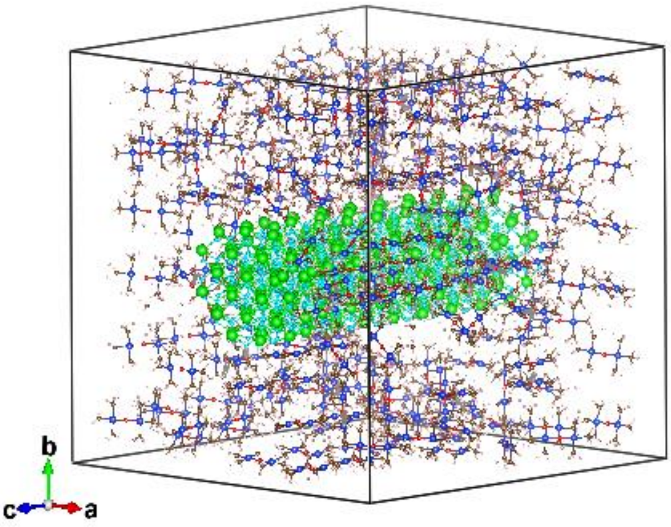

a=5.462 nm、b=5.462 nm、c= 5.55 nm

Supplementary Figure 112. CaF_2/PDMS composites with a length of 5 nm

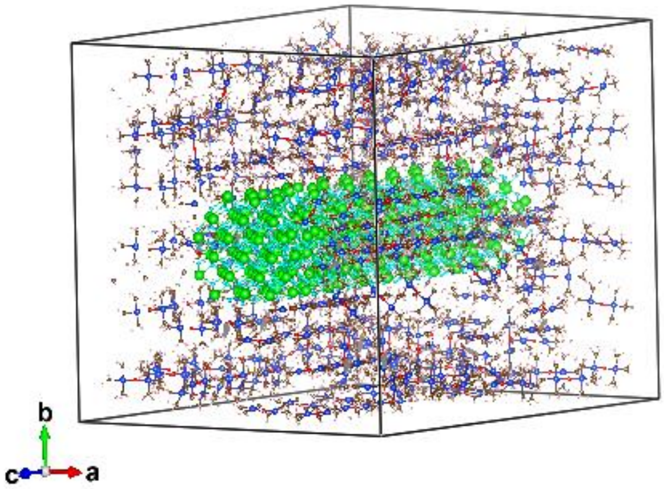

a=5.462 nm、b=5.462 nm、c= 6.55 nm

Supplementary Figure 113. CaF_2/PDMS composites with a length of 6 nm

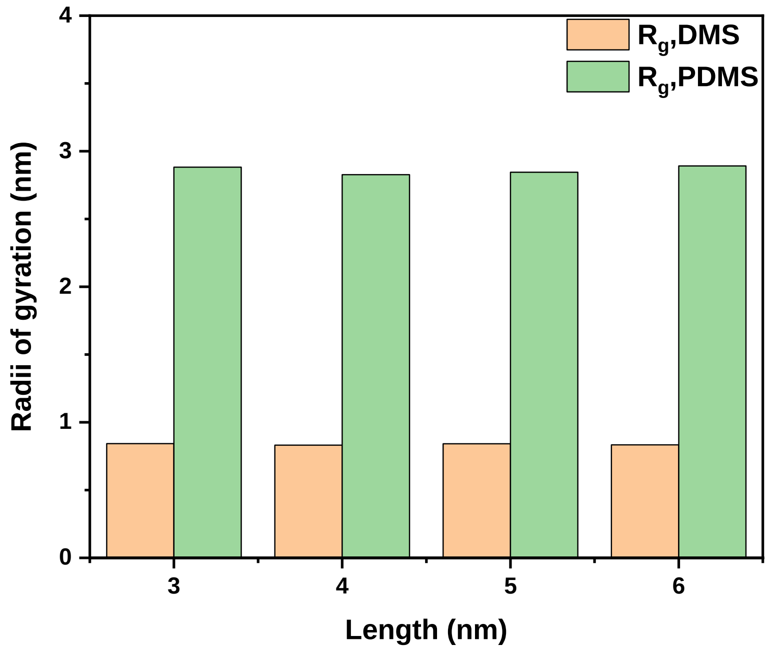

Supplementary Figure 114. Radius of gyration of CaF_2/PDMS composites dependent on length

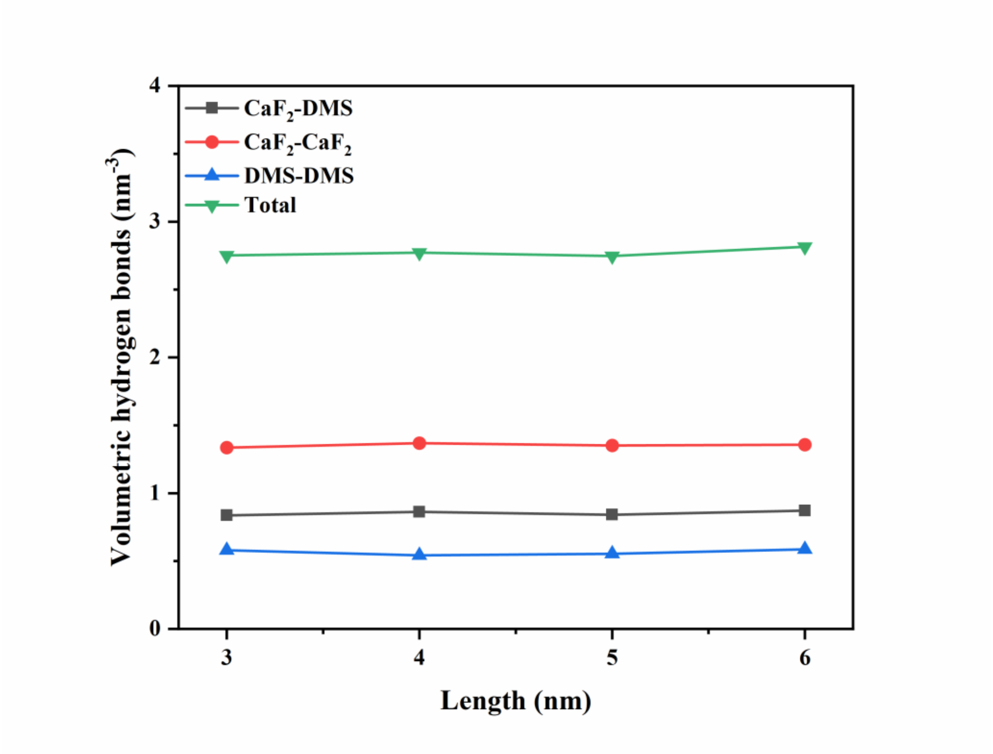

Supplementary Figure 115. Volumetric hydrogen bonds of CaF_2/PDMS composites dependent on length

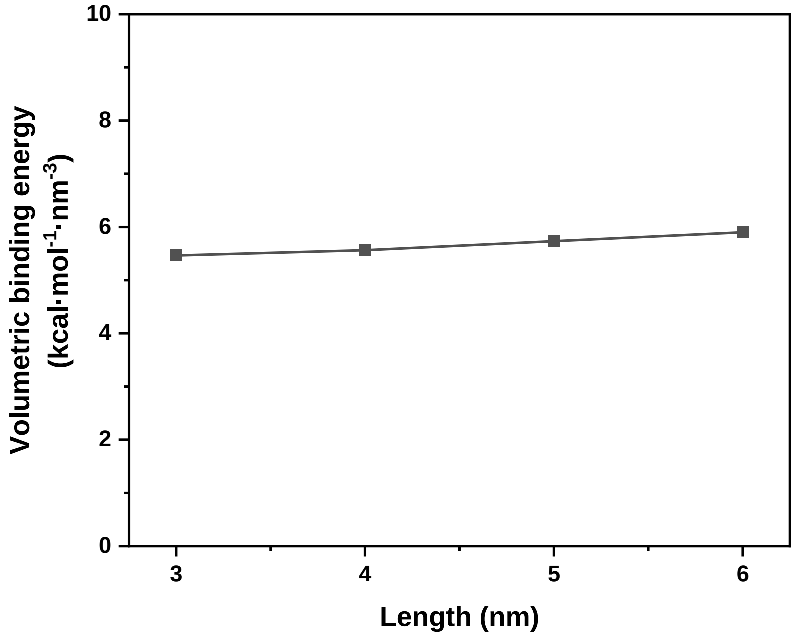

Supplementary Figure 116. Volumetric binding energy of CaF_2/PDMS composites dependent on length

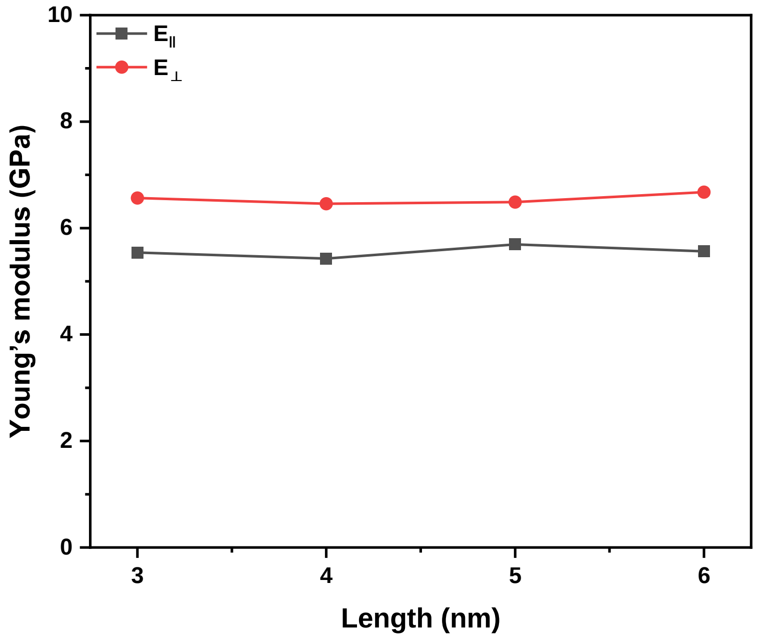

Supplementary Figure 117. Young's modulus of CaF_2/PDMS composites dependent on length

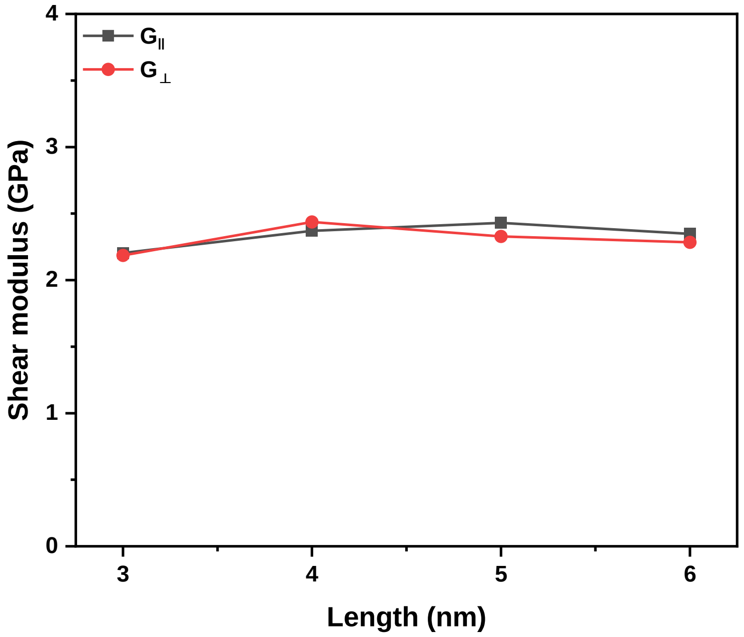

Supplementary Figure 118. Shear modulus of CaF_2/PDMS composites dependent on length

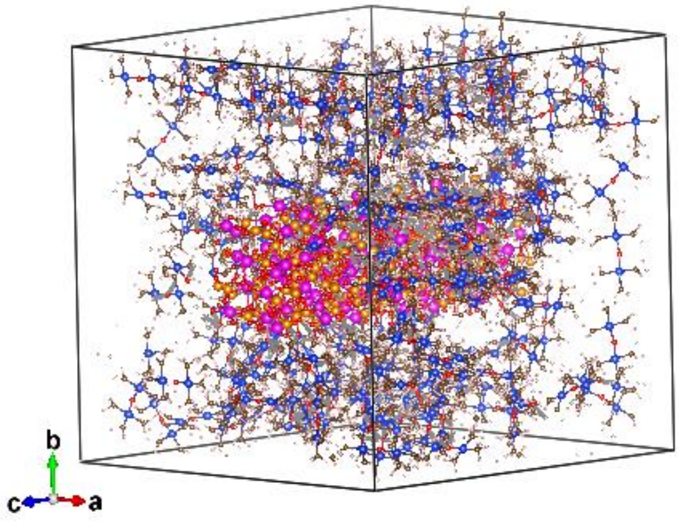

a=4.513 nm、b=4.513 nm、c= 4.55 nm

Supplementary Figure 119. $Lu_3Al_5O_{12}$/PDMS composites with a mass ratios of 10.31%

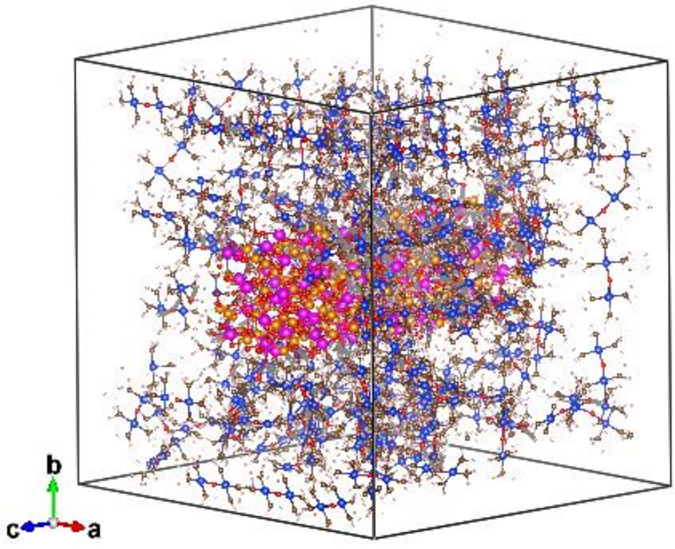

a=4.773 nm, b=4.773 nm, c= 4.55 nm

Supplementary Figure 120. $Lu_3Al_5O_{12}$/PDMS composites with a mass ratios of 13.78%

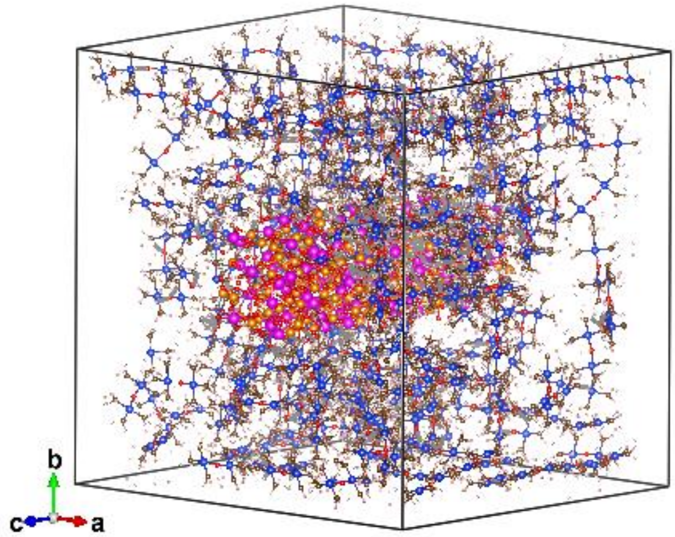

a=5.027 nm、b=5.027 nm、c= 4.55 nm

Supplementary Figure 121. $Lu_3Al_5O_{12}$/PDMS composites with a mass ratios of 16.61%

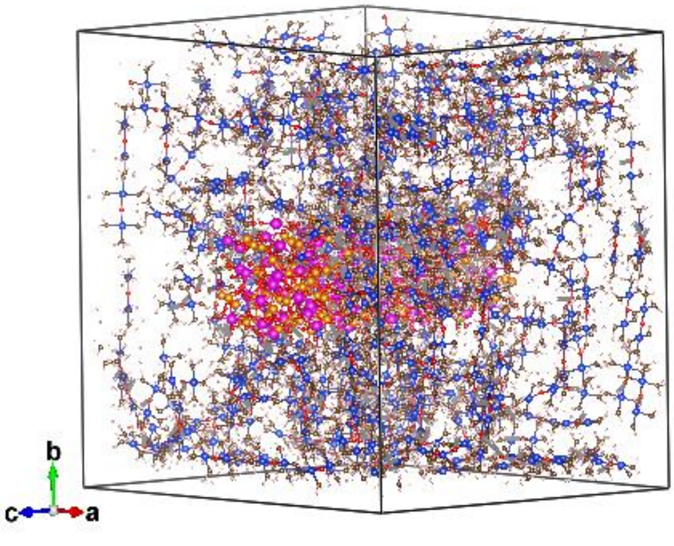

a=5.496 nm、b=5.496 nm、c= 4.55 nm

Supplementary Figure 122. $Lu_3Al_5O_{12}$/PDMS composites with a mass ratios of 20.57%

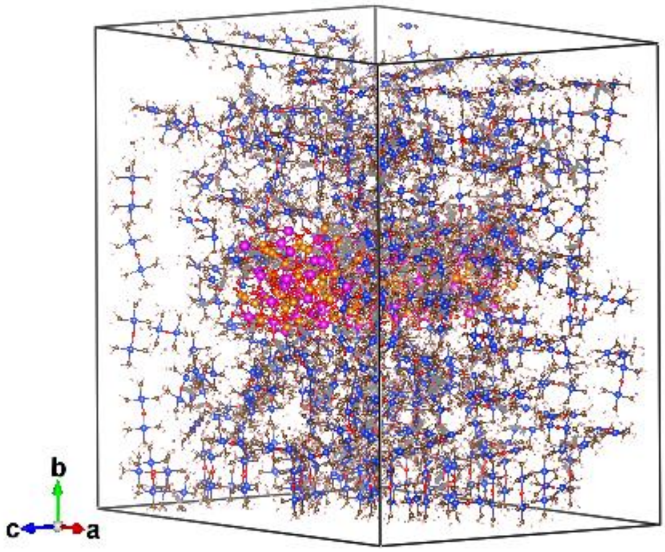

a=6.387 nm、b=6.387 nm、c= 4.55 nm

Supplementary Figure 123. $Lu_3Al_5O_{12}$/PDMS composites with a mass ratios of 22.62%

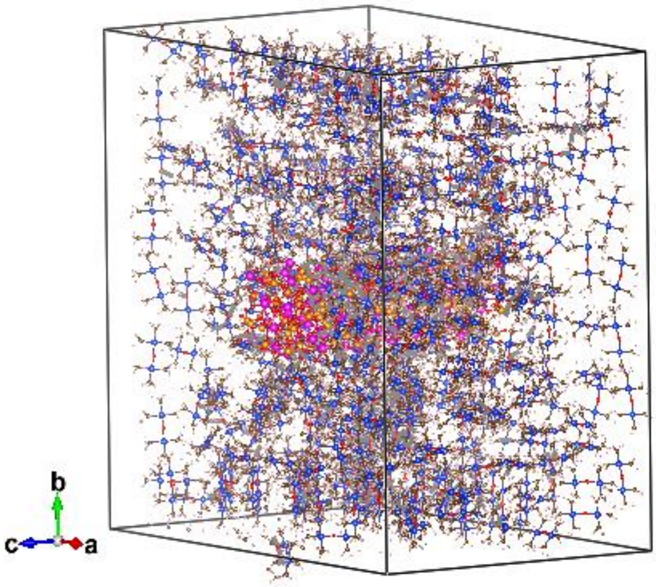

a=7.208 nm、b=7.208 nm、c= 4.55 nm

Supplementary Figure 124. $Lu_3Al_5O_{12}$/PDMS composites with a mass ratios of 25.43%

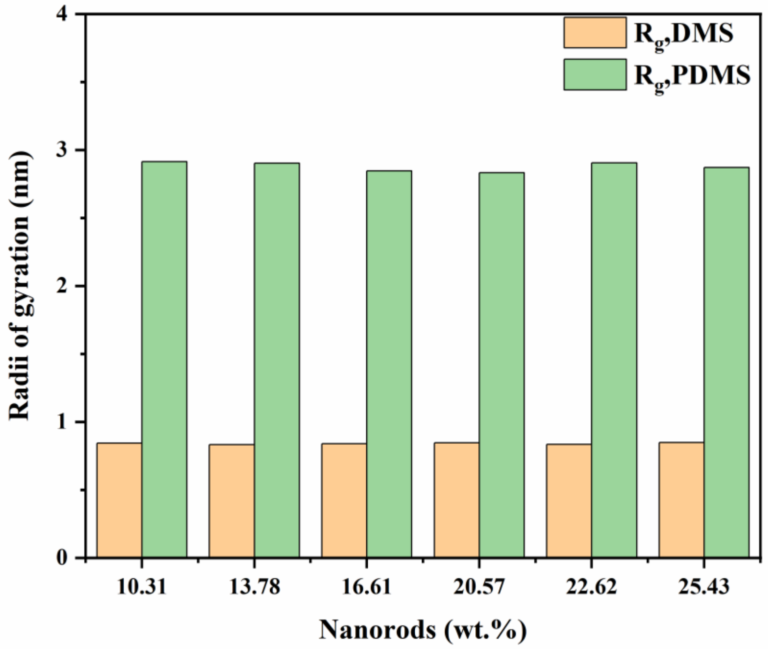

Supplementary Figure 125. Radius of gyration of $Lu_3Al_5O_{12}$/PDMS composites dependent on mass ratios

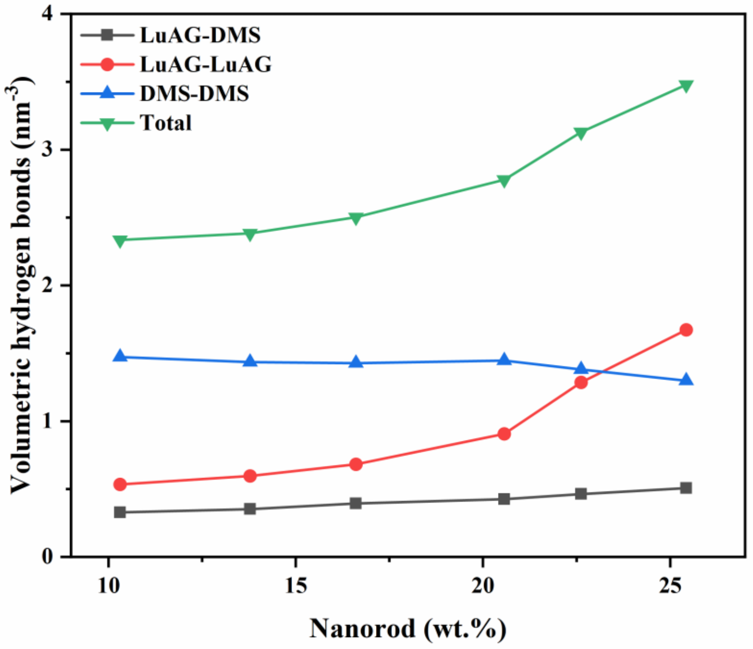

Supplementary Figure 126. Volumetric hydrogen bonds of $Lu_3Al_5O_{12}$/PDMS composites dependent on mass ratios

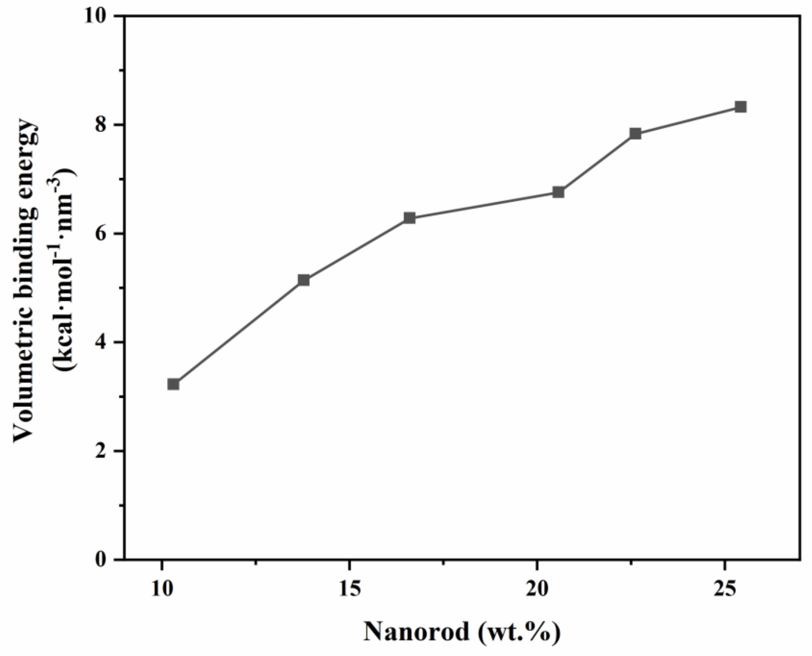

Supplementary Figure 127. Volumetric binding energy of $Lu_3Al_5O_{12}$/PDMS composites dependent on mass ratios

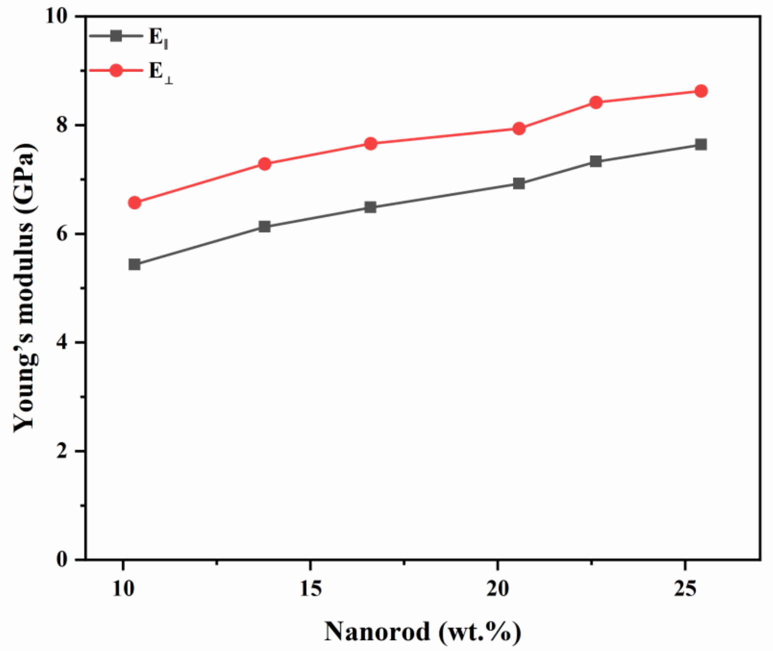

Supplementary Figure 128. Young's modulus of $Lu_3Al_5O_{12}$/PDMS composites dependent on mass ratios

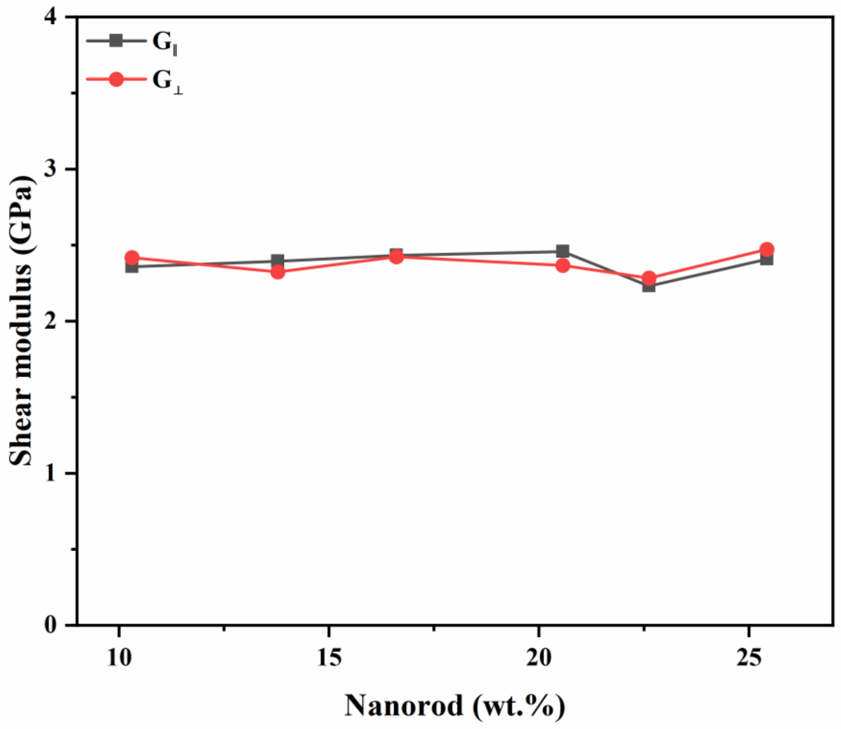

Supplementary Figure 129. Shear modulus of $Lu_3Al_5O_{12}$/PDMS composites dependent on mass ratios

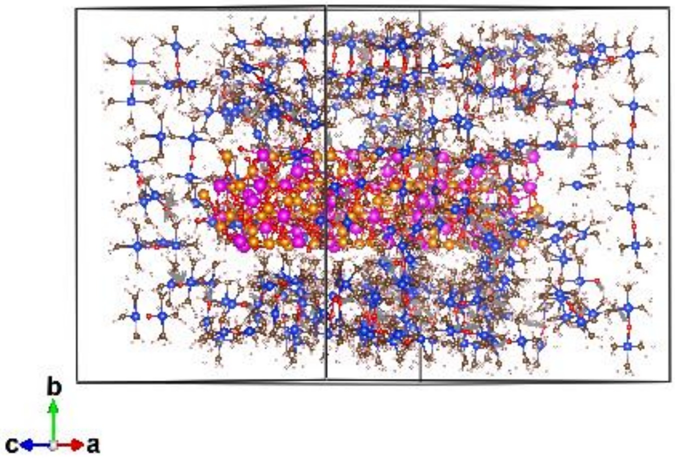

a=3.692 nm、b=3.692 nm、c= 4.55 nm

Supplementary Figure 130. $Lu_3Al_5O_{12}$/PDMS composites with a diameter of 1 nm

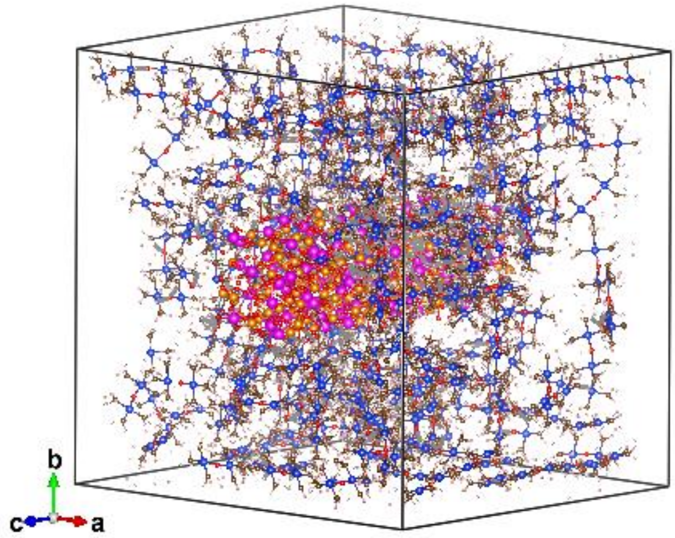

a=5.027 nm、b=5.027 nm、c= 4.55 nm

Supplementary Figure 131. $Lu_3Al_5O_{12}$/PDMS composites with a diameter of 1.5 nm

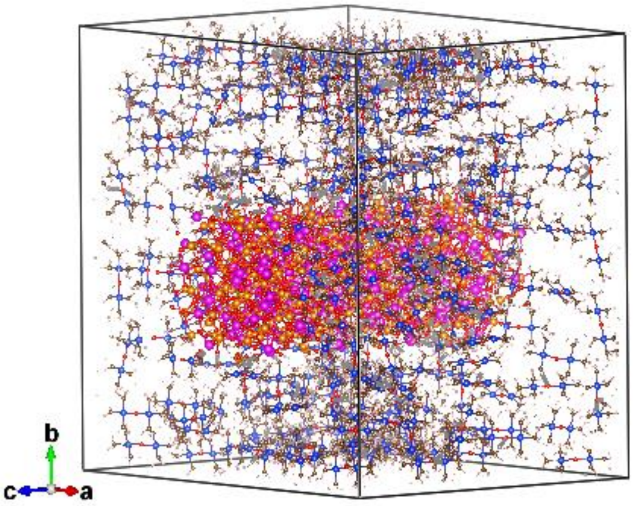

a=5.916 nm、b=5.916 nm、c= 4.55 nm

Supplementary Figure 132. $Lu_3Al_5O_{12}$/PDMS composites with a diameter of 2 nm

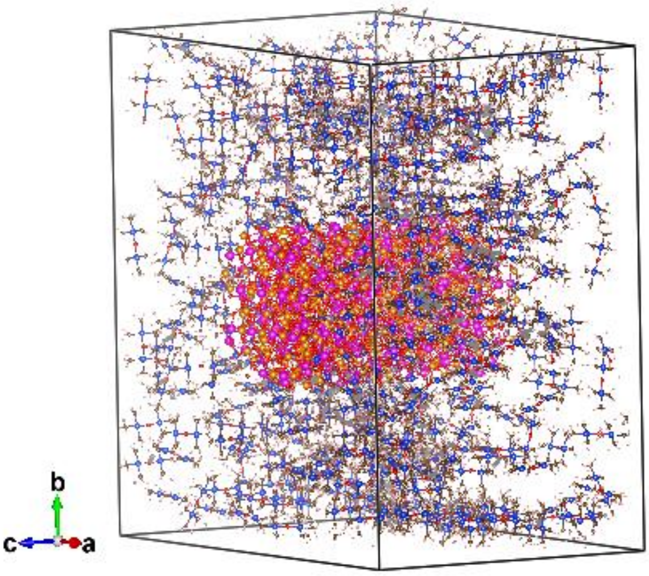

a=7.484 nm、b=7.484 nm、c= 4.55 nm

Supplementary Figure 133. $Lu_3Al_5O_{12}$/PDMS composites with a diameter of 2.5 nm

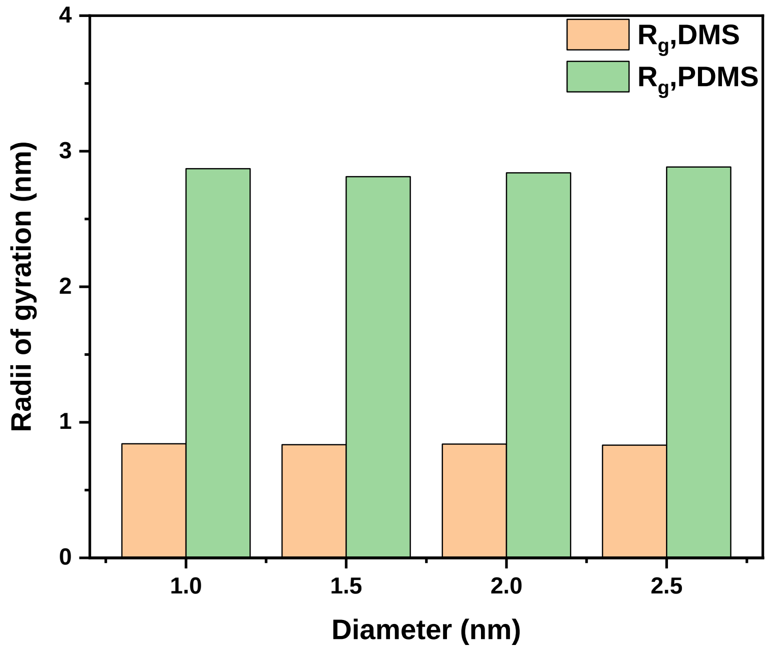

Supplementary Figure 134. Radius of gyration of $Lu_3Al_5O_{12}$/PDMS composites dependent on diameter

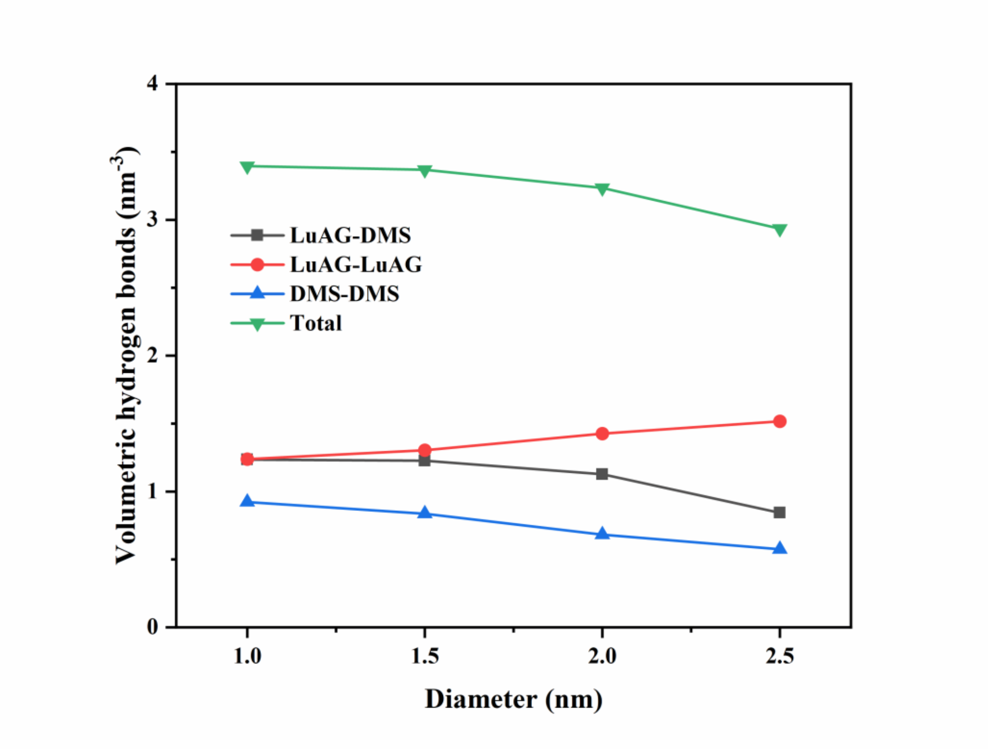

Supplementary Figure 135. Volumetric hydrogen bonds of $Lu_3Al_5O_{12}$/PDMS composites dependent on diameter

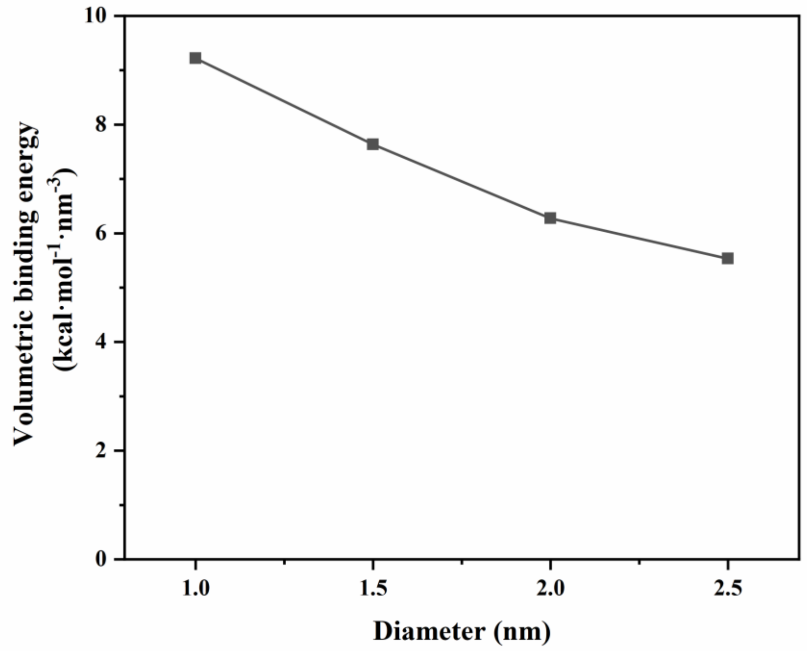

Supplementary Figure 136. Volumetric binding energy of $Lu_3Al_5O_{12}$/PDMS composites dependent on diameter

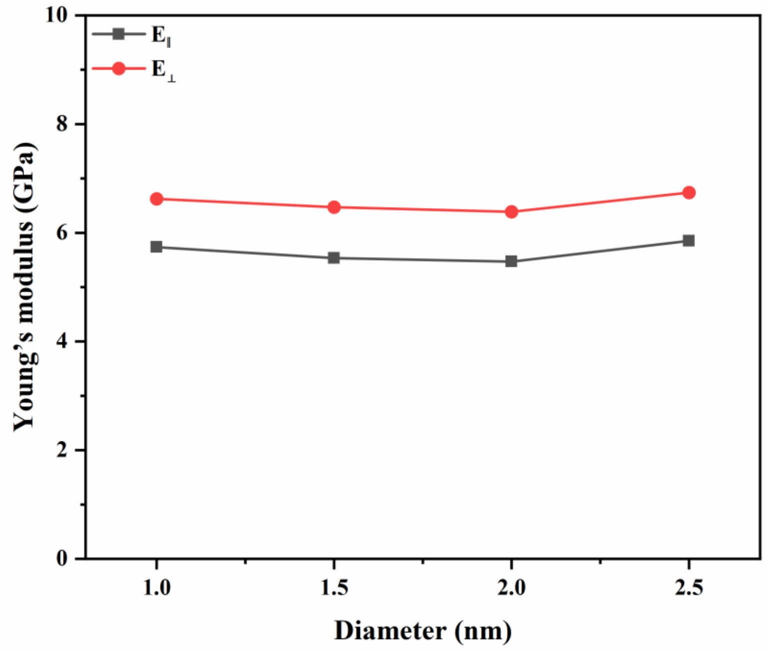

Supplementary Figure 137. Young's modulus of $Lu_3Al_5O_{12}$/PDMS composites dependent on diameter

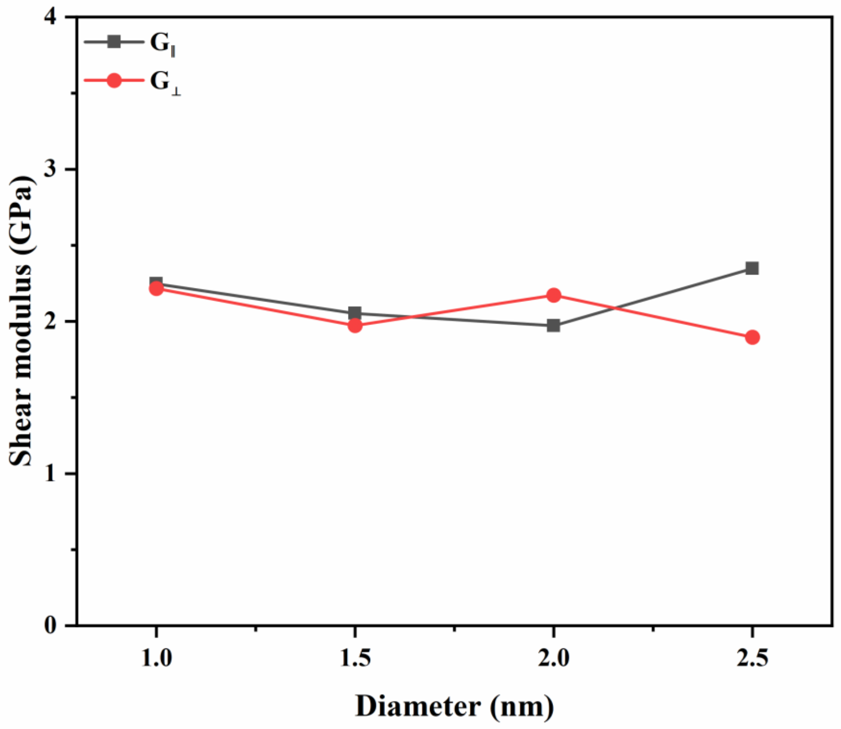

Supplementary Figure 138. Shear modulus of $Lu_3Al_5O_{12}$/PDMS composites dependent on diameter

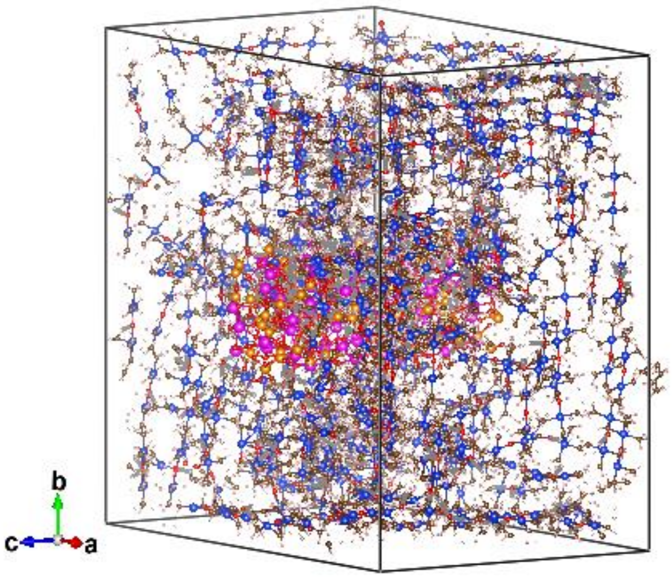

a=5.496 nm、b=5.496 nm、c= 3.55 nm

Supplementary Figure 139. $Lu_3Al_5O_{12}$/PDMS composites with a length of 3 nm

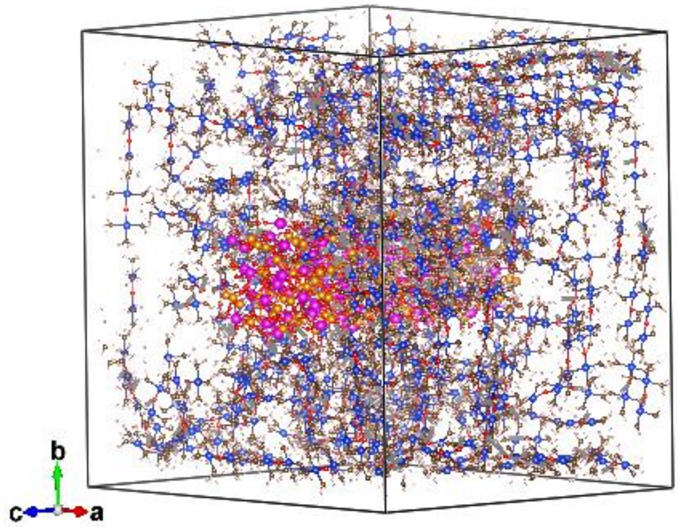

a=5.496 nm、b=5.496 nm、c= 4.55 nm

Supplementary Figure 140. $Lu_3Al_5O_{12}$/PDMS composites with a length of 4 nm

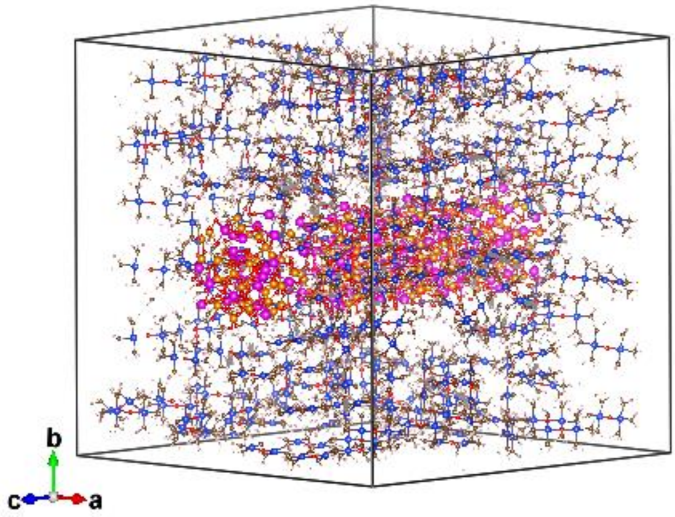

a=5.496 nm、b=5.496 nm、c= 5.55 nm

Supplementary Figure 141. $Lu_3Al_5O_{12}$/PDMS composites with a length of 5 nm

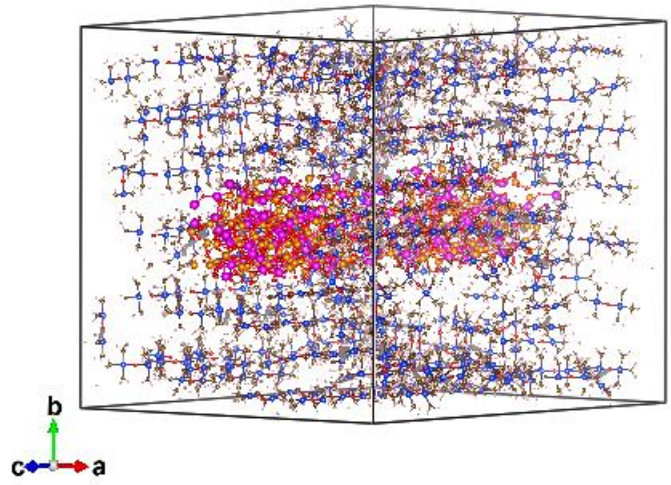

a=5.496 nm、b=5.496 nm、c= 6.55 nm

Supplementary Figure 142. $Lu_3Al_5O_{12}$/PDMS composites with a length of 6 nm

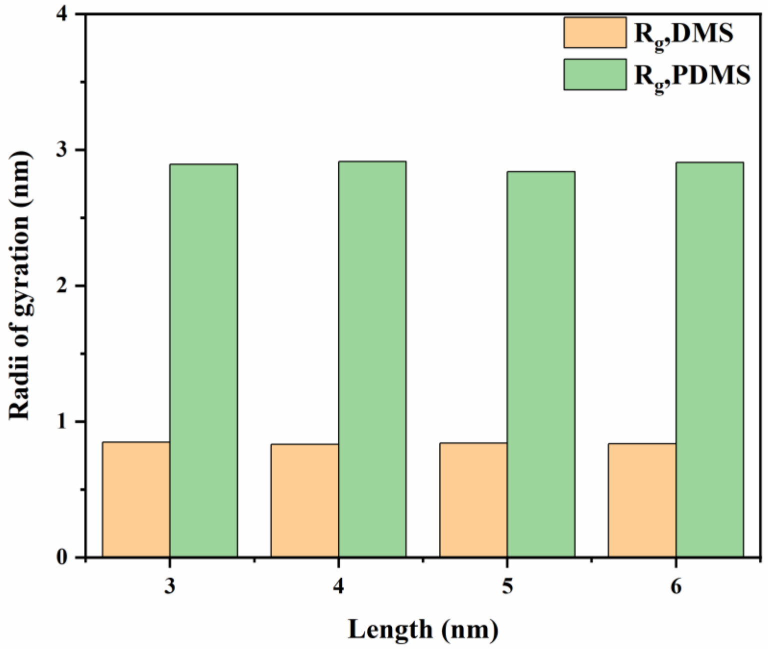

Supplementary Figure 143. Radius of gyration of $Lu_3Al_5O_{12}$/PDMS composites dependent on length

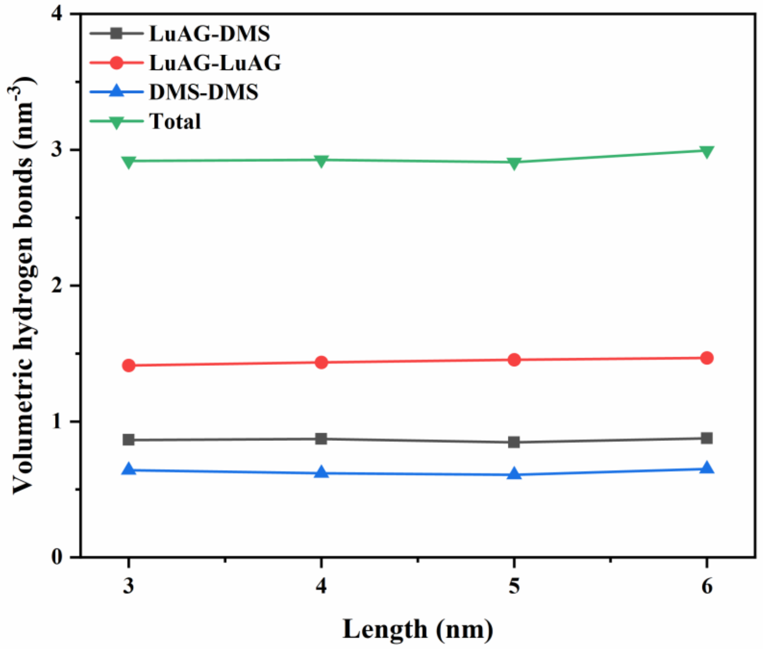

Supplementary Figure 144. Volumetric hydrogen bonds of $Lu_3Al_5O_{12}$/PDMS composites dependent on length

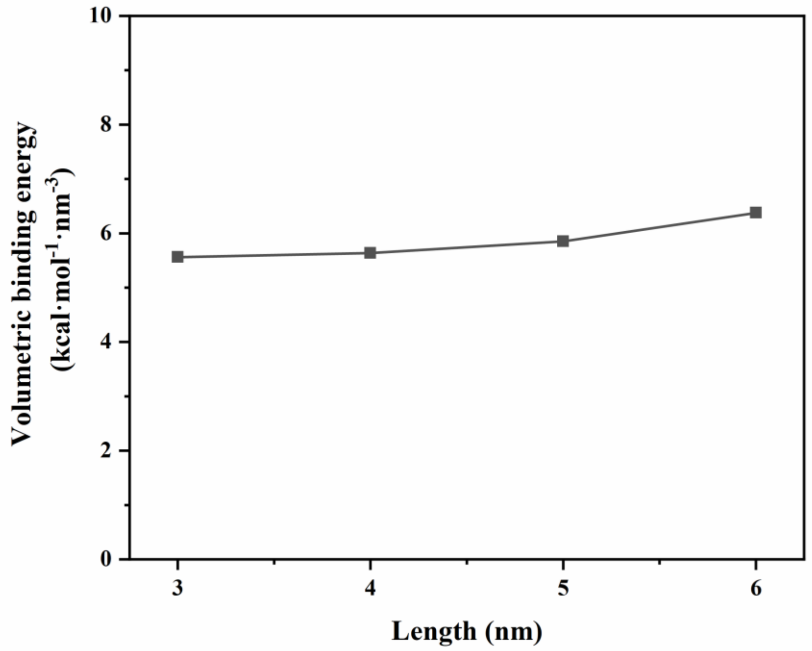

Supplementary Figure 145. Volumetric binding energy of $Lu_3Al_5O_{12}$/PDMS composites dependent on length

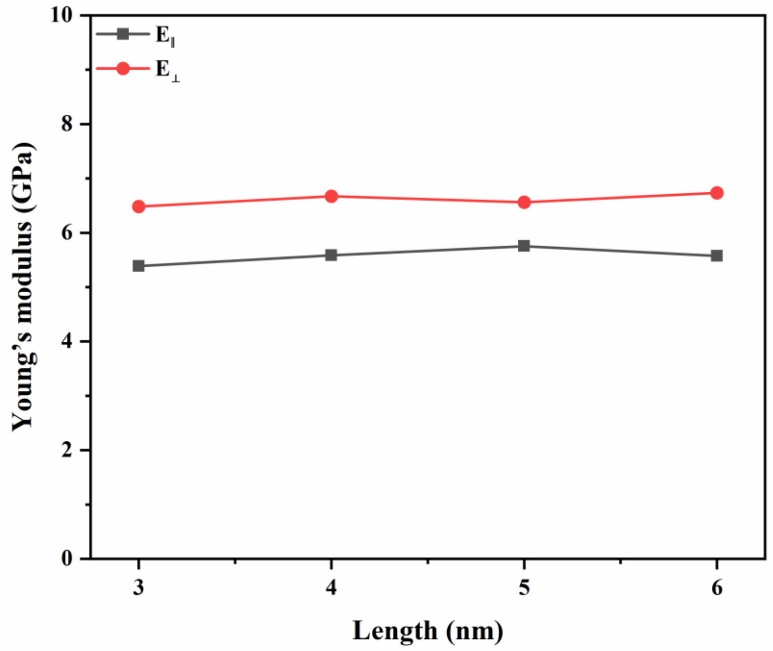

Supplementary Figure 146. Young's modulus of $Lu_3Al_5O_{12}$/PDMS composites dependent on length

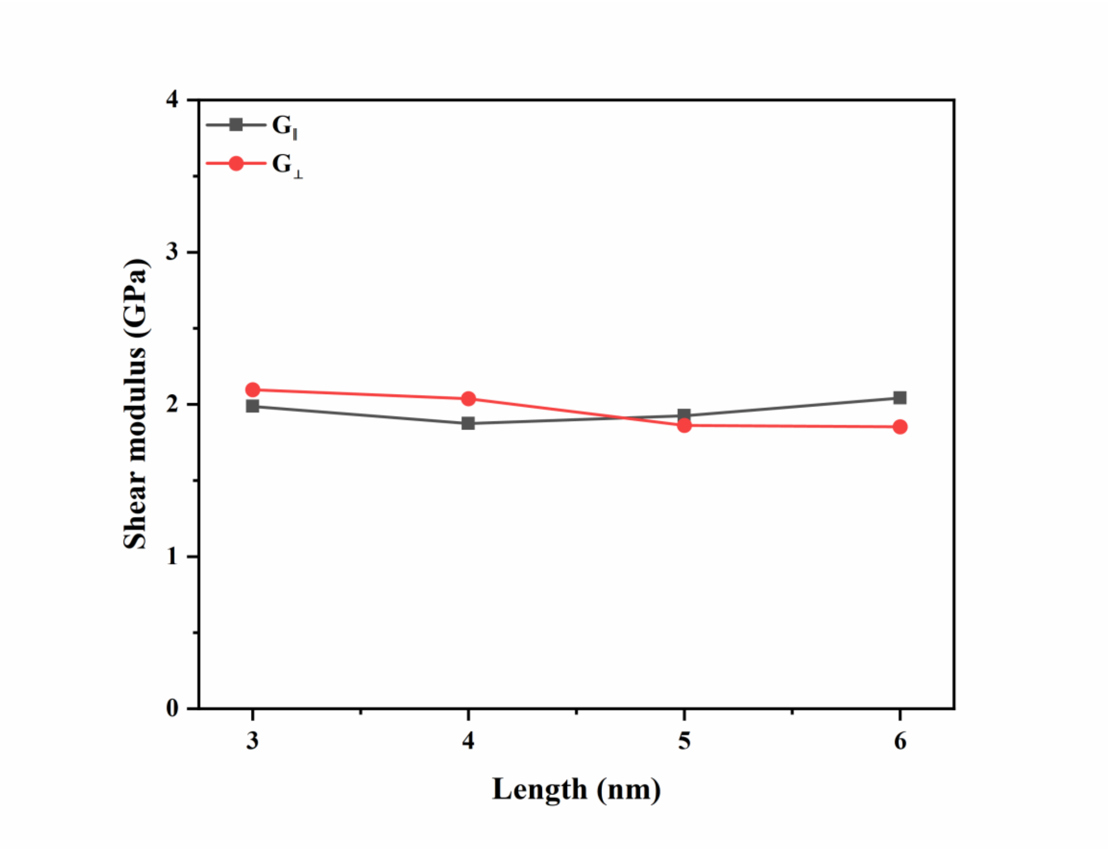

Supplementary Figure 147. Shear modulus of $Lu_3Al_5O_{12}$/PDMS composites dependent on length

Supplementary Note 18: A brief introduction to the AFM and EFM techniques

Generally, the surface morphology is measured by an atomic force microscope (AFM) and the surface electron distribution can be measured by an electrostatic force microscope (EFM). In this work, both the surface morphology and surface electron distribution of the samples were obtained by using an Oxford Cypher S AFM Microscope. AFM is a broader technique that encompasses various force interactions, while EFM is a specialized mode of AFM that specifically investigates electrostatic forces. The principle of AFM is based on the detection of forces, primarily the van der Waals forces, between the atoms on the surface of the sample and the atoms on the probe tip. These forces cause the deflection of the cantilever, which is monitored using a laser beam deflection or an array of piezoresistive sensors. By precisely controlling the tip-sample distance, the topography of the surface can be mapped with high resolution. EFM is a specialized mode of AFM that focuses on the measurement of electrostatic forces between the probe tip and the sample surface. It provides information about the local electrical properties and surface charge distribution of the sample.

Supplementary References

1. Wu, C. *et al.* Efficient Mechanoluminescent Elastomers for Dual-Responsive Anticounterfeiting Device and Stretching/Strain Sensor with Multimode Sensibility. *Adv. Funct. Mater.* **28**, 1803168 (2018).
2. Ma, Z. *et al.* Mechanics-induced triple-mode anticounterfeiting and moving tactile sensing by simultaneously utilizing instantaneous and persistent mechanoluminescence. *Mater. Horiz.* **6**, 2003–2008 (2019).
3. Zhou, J. *et al.* An ultra-strong non-pre-irradiation and self-recoverable mechanoluminescent elastomer. *Chem. Eng. J.* **390**, 124473 (2020).
4. Wang, J. *et al.* Contact Electrification Induced Multicolor Self-Recoverable Mechanoluminescent Elastomer for Wearable Smart Light-Emitting Devices. *Adv. Opt. Mater.* **11**, 2203112 (2023).
5. Zhang, X. *et al.* Ultralong UV/mechano-excited room temperature phosphorescence from purely organic cluster excitons. *Nat. Commun.* **10**, 5161 (2019).
6. Zhang, Q. *et al.* A flexible organic mechanoluminophore device. *Nat. Commun.* **14**, 1257 (2023).
7. Wang, W. *et al.* Contact electrification induced mechanoluminescence. *Nano Energy* **94**, 106920 (2022).
8. Zou, H. *et al.* Quantifying the triboelectric series. *Nat. Commun.* **10**, 1427 (2019).
9. Briegleb, G. Electron Affinity of Organic Molecules. *Angew. Chem. Int. Ed. Engl.* **3**, 617–632 (1964).
10. Politzer, P., Murray, J. S. & Clark, T. Halogen bonding: an electrostatically-driven highly directional noncovalent interaction. *Phys. Chem. Chem. Phys.* **12**, 7748–7757 (2010).
11. Pritchard, H. O. & Skinner, H. A. The Concept Of Electronegativity. *Chem. Rev.* **55**, 745–786 (1955).
12. Wang, S., Lin, L. & Wang, Z. L. Nanoscale Triboelectric-Effect-Enabled Energy Conversion for Sustainably Powering Portable Electronics. *Nano Lett.* **12**, 6339–6346 (2012).
13. Bae, J. *et al.* Flutter-driven triboelectrification for harvesting wind energy. *Nat. Commun.* **5**, 4929 (2014).
14. Wang, Z. L. & Wang, A. C. On the origin of contact-electrification. *Mater. Today* **30**,

34–51 (2019).

15. Liu, Z. *et al.* Fabrication of triboelectric polymer films via repeated rheological forging for ultrahigh surface charge density. *Nat. Commun.* **13**, 4083 (2022).
16. Momma, K. & Izumi, F. VESTA 3 for three-dimensional visualization of crystal, volumetric and morphology data. *J. Appl. Crystallogr.* **44**, 1272–1276 (2011).
17. Wang, V., Xu, N., Liu, J.-C., Tang, G. & Geng, W.-T. VASPKIT: A user-friendly interface facilitating high-throughput computing and analysis using VASP code. *Comput. Phys. Commun.* **267**, 108033 (2021).
18. Perdew, J. P., Burke, K. & Ernzerhof, M. Generalized gradient approximation made simple. *Phys. Rev. Lett.* **77**, 3865 (1996).
19. Kresse, G. & Joubert, D. From ultrasoft pseudopotentials to the projector augmented-wave method. *Phys. Rev. B* **59**, 1758 (1999).
20. Kresse, G. & Furthmüller, J. Efficiency of ab-initio total energy calculations for metals and semiconductors using a plane-wave basis set. *Comp. Mater. Sci.* **6**, 15–50 (1996).
